# Wuchuyuamines A-G, seven new alkaloids isolated from the fruits of *Tetradium ruticarpum* and their antifungal activities

**DOI:** 10.1007/s13659-026-00610-6

**Published:** 2026-08-10

**Authors:** Meng-Ting Chen, Jian Zhang, Qian Zhao, Su-Min Zhang, Yin-Ling Wei, Ying Zhang, Xiao-Jiang Hao

**Affiliations:** 1 State Key Laboratory of Phytochemistry and Natural Medicines, Kunming Institute of Botany, Chinese Academy of Sciences, Kunming, 650201 China; 2 Yunnan Characteristic Plant Extraction Laboratory, Kunming, 650106 China; 3 University of Chinese Academy of Sciences, Beijing, 100049 China; 4 Research Unit of Chemical Biology of Natural Anti-Virus Products, Chinese Academy of Medical Sciences, Beijing, 100730 China

**Keywords:** *Tetradium ruticarpum*, Alkaloids, Antifungal activity, *Fusarium graminearum*

## Abstract

Plant diseases caused by fungal pathogens severely threaten crop yields and food security, driving the need for new, environmentally friendly fungicides. In this study, thirty-six alkaloids, including seven novel ones (**1**–**4**, **23**, **29**, and **30**), were isolated from the fruits of *Tetradium ruticarpum*, and their structures were elucidated by extensive spectroscopic analyses. The antifungal potential of compounds obtained with sufficient quantities was evaluated using the mycelial growth rate method against three phytopathogens (*Alternaria solani*, *Bipolaris sorokiniana*, and *Fusarium odoratissimum*). Compounds **16** and **24** showed notable activity against *A. solani*, exhibiting approximately 80% and 90% growth inhibition at 100 μg/mL, with IC_50_ values of 33.39 and 36.02 μg/mL, respectively. To evaluate the antifungal spectrum, the abundant constituents **13**, **19**, and **25** were further screened against sixteen phytopathogenic fungi. Among them, compound **25** displayed the most potent and broad-spectrum antifungal activity, achieving an IC_50_ of 13.15 μg/mL against *Fusarium graminearum*, which was stronger than the positive control hymexazol. Furthermore, SEM and TEM analyses revealed that compound **25** markedly disrupted the surface morphology and cellular ultrastructure, suggesting a potential link to membrane integrity impairment. Additionally, leakage assays showed increased release of nucleic acids and proteins, providing evidence that compound **25** increased membrane permeability and compromised the membrane integrity in *F. graminearum*. Therefore, compound **25** represents a promising lead structure for the development of novel, eco-friendly fungicides.

**Graphical Abstract:**

**Figure:**
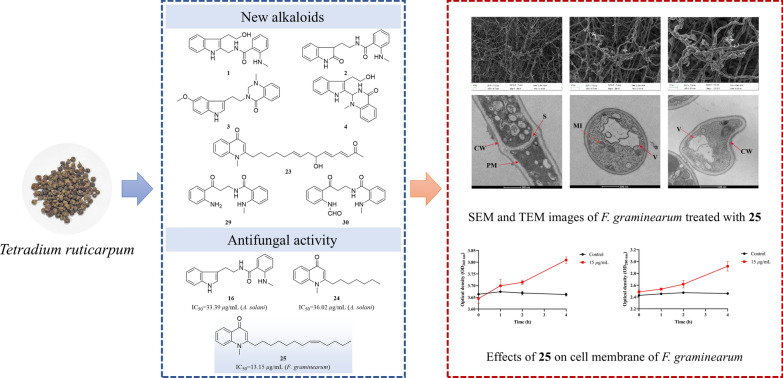

**Supplementary Information:**

The online version contains supplementary material available at 10.1007/s13659-026-00610-6.

## Introduction

Plant pathogens cause a wide range of diseases in fruits, vegetables, and other crops, resulting in substantial yield losses and posing a major threat to global agricultural production and food security. Approximately 8000 species of fungi and oomycetes have been reported to be associated with plant diseases [1, 2]. Among fungal pathogens, *Magnaporthe oryzae*, *Botrytis cinerea*, *Puccinia* spp., *Fusarium graminearum*, and *Fusarium oxysporum* are the most scientifically and economically important fungal pathogens [3]. Chemical control remains the most widely used approach for managing plant diseases. However, the excessive and long-term application of synthetic fungicides has led to multiple problems, including environmental pollution, potential health risks to humans and animals, and the emergence of fungicide-resistant pathogens [4]. These issues have prompted an urgent demand for safer and more sustainable alternatives.

To meet this demand, the development of plant-derived natural products as sources of fungicidal agents is consistent with future-oriented food and agricultural policies [5]. Among these natural products, alkaloids, a class of nitrogen-containing heterocyclic compounds, have garnered increasing attention due to their relatively low mammalian toxicity, ready biodegradability, and environmental compatibility [6, 7]. *Tetradium ruticarpum* (A. Juss.) T. G. Hartley (Rutaceae family, *Tetradium* genus), known as Wu Zhu Yu in traditional Chinese medicine, has been widely used for centuries to treat ailments such as headache, abdominal pain, and vomiting [8]. Phytochemical investigations have revealed that *T. ruticarpum* contains diverse bioactive constituents, including alkaloids, limonoids, and flavonoids [9–11]. To date, approximately 300 metabolites have been reported in *T. ruticarpum*, about half of which are alkaloids [12]. Pharmacological studies further indicate that these constituents exhibit diverse biological activities, such as vasodilatory, anti-inflammatory, analgesic, and antibacterial effects [13, 14]. Nevertheless, the antifungal potential of alkaloids derived from *T. ruticarpum* remains largely unexplored.

**Fig. 1:**
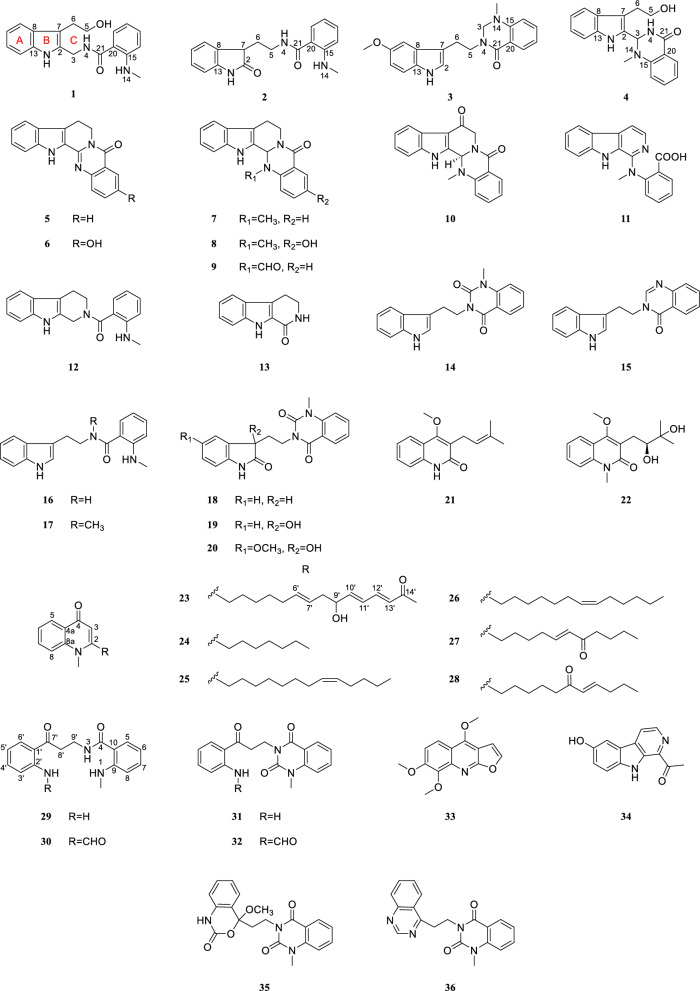
The structures of compounds **1**–**36**

In this study, we aimed to systematically isolate and identify the alkaloids from the fruits of *T. ruticarpum* and evaluate their antifungal activities against phytopathogenic fungi. A total of thirty-six alkaloids were obtained (Fig. 1), including seven new ones (**1**–**4**, **23**, **29**, and **30**) and twenty-nine known alkaloids: rutaecarpine (**5**) [15], 3-hydroxyrutaecarpine (**6**) [16], evodiamine (**7**) [15], 3-hydroxyevodiamine (**8**) [17], *N*^14^-formyldihydrorutaecarpine (**9**) [18], evodamide A (**10**) [19], evollionines A (**11**) [20], goshuyuamide I (**12**) [21], 1,2,3,4-tetrahydronorharman-1-one (**13**) [22], goshuyuamide II (**14**) [21], 3-(2-(1H-indol-3-yl)ethyl)quinazolin-4(3H)-one (**15**) [23],* N*-(2-methylaminobenzoyl)tryptamine (**16**) [24], evodiamide (**17**) [25], wuchuyuamide II (**18**) [26], wuchuyuamide I (**19**) [26], evollionines B (**20**) [20], atanine (**21**) [27], edulinine (**22**) [28], 2-heptyl-1-methyl-4(1*H*)-quinolinone (**24**) [29], evocarpine (**25**) [30], 1-methyl-2-[(*Z*)-7-tridecenyl]-4(1*H*)-quinolinone (**26**) [31], euocarpine A (**27**) [32], euocarpine B (**28**) [32], wuchuyuamide III (**31**) [33], wuchuyuamide IV (**32**) [34], skimmianine (**33**) [35], sinometumine I (**34**) [36], evodiamide A (**35**) [37], and evodiamide B (**36**) [37]. Their structures were elucidated based on extensive spectroscopic analyses. Compounds **8**, **15**, **22**, and **34** were reported from *T. ruticarpum* for the first time. This report described the isolation and structural elucidation of all alkaloids obtained from *T. ruticarpum*, and the antifungal activities of selected compounds were further assessed.

## Results and discussion

### Structure elucidation and identification

**Fig. 2:**
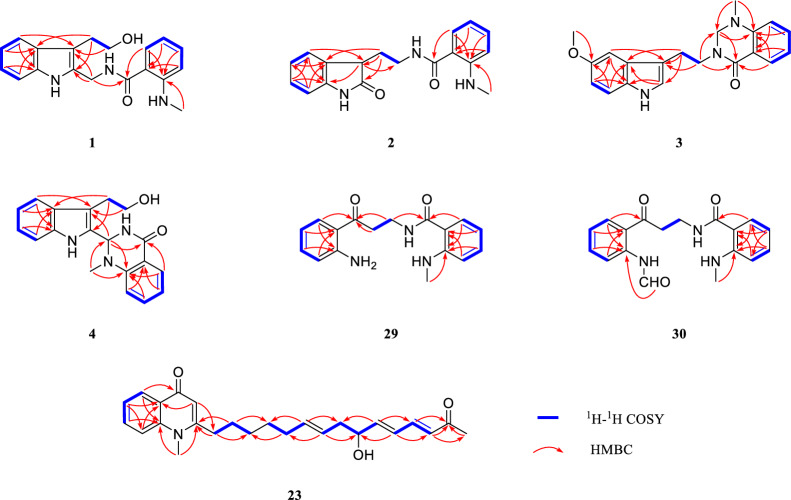
Key ^1^H–^1^H COSY and HMBC correlations of compounds **1**–**4**, **23**, **29**, and **30**

Wuchuyuamine A (**1**) was acquired as a yellow amorphous powder and its molecular formula C_19_H_21_N_3_O_2_ was determined by HRESIMS at *m/z* 346.1533 [M + Na]^+^ (calcd. for C_19_H_21_N_3_O_2_Na, 346.1526) with 11 degrees of unsaturation. The ^1^H NMR data (CD_3_OD, 500 MHz; Table 1) of compound **1** revealed two 1,2-disubstituted aromatic rings [*δ*_H_ 7.49 (1H, d, *J* = 7.8 Hz, H-9), *δ*_H_ 6.98 (1H, ddd, *J* = 8.0, 7.0, 1.1 Hz, H-10), *δ*_H_ 7.06 (1H, ddd, *J* = 8.1, 7.1, 1.2 Hz, H-11), *δ*_H_ 7.29 (1H, m, H-12);* δ*_H_ 6.68 (1H, d, *J* = 8.4 Hz, H-16), *δ*_H_ 7.29 (1H, m, H-17), *δ*_H_ 6.56 (1H, dd, *J* = 8.0, 7.0 Hz, H-18),* δ*_H_ 7.46 (1H, dd, *J* = 7.9, 1.6 Hz, H-19)], three aliphatic methylene signals [*δ*_H_ 4.66 (2H, s, H-3), *δ*_H_ 3.78 (2H, t, *J* = 6.8 Hz, H-5),* δ*_H_ 3.03 (2H, t, *J* = 6.8 Hz, H-6)], and an *N*-CH_3_ signal [*δ*_H_ 2.83 (3H, s, *N*-CH_3_)]. In addition, the ^13^C NMR, DEPT, and HSQC data showed seven quaternary carbon signals, including six aromatic carbon signals [*δ*_C_ 133.9 (C-2), 110.4 (C-7), 129.4 (C-8), 137.4 (C-13), 151.6 (C-15), 116.9 (C-20)] and an amide carbon signal [*δ*_C_ 172.2 (C-21)]. These data indicated that compound **1** was an indolequinazoline alkaloid. The structure could be determined through further 2D NMR analysis. In the ^1^H-^1^H COSY spectrum, the cross peak of H_2_-5/H_2_-6 confirmed the presence of a CH_2_CH_2_ alkyl fragment. The HMBC correlations from H_2_-5 to C-7 and from H_2_-6 to C-2/C-7/C-8 indicated that C-7 on the indole ring was connected to the CH_2_CH_2_ alkyl fragment. The HMBC correlations from H_2_-3 to C-2/C-7/C-21 demonstrated that the quinazoline unit was connected to the indole ring through C-3 (Fig. 2). Along with the molecular formula of compound **1** and the chemical shift of C-5 (*δ*_C_ 63.4) suggested that C-5 was connected to a hydroxyl group. Comparing with the structure of goshuyuamide I (**12**) [21], the notable difference was that **1** was a C-*seco* indolequinazoline alkaloid with a 5-hydroxyl group. Therefore, the structure of compound **1** was established as depicted in Fig. 1.

**Table 1:** ^1^H NMR and ^13^C NMR spectroscopic data for **1**–**4** (*δ* in ppm, *J* in Hz)

No	**1**^a^		**2**^b^		**3**^c^		**4**^a^	
	*δ*_H_	*δ*_C_	*δ*_H_	*δ*_C_	*δ*_H_	*δ*_C_	*δ*_H_	*δ*_C_
2		133.9		179.8	7.08 (1H, s)	124.5		132.4
3	4.66 (2H, s)	36.0			4.21 (2H, s)	68.1	5.98 (1H, s)	67.9
5	3.78 (2H, t, 6.8)	63.4	3.58 (2H, m)	37.8	3.80 (2H, t, 7.0)	48.5	3.80 (2H, m)	63.3
6	3.03 (2H, t, 6.8)	28.5	2.18 (2H, m)	31.3	3.08 (2H, t, 7.0)	25.0	3.06 (2H, m)	28.4
7		110.4	3.53 (1H, d, 6.0)	44.8		113.0		113.9
8		129.4		130.7		129.1		128.8
9	7.49 (1H, d, 7.8)	119.2	7.40 (1H, d, 7.4)	125.1	7.09 (1H, d, 2.4)	101.2	7.58 (1H, d, 8.0)	119.8
10	6.98 (1H, ddd, 8.0, 7.0, 1.1)	119.7	7.00 (1H, dd, 7.6, 7.5)	122.5		155.0	7.04 (1H, ddd, 8.0, 6.9, 1.0)	120.1
11	7.06 (1H, ddd, 8.1, 7.1, 1.2)	122.4	7.20 (1H, dd, 7.8, 7.6)	128.6	6.74 (1H, dd, 8.7, 2.4)	112.9	7.14 (1H, ddd, 8.2, 7.0, 1.1)	123.7
12	7.29 (1H, m)	112.0	6.92 (1H, d, 7.7)	110.2	7.22 (1H, d, 8.7)	112.9	7.36 (1H, d, 8.2)	112.6
13		137.4		143.5		133.3		138.1
15		151.6		151.7		151.2		151.0
16	6.68 (1H, d, 8.4)	112.0	6.64 (1H, d, 8.3)	111.4	6.72 (1H, d, 8.4)	113.3	6.91 (1H, m)	114.0
17	7.29 (1H, m)	133.8	7.27 (1H, dd, 8.6, 7.1)	133.2	7.40 (1H, dd, 8.3, 7.2)	134.9	7.48 (1H, dd, 7.3, 1.7)	135.8
18	6.56 (1H, dd, 8.0, 7.0)	115.9	6.52 (1H, dd, 7.9, 7.1)	114.8	6.86 (1H, dd, 7.5, 7.5)	119.6	6.91 (1H, m)	119.5
19	7.46 (1H, dd, 7.9, 1.6)	129.3	7.54 (1H, d, 7.9)	128.6	7.83 (1H, d, 7.7)	129.3	7.88 (1H, dd, 7.9, 1.7)	129.2
20		116.9		116.1		118.6		117.2
21		172.2		170.4		165.6		166.4
*N*-CH_3_	2.83 (3H, s)	29.9	2.82 (3H, s)	30.0	2.63 (3H, s)	35.6	2.65 (3H, s)	34.2
OCH_3_					3.72 (3H, s)	56.2		

^a^500 MHz in CD_3_OD; 125 MHz in CD_3_OD

^b^600 MHz in (CD_3_)_2_CO; 150 MHz in (CD_3_)_2_CO

^c^600 MHz in CD_3_OD; 150 MHz in CD_3_OD

Wuchuyuamine B (**2**) was obtained as a white amorphous powder. Its molecular formula was determined as C_18_H_19_N_3_O_2_ based on HRESIMS ion peak at *m/z* 310.1547 [M + H]^+^ (calcd. for C_18_H_20_N_3_O_2_, 310.1550). The ^1^H NMR (CD_3_OD, 600 MHz; Figure S12.1) signals of compound **2** were significantly broadened, rendering the spin–spin coupling constants unmeasurable and thereby complicating the structural analysis. Therefore, derivatization of compound **2** was attempted by using chemical approach with hydrogen chloride-methanol solution. After converting **2** into the wuchuyuamine B chloride, the signal broadening disappeared, and coupling constants could be easily measured. The 1D NMR data (^1^H NMR, 600 MHz; ^13^C NMR, 150 MHz), recorded in (CD_3_)_2_CO (see Table 1), indicated that compound **2** had a high degree of structural similarity to *N*-(2-methylaminobenzoyl)tryptamine (**16**) [24]. However, compound **2** had an indolinone unit rather than an indoline unit due to the signals of the indole ring showed significant changes compared with those in *N*-(2-methylaminobenzoyl)tryptamine (**16**), particularly the downfield shift of C-2 (*δ*_C_ 179.8, Δ*δ* + 56.3 ppm) and the upfield shift of C-7 (*δ*_C_ 44.8, Δ*δ*—68.7 ppm). Furthermore, the HMBC correlations from H-7 to C-2/C-5/C-13 were observed, indicating that the double bond placed at C-2 and C-7 in *N*-(2-methylaminobenzoyl)tryptamine (**16**) was replaced by a carbonyl group in compound **2** (Fig. 2). Although compounds *N*-(2-methylaminobenzoyl)tryptamine (**16**) and **2** had similar structures, the ^1^H NMR spectrum of *N*-(2-methylaminobenzoyl)tryptamine (**16**) did not exhibit broadening signals. The broadening signals of the ^1^H NMR spectrum of compound **2** were attributed to the existence of an amide moiety, and the 4-NH as an active hydrogen capable could form the intermolecular hydrogen bond with the carbonyl group at C-2. In addition, the specific rotation value of compound **2** was close to zero. Combined with ECD experiments showing no obvious Cotton effect (Figure S17) and the presence of only one chiral center at C-7, compound **2** was determined to be a racemic mixture, and named wuchuyuamine B.

Wuchuyuamine C (**3)** was obtained as a white amorphous powder. Based on the HRESIMS ion peak at *m/z* 358.1529 [M + Na]^+^ (calcd. for C_20_H_21_N_3_O_2_Na, 358.1526), its molecular formula was determined as C_20_H_21_N_3_O_2_. Analysis of the ^1^H and ^13^C NMR data (600 and 150 MHz, respectively, in CD_3_OD; Table 1) for compound **3** revealed a similar structure to 10-methoxygoshuyuamide-II [19]. The molecular weight of compound **3** was 14 less than that of 10-methoxygoshuyuamide-II, and this difference was due to the presence of a carbonyl substitution at C-3 in 10-methoxygoshuyuamide-II. The HMBC correlations from H_2_-3 to C-5/C-15/C-21/*N*–CH₃, and from H_2_-5 to C-3/C-7/C-21 confirmed the above speculation (Fig. 2), and therefore the structure of compound **3** was determined as wuchuyuamine C.

Wuchuyuamine D (**4**) was isolated as a white amorphous powder. The molecular formula C_19_H_19_N_3_O_2_ was determined by HRESIMS ion peak at *m/z* 322.1554 [M + H]^+^ (calcd. for C_19_H_20_N_3_O_2_, 322.1550). The ^1^H NMR (500 MHz, CD_3_OD) and ^13^C NMR (125 MHz, CD_3_OD) data (Table 1) displayed signals corresponding to indole and quinazoline rings, indicating that it was an indolequinazoline alkaloid with a similar skeleton to evodiamine (**7**) [15]. Comparison with the ^13^C NMR data of evodiamine (**7**) suggested that C-5 (*δ*_C_ 63.3) and C-6 (*δ*_C_ 28.4) in compound **4** were shifted downfield and the chemical shifts were close to C-5 (*δ*_C_ 63.4) and C-6 (*δ*_C_ 28.5) in compound **1**. The HMBC spectrum of compound **4** also lacked the correlations from H-5 to C-3/C-21 (Fig. 2). Similar to compound **1**, the C ring of compound **4** was broken with the bond disruption of the C-5 and *N*-4, and a hydroxyl group was linked to C-5. The specific rotation was close to zero, and the experimental ECD spectra showed no obvious Cotton effect (Figure S34). Therefore, compound **4** was determined to be a racemic mixture, the structure was depicted as shown in Fig. 1.

Wuchuyuamine E (**23**) was obtained as a yellow oil. Its molecular formula was determined to be C_25_H_31_NO_3_ based on the HRESIMS ion peak at *m/z* 394.2380 [M + H]^+^ (calcd. for C_25_H_32_NO_3_, 394.2377). The UV spectrum showed absorption maxima at 215, 239, 322, and 335 nm, which were characteristic of the *N*-methyl-4(1*H*)-quinolone skeleton, indicating that compound **23** was a quinolone alkaloid. The ^1^H and ^13^C NMR data (500 and 125 MHz, respectively, in CD_3_OD; Table 2) also displayed characteristic signals of the *N*-methyl-4(1*H*)-quinolone skeleton [*δ*_H_ 6.31 (1H, s, H-3), 8.32 (1H, dd, *J* = 8.1, 1.5 Hz, H-5), 7.47 (1H, ddd, *J* = 7.9, 6.8, 1.1 Hz, H-6), 7.80 (1H, ddd, *J* = 8.7, 6.8, 1.6 Hz, H-7), 7.86 (1H, dd, *J* = 8.8, 1.3 Hz, H-8), 3.89 (3H, s,* N*-CH_3_);* δ*_C_ 158.8 (C-2), 111.1 (C-3), 179.5 (C-4), 126.9 (C-4a), 126.6 (C-5), 125.1 (C-6), 133.9 (C-7), 117.9 (C-8), 143.5 (C-8a), 35.5 (*N*-CH_3_)]. Combined with the ^1^H-^1^H COSY and HSQC, the signals of the aliphatic side chain were assigned as [*δ*_C_ 35.6, 29.5, 30.2, 29.9, 28.2, 133.1, 126.0, 36.0,72.5, 148.0, 128.9, 145.3, 131.1, 201.4, 27.1 (C1′-C15′)]. In the ^1^H-^1^H COSY spectrum, the correlations of H-7′/H-8′/H-9′/H-10′, combined with HMBC correlations from H-10′ to C-8′/C-12′, from H-11′ to C-9′/C-13′ (Fig. 2), along with their chemical shifts, indicated the hydroxyl group located at the position of C-9′. Additionally, HMBC correlations from H-3 to C-1′ and from H-2′ to C-2 confirmed that the aliphatic carbon chain was connected to C-2. Based on the coupling constants (^3^*J*_H-6′,H-7′_ = 18.1 Hz, ^3^*J*_H-10′,H-11′_ = 15.3 Hz, ^3^*J*_H-12′,H-13′_ = 15.6 Hz), the configurations of the couple of olefinic carbons between C-6′ and C-7′, between C-10′ and C-11′, between C-12′ and C-13′ in compound **23** were elucidated as *E*-form. Compound **23** was further confirmed to be a racemate because it showed no Cotton effect and its optical rotation value was close to zero (Figure S43). From above mentioned results, the structure of **23** was elucidated as wuchuyuamine E.

**Table 2:** ^1^H NMR (500 MHz) and ^13^C NMR (125 MHz) spectroscopic data for **23** in CD_3_OD (*δ* in ppm, *J* in Hz)

No	*δ*_H_	*δ*_C_	No	*δ*_H_	*δ*_C_
*N*-CH_3_	3.89 (3H, s)	35.5	4′	1.48 (2H, m)	29.9
2		158.8	5′	2.10 (2H, m)	28.2
3	6.31 (1H, s)	111.1	6′	5.51 (1H, dt, 18.2, 7.3)	133.1
4		179.5	7′	5.43 (1H, dt, 18.1, 7.2)	126.0
4a		126.9	8′	2.32 (2H, m)	36.0
5	8.32 (1H, dd, 8.1, 1.5)	126.6	9′	4.21 (1H, dt, 6.6, 5.3)	72.5
6	7.47 (1H, ddd, 7.9, 6.8, 1.1)	125.1	10′	6.27 (1H, dd, 15.3, 5.7)	148.0
7	7.80 (1H, ddd, 8.7, 6.8, 1.6)	133.9	11′	6.41 (1H, dd, 15.4, 11.0)	128.9
8	7.86 (1H, dd, 8.8, 1.3)	117.9	12′	7.25 (1H, dd, 15.7, 10.8)	145.3
8a		143.5	13′	6.14 (1H, d, 15.6)	131.1
1′	2.89 (2H, t, 7.8)	35.6	14′		201.4
2′	1.73 (2H, m)	29.5	15′	2.25 (3H, s)	27.1
3′	1.48 (2H, m)	30.2			

Wuchuyuamine F (**29**) was a white amorphous powder. The HRESIMS ion peak at *m/z* 320.1362 [M + Na]^+^ (calcd. for C_17_H_19_N_3_O_2_Na, 320.1369) and 1D NMR data (^1^H NMR, 600 MHz; ^13^C NMR, 150 MHz), recorded in CD_3_OD (Table 3), led to the determination of the molecular formula as C_17_H_19_N_3_O_2_. ^13^C NMR data of **29** indicated a similar structure to wuchuyuamide III (**31**), except for the absence of a carbonyl signal. In the HMBC spectrum (Fig. 2), compound **29** lacked the key correlations from H-9′ to C-2 and from *N*–CH₃ to C-2. Therefore, the absent carbonyl group was located at the position of C-2 and the structure of compound **29** was constructed (Fig. 1).

**Table 3:** ^1^H NMR and ^13^C NMR spectroscopic data for **29** and **30** (*δ* in ppm, *J* in Hz)

No	**29**^a^		**30**^b^	
	*δ*_H_	*δ*_C_	*δ*_H_	*δ*_C_
4		172.3		170.0
5	7.39 (1H, dd, 7.8, 1.6)	129.1	7.30 (1H, m)	127.3
6	6.58 (1H, m)	115.9	6.58 (1H, dd, 7.6, 7.6)	114.6
7	7.29 (1H, ddd, 8.6, 7.1, 1.6)	133.6	7.30 (1H, m)	133.2
8	6.68 (1H, d, 8.4)	111.9	6.66 (1H, d, 8.3)	111.3
9		151.4		150.8
10		117.4		114.8
*N*-CH_3_	2.83 (3H, s)	29.9	2.85 (3H, d, 5.0)	29.8
1′		118.6		121.6
2′		152.7		140.1
3′	6.74 (1H, d, 8.4)	118.3	8.76 (1H, d, 8.5)	121.9
4′	7.23 (1H, ddd, 8.5, 6.9, 1.5)	135.5	7.58 (1H, dd, 8.0, 7.9)	135.7
5′	6.58 (1H, m)	116.2	7.17 (1H, dd, 7.8, 7.7)	123.4
6′	7.80 (1H, dd, 8.3, 1.5)	132.4	7.94 (1H, d, 8.0)	131.1
7′		202.5		204.0
8′	3.27 (2H, t, 6.8)	39.5	3.39 (2H, t, 5.7)	39.8
9′	3.69 (2H, t, 6.8)	36.8	3.82 (2H, dt, 5.9, 5.8)	34.7
CHO			8.52 (1H, s)	160.0
*N*H-CHO			11.5 (1H, s)	

^a^600 MHz in CD_3_OD; 150 MHz in CD_3_OD

^b^600 MHz in CDCl_3_; 150 MHz in CDCl_3_

Wuchuyuamine G (**30**) was obtained as a white amorphous powder. Its molecular formula, C_18_H_19_N_3_O_3_, was deduced from the HRESIMS ion peak at *m/z* 326.1499 [M + H]^+^ (calcd. for C_18_H_20_N_3_O_3_, 326.1499). Since **30** exhibited similar UV, IR, and NMR spectral characteristics to **29**, it was supposed that compound **30** had the same skeleton as **29**. Analysis of the ^1^H and ^13^C NMR spectra of **30**, recorded in CDCl_3_ at 600 and 150 MHz (Table 3), showed an additional aldehyde signal (*δ*_H_ 8.52;* δ*_C_ 160.0) compared with **29**. Combined with the HMBC correlation from aldehyde proton (*δ*_H_ 8.52) to C-2′ (Fig. 2), it could be concluded that the aldehyde group and C-2′ were connected through the amino group. The structure of compound **30** was determined as depicted in Fig. 1.

### Antifungal activity in vitro

The antifungal activities of compounds **2**, **4**, **12**–**14**, **16**, **18**, **19**, **24**–**26**, **31,** and **33**, available in sufficient amounts, were evaluated against three phytopathogenic fungi. The results are summarized in Table S1. Among the tested compounds, **16** and **24** exhibited pronounced inhibitory activity against *Alternaria solani*, with inhibition rates approaching 80% and 90% at 100 μg/mL, respectively. To further assess their antifungal potency, the IC_50_ values of **16** and **24** against *A. solani* were determined (Table 4). Both compounds **16** and **24** demonstrated dose-dependent inhibition, although their activities were slightly lower than that of the commercial fungicide hymexazol. These results suggest that the structural frameworks of **16** and **24** may confer selective antifungal activity toward *A. solani.*

**Table 4:** Antifungal activities with IC_50_ of compounds **13**, **16**, **24**, **25**

Test fungi	Compounds	IC_50_ (*μ*g/mL)	95% CI	R^2^
*A. solani*	**16**	33.39	29.58–38.26	0.9964
	**24**	36.02	32.26–40.79	0.9977
	Hymexazol^a^	14.90	11.23–19.92	0.9872
*F. graminearum*	**13**	> 100	–	–
	**25**	13.15	8.51–21.36	0.9710
	Hymexazol^a^	30.86	23.61–43.67	0.9829

^a^Hymexazol was used as the positive control

To further investigate the broad-spectrum antifungal potential of the major alkaloids in *T. ruticarpum*, compounds **13**, **19** and **25**, identified as the high-content constituents in the fruit extract, were selected for testing against sixteen phytopathogenic fungi, including *Botrytis *sp., *Ceratocystis fimbriata*, *Colletotrichum capsici f. nicotianae*, *Colletotrichum *sp., *Curvularia lunata*, *Exserohilum turcicum*, *Fusarium graminearum*, *Fusarium moniliforme*, *Fusarium oxysporum*, *Gerlachia nivalis*, *Neofusicoccum *sp., *Pestalotiopsis maculans*, *Pestalotiopsis *sp., *Phytophthora capsici*, *Rhizoctonia solani*, *Verticillium dahliae*, with hymexazol serving as the positive control (Table S2). Among the three alkaloids, compound **25** presented the most pronounced antifungal activity, with inhibition rates varying between 7.2% and 83.5%. Notably, compound **25** showed stronger inhibitory activity against *F. graminearum* than hymexazol, with an IC_50_ value of 13.15 μg/mL (Table 4), highlighting its potential as a promising lead compound for the development of plant-derived fungicides. Additionally, compound **13** showed moderate inhibition activity, with a maximum inhibition rate of 72.9% at 100 μg/mL, depending on the pathogen tested. Unfortunately, compound **19** exhibited negligible inhibitory activity across all tested fungi, indicating limited antifungal potential.

### Observation of morphology and ultrastructure in *F. graminearum*

**Fig. 3:**
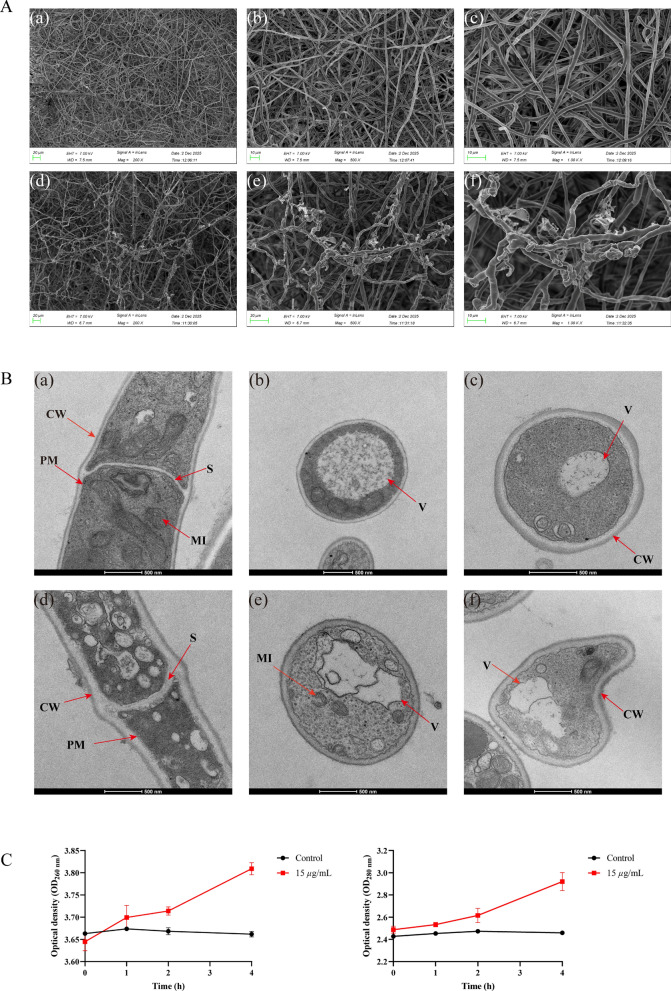
**A** SEM images [200 × (a, d), 500 × (b, e) and 1000 × (c, f) magnification] of *F. graminearum* hyphae treated with DMSO (a-c) and **25** at 15 µg/mL (d-f). **B** TEM images [22,000 × (a, d-f), 17,500 × (b) and 28,000 × (c) magnification] of *F. graminearum* hyphae treated with DMSO (a-c) and **25** at 15 µg/mL (d-f). Scale bars are provided in each image in (**A**) and (**B**). CW, cell wall; PM, plasma membrane; S, septum; V, vacuole; MI, mitochondrion. **C** Leakage of nucleic acids (OD_260 nm_) and proteins (OD_280 nm_)

Scanning Electron Microscopy (SEM) was employed to observe the morphological effects of **25** on *F. graminearum* hyphae, in which the mycelia of *F. graminearum* treated by DMSO were used as blank controls. As shown in Fig. 3A, the hyphae on the PSA medium containing DMSO were slender, uniform, and smooth. In contrast, **25** (15 μg/mL) caused disordered growth and distortion, with a roughened surface, localized collapse and hyphal breakage. These results indicated that **25** caused severe damage to the hyphae of *F. graminearum*.

Transmission Electron Microscopy (TEM) observations further confirmed ultrastructural disruption (Fig. 3B). Control hyphae displayed intact cell walls with well-defined plasma membranes, preserved mitochondria, and normal vacuoles. After treatment with **25** (15 μg/mL), the cell wall contour became deformed and the plasma membrane was indistinct, accompanied by pronounced cytoplasmic vacuolization and markedly wrinkled vacuolar membranes. In addition, some mitochondria showed disorganization and compromised membrane integrity. These observations suggest that **25** disrupted the cellular ultrastructure of *F. graminearum.*

### Effect of 25 on the cytoplasmic content leakage of *F. graminearum*

Membrane damage was evaluated by measuring leakage of intracellular components. To assess the effect of **25**, nucleic acid (OD_260 nm_) and protein (OD_280 nm_) leakage were quantified. As shown in Fig. 3C, OD_260 nm_ and OD_280 nm_ values in the DMSO control remained relatively stable over time. In contrast, treatment with **25** (15 µg/mL) led to a time-dependent increase in OD_260 nm_ and OD_280 nm_ within 4 h. This demonstrates that **25** enhanced cell membrane permeability, resulting in membrane rupture and subsequent leakage of intracellular contents.

## Conclusion

In summary, thirty-six alkaloids, including seven novel compounds, wuchuyuamines A-G (**1**–**4**, **23**, **29**, and **30**), were isolated and identified from the fruits of *T. ruticarpum*. The antifungal activities of representative alkaloids were systematically evaluated against a range of phytopathogenic fungi. Compounds **16** and **24** exhibited moderate inhibitory activity against *A. solani*, with IC_50_ values of 33.39 and 36.02 μg/mL, respectively. Notably, compound **25** demonstrated marked broad-spectrum antifungal efficacy, particularly against *F. graminearum*, where its inhibitory potency (IC_50_ = 13.15 μg/mL) exceeded that of the commercial fungicide hymexazol. The SEM and TEM analyses indicated that **25** disrupted the surface morphology and cellular ultrastructure of *F. graminearum* hyphae. Compound **25** induced leakage of intracellular contents, resulting in metabolic dysregulation and ultimately leading to cell death. These findings not only expand the chemical diversity of alkaloids from *T. ruticarpum* but also highlight the antifungal potential of quinolone alkaloids as promising lead structures for the development of novel, efficient, and environmentally friendly fungicides.

## Experimental

### General experimental procedures

HRESIMS data were acquired on an Agilent 1260 UPLC/6540 Q-TOF MS spectrometer (Agilent, USA). Optical rotations were measured in MeOH using an Autopol VI spectropolarimeter (Rudolph Research Analytical). ECD spectra were recorded on a Chirascan instrument (Applied Photophysics Ltd., Leatherhead, UK). 1D and 2D NMR spectra were obtained on Bruker 500 or 600 MHz spectrometer with chemical shifts (*δ*) given in ppm with reference to the solvent and coupling constants (*J*) in Hz (Bruker, Germany). UV spectra were obtained on a Shimadzu UV-2401PC spectrophotometer (Shimadzu, Tokyo, Japan). IR spectra were recorded on a Bruker Vertex 70 IR spectrometer (Bruker, Germany). Column chromatography was performed using Sephadex LH-20, RP-18, and silica gel. An X-Bridge C_18_ column (Waters, 10 mm × 250 mm, 5 μm) and a YMC-Triart C_18_ column (YMC, 10 mm × 250 mm, 5 μm) were used for HPLC analysis.

### Plant material

The fruits of *T. ruticarpum* were purchased from Chongqing Zhongmiao Pharmaceutical Co., Ltd. (Chongqing, China) in September 2023 (Batch No. 230201). The plant material was identified by Dr. Chun-Lei Xiang, Kunming Institute of Botany, Chinese Academy of Sciences. A voucher specimen (NO. KIBH20230915) was deposited at the State Key Laboratory of Phytochemistry and Natural Medicines, Kunming Institute of Botany, Chinese Academy of Sciences.

### Fungal materials

Plant pathogenic fungi *A. solani*, *Bipolaris sorokiniana* and *Fusarium odoratissimum* were obtained from the Institute of Microbiology, Chinese Academy of Sciences. The remaining fungal strains were supplied by the State Key Laboratory for Conservation and Utilization of Bio-Resources in Yunnan, China.

### Extraction and isolation

The separation and isolation procedures for compounds **1**–**36** and spectroscopic data of **1**–**4**, **23**, **29**, and **30** are provided in the Supporting Information (SI).

### Spectroscopic data

(**1**): yellow amorphous powder; UV (MeOH) *λ*_max_ (log *ε*) 207 (4.07), 219 (4.13), 257 (3.58), 278 (3.35), 343 (3.20) nm; IR (KBr) *ν*_max_ 3433, 1632, 1520, 1384, 1275, 747 cm^−1^; ^1^H and ^13^C NMR data (CD_3_OD, 500 and 125 MHz), see Table 1; HRESIMS *m/z* 346.1533 [M + Na]^+^ (calcd. for C_19_H_21_N_3_O_2_Na, 346.1526).

(**2**): white amorphous powder; UV (MeOH) *λ*_max_ (log *ε*) 204 (4.53), 241 (4.05), 307 (3.48) nm; IR (KBr) *ν*_max_ 3390, 2920, 1617, 1582, 1412, 1062, 750 cm^−1^; ^1^H and ^13^C NMR data ((CD_3_)_2_CO, 600 and 150 MHz), see Table 1; HRESIMS *m/z* 310.1547 [M + H]^+^ (calcd. for C_18_H_20_N_3_O_2_, 310.1550).

(**3**): white amorphous powder; UV (MeOH) *λ*_max_ (log *ε*) 207 (4.08), 221 (4.15), 297 (3.20), 340 (2.97), 349 (2.94) nm; IR (KBr) *ν*_max_ 3434, 2924, 1638, 1384, 1215, 759 cm^−1^; ^1^H and ^13^C NMR data (CD_3_OD, 600 and 150 MHz), see Table 1; HRESIMS *m/z* 358.1529 [M + Na]^+^ (calcd. for C_20_H_21_N_3_O_2_Na, 358.1526).

(**4**): white amorphous powder; UV (MeOH) *λ*_max_ (log *ε*) 227 (4.83), 268 (4.15), 347 (3.48) nm; IR (KBr) *ν*_max_ 3390, 3281, 1657, 1488, 1041, 749 cm^−1^; ^1^H and ^13^C NMR data (CD_3_OD, 500 and 125 MHz), see Table 1; HRESIMS *m/z* 322.1554 [M + H]^+^ (calcd. for C_19_H_20_N_3_O_2_, 322.1550).

(**23**): yellow oil; UV (MeOH) *λ*_max_ (log *ε*) 215 (3.27), 239 (3.28), 274 (2.88), 322 (2.91), 335 (2.91) nm; IR (KBr) *ν*_max_ 3433, 2922, 1620, 1598, 1557, 1384, 1268, 1179, 763 cm^−1^; ^1^H and ^13^C NMR data (CD_3_OD, 500 and 125 MHz), see Table 2; HRESIMS *m/z* 394.2380 [M + H]^+^ (calcd. for C_25_H_32_NO_3_, 394.2377).

(**29**): white amorphous powder; UV (MeOH) *λ*_max_ (log *ε*) 208 (3.82), 219 (3.85), 257 (3.45), 355 (3.20) nm; IR (KBr) *ν*_max_ 3448, 2920, 1637, 1520, 1277, 1210, 1166, 750 cm^−1^; ^1^H and ^13^C NMR data (CD_3_OD, 600 and 150 MHz), see Table 3; HRESIMS *m/z* 320.1362 [M + Na]^+^ (calcd. for C_17_H_19_N_3_O_2_Na, 320.1369).

(**30**): white amorphous powder; UV (MeOH) *λ*_max_ (log *ε*) 207 (4.07), 219 (4.14), 225 (4.14), 257 (3.87), 329 (3.48) nm; IR (KBr) *ν*_max_ 3405, 2922, 1641, 1520, 1277, 1200, 1172, 752 cm^−1^; ^1^H and ^13^C NMR data (CDCl_3_, 600 and 150 MHz), see Table 3; HRESIMS *m/z* 326.1499 [M + H]^+^ (calcd. for C_18_H_20_N_3_O_3_, 326.1499).

### Antifungal activity assay in vitro

Compounds **2**, **4**, **12–14**, **16**, **18**, **19**, **24–26**, **31** and **33** were evaluated for their antifungal activities against *A. solani*, *B. sorokiniana* and *F. odoratissimum* at 100 μg/mL following the mycelium growth rate method [38]. For compounds exhibiting appreciable inhibition, dose–response assays were conducted at serial concentrations, and IC_50_ values were calculated from the fitted inhibition curves using the same method. Hymexazol was used as a positive control. All results were expressed as the mean ± SD. Broad-spectrum antifungal activities of compounds **13**, **19** and **25** were further evaluated against sixteen phytopathogenic fungi using the same mycelium growth rate method.

### SEM and TEM observations

*Fusarium graminearum* was treated with compound **25** at a concentration of 15 μg/mL, while the solvent served as the control. Subsequently, SEM and TEM were used to observe alterations in mycelial morphology and intracellular ultrastructure, respectively [39].

### Effect of 25 on the cytoplasmic content leakage of *F. graminearum*

The mycelium (400 mg) was suspended in 40 mL of sterile water containing **25** (15 µg/mL) and incubated at 26 ℃ for 0, 1, 2, or 4 h, with DMSO as the solvent control. After incubation, the supernatant absorbance at 260 and 280 nm was measured using a SpectraMax iD3 microplate reader (Molecular Devices, USA) to assess nucleic acid and protein leakage, respectively [40].

### Statistical analysis

All experiments were conducted with three independent biological replicates, with consistent results among replicates. The half-maximal inhibitory concentration (IC_50_) values were calculated by nonlinear regression (log[inhibitor] vs. normalized response, Variable slope) using GraphPad Prism (GraphPad Software, USA). Absorbance at 260 and 280 nm (OD_260 nm_ and OD_280 nm_) was analyzed and plotted in GraphPad Prism.

## Supplementary Information

Supplementary Material 1. Extraction and isolation; Antifungal activities; 1D and 2D NMR, HRESIMS of compounds **1**–**4**, **23**, **29**, and **30**.

## Data Availability

The data underlying this study are available in the published article and its Supporting Information.
