# Supplementary material for: Wuchuyuamines A-G, seven new alkaloids isolated from the fruits of Tetradium ruticarpum and their antifungal activities
Source: Nat Prod Bioprospect. 2026 Aug 10;16(1):81. doi: 10.1007/s13659-026-00610-6 (PMC13457749; doi:10.1007/s13659-026-00610-6)

Supplementary Information

Wuchuyuamines A-G, seven new alkaloids isolated from the fruits of *Tetradium ruticarpum* and their antifungal activities

Meng-Ting Chen[1,3], Jian Zhang[1], Qian Zhao[1,3], Su-Min Zhang[1], Yin-Ling Wei[1,3], Ying Zhang[1], Xiao-Jiang Hao[1,2,3,4*]

[1] *State Key Laboratory of Phytochemistry and Natural Medicines, Kunming Institute of Botany, Chinese Academy of Sciences, Kunming, 650201, China*

[2] *Yunnan Characteristic Plant Extraction Laboratory, Kunming, 650106, China*

[3] *University of Chinese Academy of Sciences, Beijing, 100049, China*

[4] *Research Unit of Chemical Biology of Natural Anti-Virus Products, Chinese Academy of Medical Sciences, Beijing, 100730, China*

*Correspondence:

Xiao-Jiang Hao

haoxj@mail.kib.ac.cn

Tel: +86-871-65223070; Fax: +86-871-65223263

Contents

1 Extraction and Isolation

2 Antifungal activities Assay

Table S1. Antifungal activities of alkaloids against three pathogenic fungi 9

Table S2. Broad-spectrum antifungal activities of compounds **13**, **19** and **25**. 10

3 Spectra for compounds 1-4, 23, 29, and 30

Figure S1. HRESIMS spectrum of compound **1** 11

Figure S2. UV spectrum of compound **1** 12

Figure S3. IR spectrum of compound **1** 12

Figure S4. ^{1}H NMR spectrum of compound **1** (500 MHz, CD_3OD) 13

Figure S5. ^{13}C NMR spectrum of compound **1** (125 MHz, CD_3OD) 13

Figure S6. ^{1}H-^{1}H COSY spectrum of compound **1** 14

Figure S7. HSQC spectrum of compound **1** 14

Figure S8. HMBC spectrum of compound **1** 15

Figure S9. HRESIMS spectrum of compound **2** 16

Figure S10. UV spectrum of compound **2** 17

Figure S11. IR spectrum of compound **2** 17

Figure S12.1. ^{1}H NMR spectrum of compound **2** before derivatization (600 MHz, CD_3OD)..18

Figure S12.2. ^{1}H NMR spectrum of compound **2** (600 MHz, $(CD_3)_2CO$)..................18

Figure S13.1 ^{13}C NMR spectrum of compound **2** before derivatization (150 MHz, CD_3OD)..19

Figure S13.2 ^{13}C NMR spectrum of compound **2** (150 MHz, $(CD_3)_2CO$) 19

Figure S14. ^{1}H-^{1}H COSY spectrum of compound **2**..20

Figure S15. HSQC spectrum of compound **2** 20

Figure S16. HMBC spectrum of compound **2** 21

Figure S17. CD spectrum of compound **2**..21

Figure S18. HRESIMS spectrum of compound **3** 22
Figure S19. UV spectrum of compound **3** 23
Figure S20. IR spectrum of compound **3** 23
Figure S21. ^{1}H NMR spectrum of compound **3** (600 MHz, CD_3OD) 24
Figure S22. ^{13}C NMR spectrum of compound **3** (150 MHz, CD_3OD) 24
Figure S23. ^{1}H-^{1}H COSY spectrum of compound **3** 25
Figure S24. HSQC spectrum of compound **3** 25
Figure S25. HMBC spectrum of compound **3** 26
Figure S26. HRESIMS spectrum of compound **4** 27
Figure S27. UV spectrum of compound **4** 28
Figure S28. IR spectrum of compound **4** 28
Figure S29. ^{1}H NMR spectrum of compound **4** (500 MHz, CD_3OD) 29
Figure S30. ^{13}C NMR spectrum of compound **4** (125 MHz, CD_3OD) 29
Figure S31. ^{1}H-^{1}H COSY spectrum of compound **4** 30
Figure S32. HSQC spectrum of compound **4** 30
Figure S33. HMBC spectrum of compound **4** 31
Figure S34. CD spectrum of compound **4** 31
Figure S35. HRESIMS spectrum of compound **23** 32
Figure S36. UV spectrum of compound **23** 33
Figure S37. IR spectrum of compound **23** 33
Figure S38. ^{1}H NMR spectrum of compound **23** (500 MHz, CD_3OD) 34
Figure S39. ^{13}C NMR spectrum of compound **23** (125 MHz, CD_3OD) 34
Figure S40. ^{1}H-^{1}H COSY spectrum of compound **23** 35
Figure S41. HSQC spectrum of compound **23** 35
Figure S42. HMBC spectrum of compound **23** 36
Figure S43. CD spectrum of compound **23** 36

Figure S44. HRESIMS spectrum of compound **29** 37

Figure S45. UV spectrum of compound **29** 38

Figure S46. IR spectrum of compound **29** 38

Figure S47. ^{1}H NMR spectrum of compound **29** (600 MHz, CD_3OD) 39

Figure S48. ^{13}C NMR spectrum of compound **29** (150 MHz, CD_3OD) 39

Figure S49. ^{1}H-^{1}H COSY spectrum of compound **29** 40

Figure S50. HSQC spectrum of compound **29** 40

Figure S51. HMBC spectrum of compound **29** 41

Figure S52. HRESIMS spectrum of compound **30** 42

Figure S53. UV spectrum of compound **30** 43

Figure S54. IR spectrum of compound **30** 43

Figure S55. ^{1}H NMR spectrum of compound **30** (600 MHz, $CDCl_3$) 44

Figure S56. ^{13}C NMR spectrum of compound **30** (150 MHz, $CDCl_3$) 44

Figure S57. ^{1}H-^{1}H COSY spectrum of compound **30** 45

Figure S58. HSQC spectrum of compound **30** 45

Figure S59. HMBC spectrum of compound **30** 46

1 Extraction and Isolation

The air-dried, powdered fruits of *Tetradium ruticarpum* (30 kg) were extracted under reflux with 95% ethanol three times (100 L for 4 h, followed by 80 L for 3 h, and another 80 L for 3 h). After removing the solvent under reduced pressure, the ethanolic extract was suspended in H_2O and acidified to pH 2-3 with 5% HCl, and partitioned three times with petroleum ether to remove non-alkaloid constituents. The acid solution was subsequently adjusted to pH 9-10 with 10% NaOH and partitioned three times with CH_2Cl_2. The CH_2Cl_2 extracts were concentrated under reduced pressure to obtain 550 g of total alkaloids. The total alkaloids were chromatographed on silica gel with a gradient elution of PE/EA (50:1 to 1:1, *v*/*v*), followed by CH_2Cl_2/MeOH (10:1 to 1:1, *v*/*v*) to provide five fractions (Fr.A-Fr.E). Fr.B was recrystallized from MeOH to give compound **5** (5.7 g).

Fr.C (155 g) was chromatographed on RP-18 medium-pressure column chromatography (20-45 μm) with MeOH/H_2O (30:70 to 100:0, *v*/*v*) to provide seven subfractions (Fr.C1-Fr.C7). Among them, Fr.C2 (12.78 g) was further chromatographed on silica gel column chromatography with a gradient elution of CH_2Cl_2/MeOH (100:0-0:100, *v*/*v*), which afforded five fractions designated as Fr.C2.1-Fr.C2.5. Fr.C2.4 (5.28 g) was chromatographed by RP-18 column chromatography into three subfractions (Fr.C2.4.1-Fr.C2.4.3). Fr.C2.4.2 (2.11 g) was subsequently separated by Sephadex LH-20 to yield compound **13** (341 mg). HPLC purification afforded compounds **22** (3.8 mg) and **34** (1.1 mg). Fr.C2.4.3 (690 mg) was also chromatographed on Sephadex LH-20 in methanol and was further purified by HPLC (MeCN/H_2O, 45:55, *v*/*v*) to obtain compound **2** (53 mg). Fr.C2.5 (862 mg) was separated by RP-18 column chromatography into four fractions (Fr.C2.5.1-Fr.C2.5.4). Fr.C2.5.2 (505 mg) was further purified by silica gel column chromatography using a gradient elution of CH_2Cl_2/MeOH (100:1-1:1, *v*/*v*) to yield compound **19** (420 mg). Fr.C2.5.3 (71 mg) was further purified by HPLC (MeCN/H_2O, 25:75, *v*/*v*) to obtain compound **10** (7.2 mg).

Fr.C3 (4.56 g) was chromatographed on silica gel column chromatography with a gradient elution of PE/EA (5:1 to 0:1, *v*/*v*), affording five fractions (Fr.C3.1-Fr.C3.5).

Fr.C3.3 (1.27 g) was further chromatographed on Sephadex LH-20 in methanol, followed by HPLC ($MeCN/H_2O$, 30:70, *v*/*v*) to yield compounds **8** (8.5 mg), **30** (2.3 mg), and **33** (24 mg). Fr.C3.4 (1.60 g) was chromatographed on Sephadex LH-20 in methanol followed by HPLC ($MeCN/H_2O$, 40:60, *v*/*v*) to yield compound **15** (6.5 mg). Fr.C3.5 (822 mg) was separated by Sephadex LH-20 in methanol and was subsequently purified by HPLC ($MeCN/H_2O$, 35:65, *v*/*v*) to yield compounds **4** (18 mg), **9** (5.7 mg), and **32** (4.1 mg).

Fr.C4 (621 mg) was chromatographed on Sephadex LH-20 in CH_2Cl_2/MeOH (1:1, *v*/*v*) and further purified by HPLC to get compounds **1** (13 mg), **3** (1.8 mg), **29** (2.1 mg), and **31** (30 mg).

Fr.C5 (50 g) was separated by RP-18 column chromatography ($MeOH/H_2O$, 30:70 to 100:0, *v*/*v*) to yield seven subfractions (Fr.C5.1-Fr.C5.7). Fr.C5.3 was recrystallized from MeOH to afford compound **7** (3.0 g). Fr.C5.5 (2.16 g) was separated by silica gel column chromatography using a gradient elution of CH_2Cl_2/MeOH (100:1 to 1:1, *v*/*v*) into eight subfractions (Fr.C5.5.1-Fr.C5.5.8). Fr.C5.5.2 (264 mg) was purified by HPLC ($MeCN/H_2O$, 55:45, *v*/*v*) to obtain compound **21** (9.3 mg). Fr.C5.5.3 (280 mg) was purified by HPLC ($MeCN/H_2O$, 40:60, *v*/*v*) to obtain compound **16** (48 mg). Fr.C5.5.4 (715 mg) underwent a three-step purification process: first, RP-18 column chromatography ($MeOH/H_2O$, 30:70 to 100:0, *v*/*v*); second, Sephadex LH-20 (CH_2Cl_2/MeOH, 1:1, *v*/*v*); and finally, HPLC purification ($MeCN/H_2O$, 60:40, *v*/*v*). This sequence afforded compounds **14** (43 mg) and **24** (16.7 mg). Fr.C5.7 (5.17 g) was separated by silica gel column chromatography with a gradient elution of PE/EA (10:1 to 1:1, *v*/*v*) to provide five subfractions (Fr.C5.7.1-Fr.C5.7.5). Fr.C5.7.5 (3.56 g) was chromatographed on Sephadex LH-20 in methanol to get compound **25** (2.7 g). A subfraction (Fr.C5.7.5.7) was further separated by HPLC ($MeOH/H_2O$, 50:50, *v*/*v*) to obtain compound **12** (52 mg). Fr.C5.7.5.9 was purified by HPLC ($MeCN/H_2O$, 48:52, *v*/*v*) to obtain compound **6** (3.0 mg).

Fr.C6 (1.51 g) was chromatographed on silica gel column chromatography using a PE/EA gradient (10:1 to 1:1, *v*/*v*), yielding four subfractions (Fr.C6.1–Fr.C6.4).

Fr.C6.3 (1.30 g) was further purified through RP-18 column chromatography and HPLC, affording compounds **17** (1.0 mg), **23** (5.1 mg), **26** (21 mg), **27** (3.1 mg), and **28** (1.0 mg).

Fr.D (120 g) was chromatographed on silica gel with a gradient elution of PE/EA (20:1 to 2:1, *v*/*v*), followed by CH_2Cl_2/MeOH (10:1 to 1:1, *v*/*v*) to afford seven fractions (Fr.D1–Fr.D7). Fr.D2 (6.37 g) was chromatographed by RP-18 column chromatography and further purified by HPLC to provide compound **36** (5.2 mg). Fr.D5 (10.68 g) was separated by RP-18 column chromatography (MeOH/H_2O, 30:70 to 100:0, *v*/*v*) to yield ten subfractions (Fr.D5.1-Fr.D5.10). Fr.D5.4 (1.63 g) was chromatographed on silica gel column chromatography with a gradient elution of CH_2Cl_2/MeOH (30:1 to 5:1, *v*/*v*) and further separated by HPLC to obtain compound **20** (3.8 mg). Fr.D5.6 (1.29 g) was chromatographed on silica gel column chromatography with a gradient elution CH_2Cl_2/MeOH (30:1 to 10:1, *v*/*v*) and further separated by HPLC to afford compound **11** (3.9 mg). Fr.D7 (702 mg) was further purified by Sephadex LH-20 in methanol, followed by HPLC purification to yield compounds **18** (25 mg) and **35** (3.7 mg).

2 Antifungal activities Assay

Antifungal Activity Assay in Vitro

The test fungi were cultured on potato sucrose agar medium (PSA: potato 200 g, sucrose 20 g, agar 15 g, and distilled water 1000 mL) and maintained at 28 °C throughout the experiments.

The compounds **2**, **4**, **12-14**, **16**, **18**, **19**, **24-26**, **31**, and **33** were evaluated for their inhibitory effects on mycelial growth on PSA medium against three phytopathogenic fungi (*Alternaria solani*, *Bipolaris sorokiniana* and *Fusarium odoratissimum*) at a concentration of 100 μg/mL. Additionally, compounds **13**, **19** and **25** were further assessed for their broad-spectrum antifungal activities against sixteen plant pathogenic fungi at a concentration of 100 μg/mL. Each compound was dissolved in dimethyl sulfoxide (DMSO) and then was added to PSA medium pre-prepared and sterilized to obtain the test concentration (100 μg/mL). The blank control consisted of PSA supplemented with 0.5% (*v*/*v*) DMSO, while hymexazol was used as the positive control.

Mycelial disks (6 mm in diameter) of phytopathogenic fungi were inoculated onto PSA plates and incubated at 28°C. Each treatment was performed in triplicate, and colony diameters (mm) were measured using the cross-bracketing method. For compounds requiring IC_{50} determination, additional series of concentrations (50, 25, 10, 5 and 1 μg/mL) were prepared. Growth inhibition rates were calculated using the following formula once the mycelia of the blank control had grown to the edge of the Petri dish.

mycelial growth inhibition (%) = $[(C-T)/(C-6\text{ mm})]\times 100$

where *C* and *T* represent the diameters of fungal growth on untreated PSA and treated PSA, respectively.

Table S1. Antifungal activities of alkaloids against three pathogenic fungi

compound	inhibition rate ± SD (%, 100 μg/mL)[a]		
	A. solani	*B. sorokiniana*	*F. odoratissimum*
**2**	10.4 ± 3.0	25.2 ± 3.9	28.2 ± 1.8
**4**	20.4 ± 2.8	31.6 ± 4.3	11.0 ± 2.9
**12**	58.5 ± 2.1	55.6 ± 2.2	63.6 ± 0.1
**13**	26.0 ± 2.8	40.5 ± 2.1	67.6 ± 3.1
**14**	8.5 ± 0.3	34.4 ± 0.5	8.8 ± 2.9
**16**	79.6 ± 1.5	57.4 ± 2.6	57.6 ± 1.8
**18**	24.6 ± 0.6	36.5 ± 3.9	NA
**19**	14.7 ± 3.4	31.8 ± 3.1	NA
**24**	89.6 ± 3.0	58.8 ± 1.5	31.2 ± 3.0
**25**	43.6 ± 3.9	48.6 ± 1.6	NA
**26**	52.2 ± 3.8	56.8 ± 3.3	12.5 ± 2.3
**31**	15.9 ± 1.0	36.5 ± 3.1	11.0 ± 2.9
**33**	30.9 ± 2.4	63.5 ± 4.1	77.9 ± 3.4
Hymexazol[b]	100.0 ± 0.0	100.0 ± 0.0	93.7 ± 0.2
NA: no activity			

[a] Average of three replicates.

[b] Hymexazol was used as the positive control.

Table S2. Broad-spectrum antifungal activities of compounds **13**, **19** and **25**

phytopathogenic fungi	inhibition rate ± SD (%, 100 μg/mL)[a]			
	**13**	**19**	**25**	Hymexazol[b]
Botrytis sp.	49.3 ± 1.7	3.6 ± 2.8	7.2 ± 3.2	50.0 ± 2.8
C. fimbriata	72.9 ± 1.1	15.3 ± 2.6	16.0 ± 1.5	31.1 ± 2.6
C. capsici f. nicotianae	20.5 ± 3.0	NA	30.8 ± 1.2	25.5 ± 2.4
Colletotrichum sp.	61.7 ± 2.9	NA	17.0 ± 1.6	82.0 ± 2.2
C. lunata	45.5 ± 2.8	16.2 ± 4.4	77.1 ± 3.4	77.9 ± 3.2
E. turcicum	68.7 ± 1.3	4.1 ± 1.9	63.4 ± 4.0	11.9 ± 1.3
F. graminearum	50.9 ± 3.1	12.4 ± 3.0	81.9 ± 1.5	83.3 ± 2.2
F. moniliforme	36.0 ± 1.2	NA	28.0 ± 2.9	72.2 ± 3.6
F. oxysporum	29.3 ± 1.7	6.1 ± 1.1	20.0 ± 2.8	53.3 ± 3.1
G. nivalis	52.5 ± 2.8	6.3 ± 4.6	52.6 ± 3.1	62.3 ± 3.8
Neofusicoccum sp.	8.4 ± 2.2	NA	49.1 ± 5.4	13.3 ± 1.2
P. maculans	34.9 ± 2.4	NA	53.8 ± 3.4	83.3 ± 1.6
Pestalotiopsis sp.	47.3 ± 1.4	10.3 ± 2.2	83.5 ± 2.0	100.0 ± 0.0
P. capsici	NA	NA	10.9 ± 1.4	81.6 ± 2.6
R. solani	24.8 ± 2.3	NA	29.0 ± 3.3	41.4 ± 1.6
V. dahliae	25.0 ± 1.3	NA	13.2 ± 2.1	19.2 ± 2.8
NA: no activity				

[a] Average of three replicates.

[b] Hymexazol was used as the positive control.

3 Spectra for compounds 1-4, 23, 29, and 30

Figure S1. HRESIMS spectrum of compound **1**

Qualitative Analysis Report

**Data Filename**	HCMT-59a.d	**Sample Name**	HCMT-59a
**Sample Type**	Sample	**Position**	P1-F9
**Instrument Name**	Instrument 1	**User Name**	
**Acq Method**	s.m	**Acquired Time**	8/12/2025 11:46:41 AM
**IRM Calibration Status**	Success	**DA Method**	PCDL.m
**Comment**			

**Sample Group**		**Info.**
**Acquisition SW Version**	6200 series TOF/6500 series Q-TOF B.05.01 (B5125.2)	

User Spectra

Fragmentor Voltage	Collision Energy	Ionization Mode
135	0	ESI

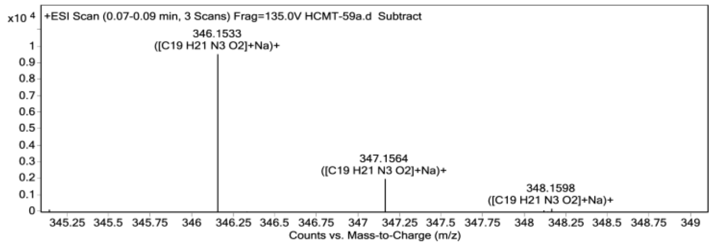

Peak List

m/z	z	Abund	Formula	Ion
174.0917	1	3829.04		
324.1714	1	6129.6		
346.1533	1	9554.71	C19 H21 N3 O2	(M+Na)+
347.1564	1	2032.53	C19 H21 N3 O2	(M+Na)+
362.1307	1	2529.2		
669.3168	1	7440.34		
670.3192	1	3328.93		
685.3088	1	1586.61		
856.4072	1	2264.43		
857.4098	1	1281.36		

Formula Calculator Element Limits

Element	Min	Max
C	3	50
H	0	100
O	0	20
N	0	5

Formula Calculator Results

Formula	CalculatedMass	CalculatedMz	Mz	Diff. (mDa)	Diff. (ppm)	DBE
C19 H21 N3 O2	323.1634	346.1526	346.1533	-0.70	-2.02	11.0000

--- End Of Report ---

Agilent Technologies Page 1 of 1 Printed at: 1:30 PM on: 8/12/2025

Figure S2. UV spectrum of compound **1**

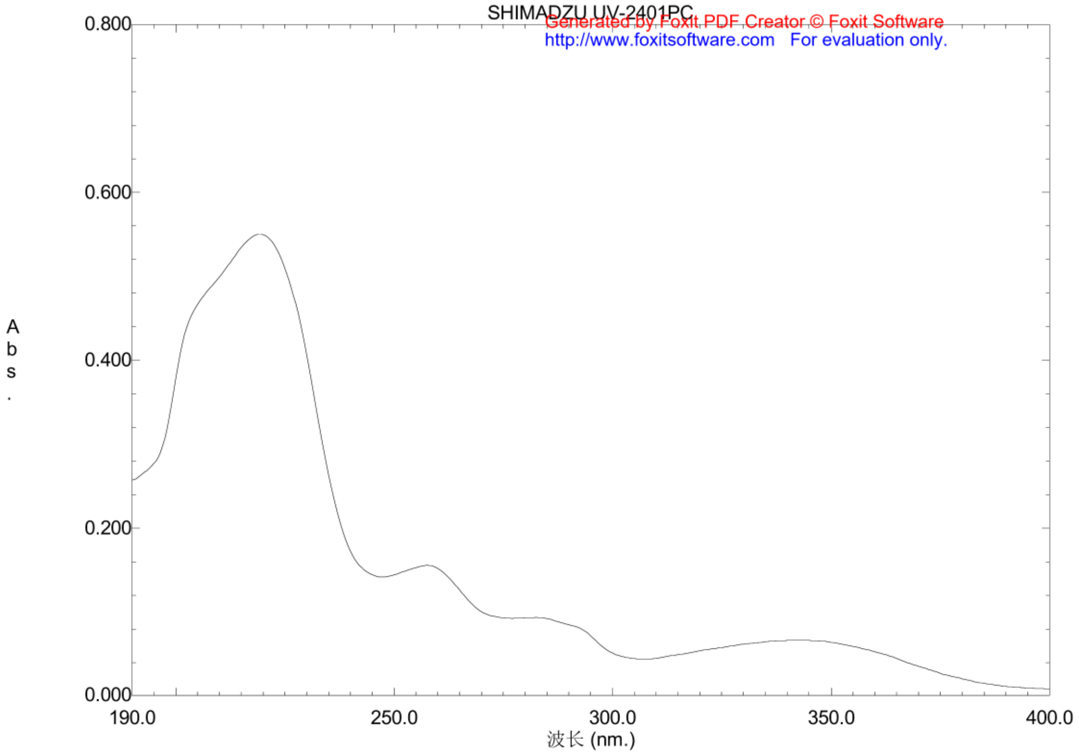

Figure S3. IR spectrum of compound **1**

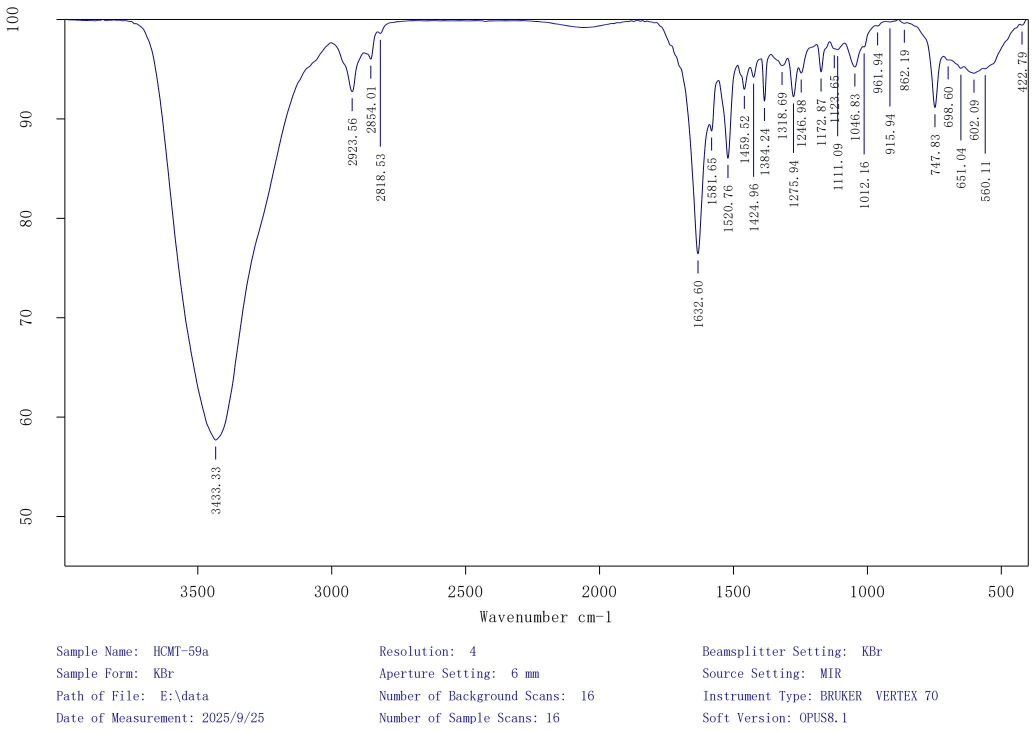

Figure S4. ^{1}H NMR spectrum of compound **1** (500 MHz, CD_3OD)

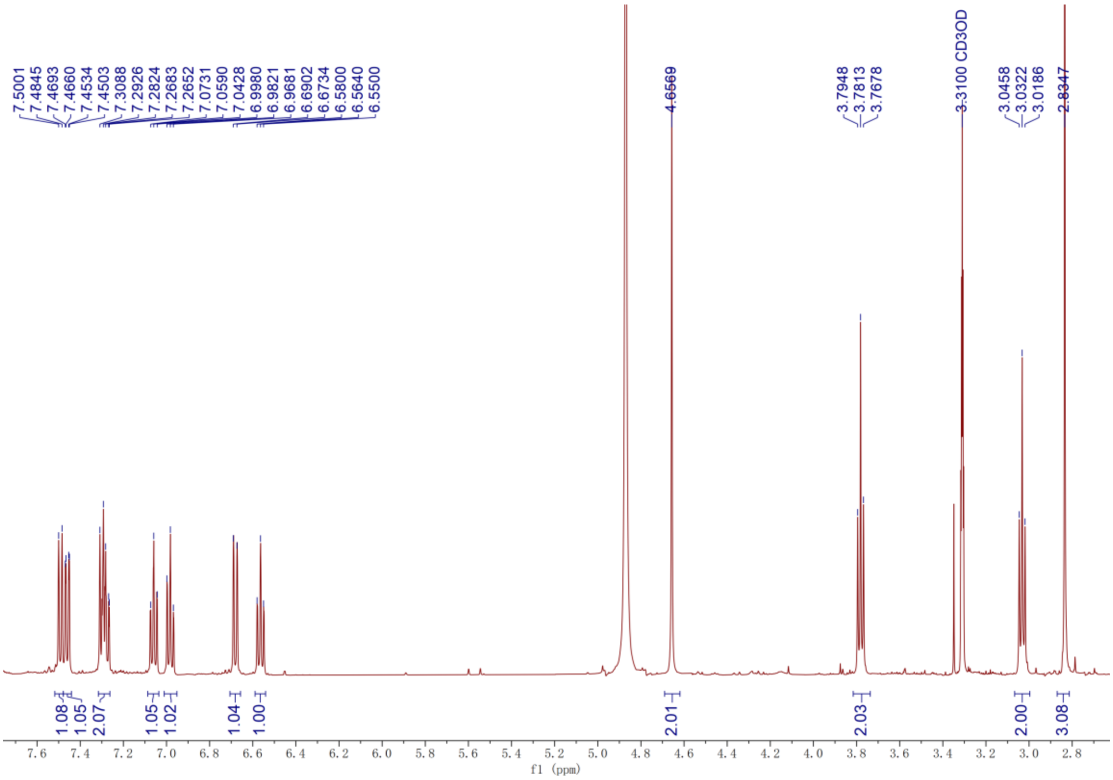

Figure S5. ^{13}C NMR spectrum of compound **1** (125 MHz, CD_3OD)

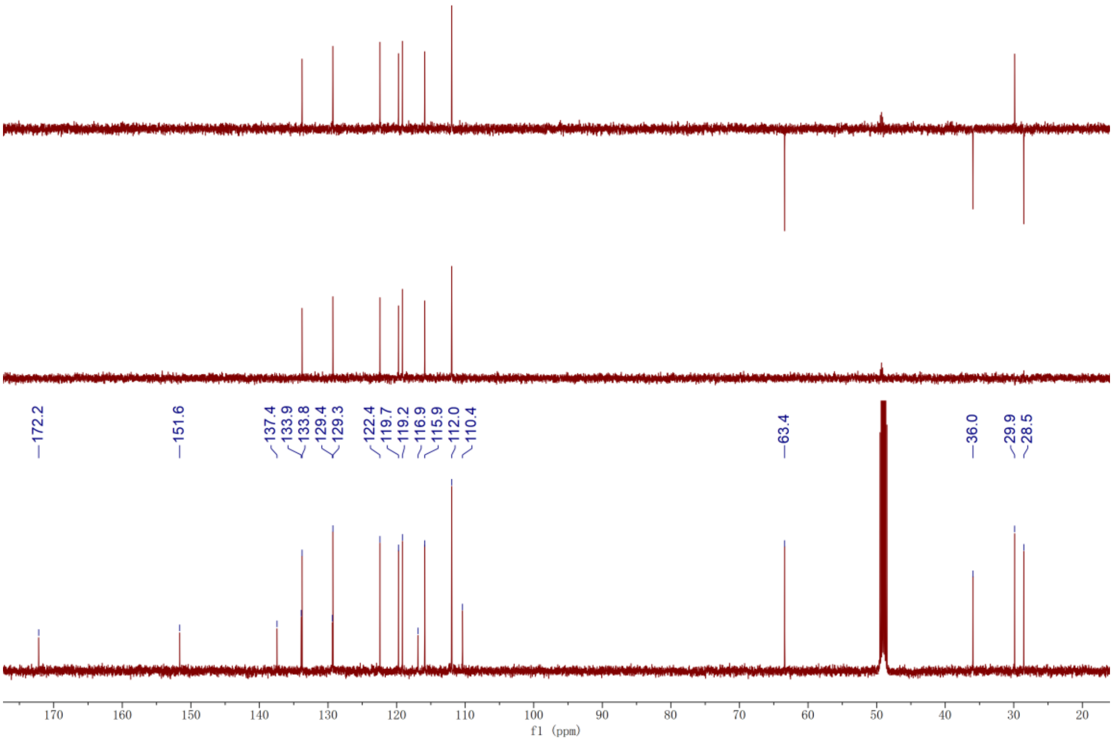

Figure S6. ^{1}H-^{1}H COSY spectrum of compound **1**

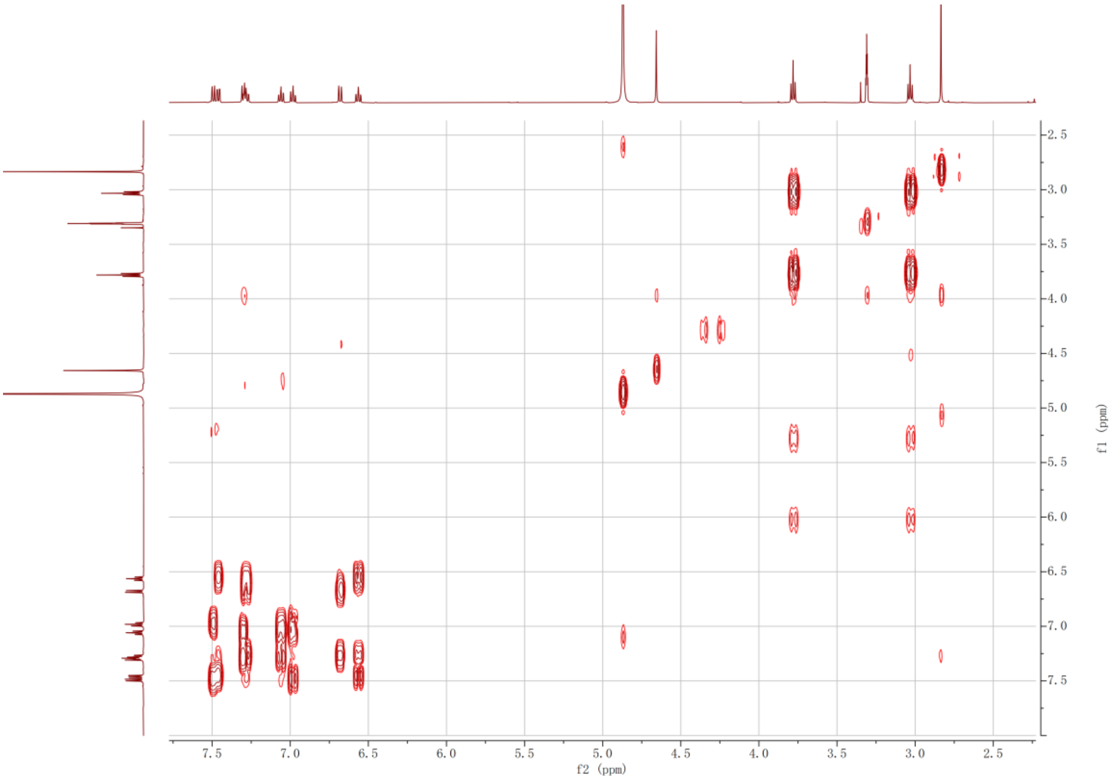

Figure S7. HSQC spectrum of compound **1**

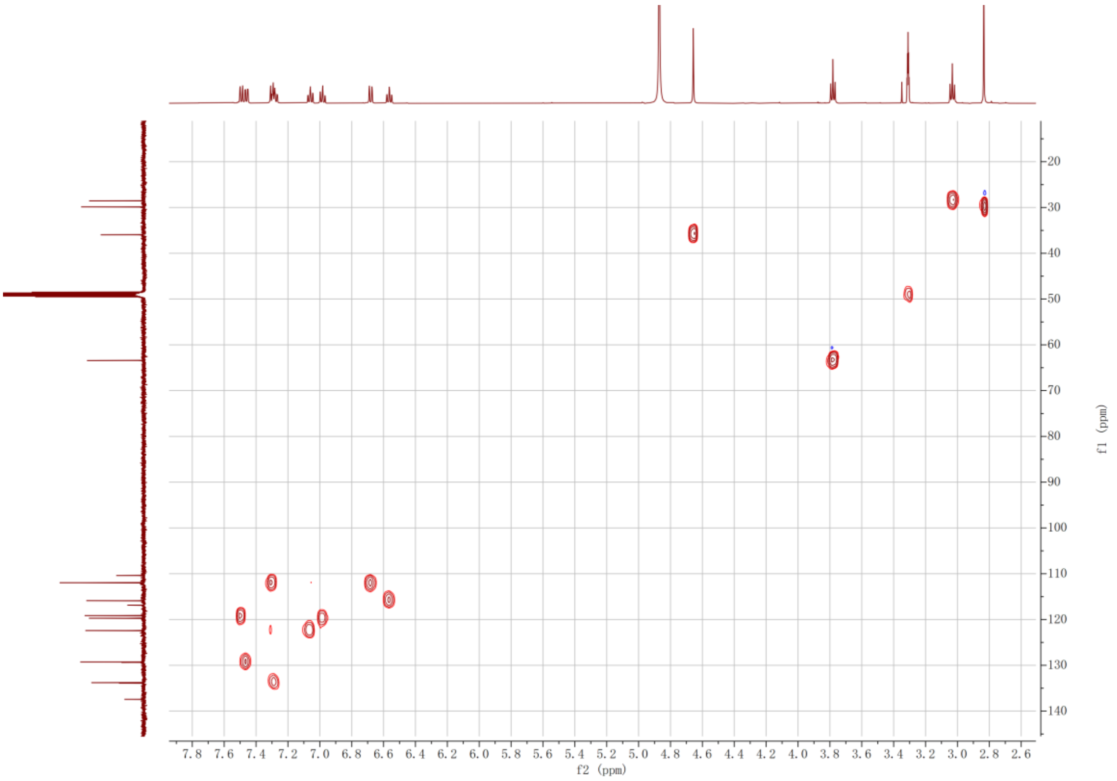

Figure S8. HMBC spectrum of compound **1**

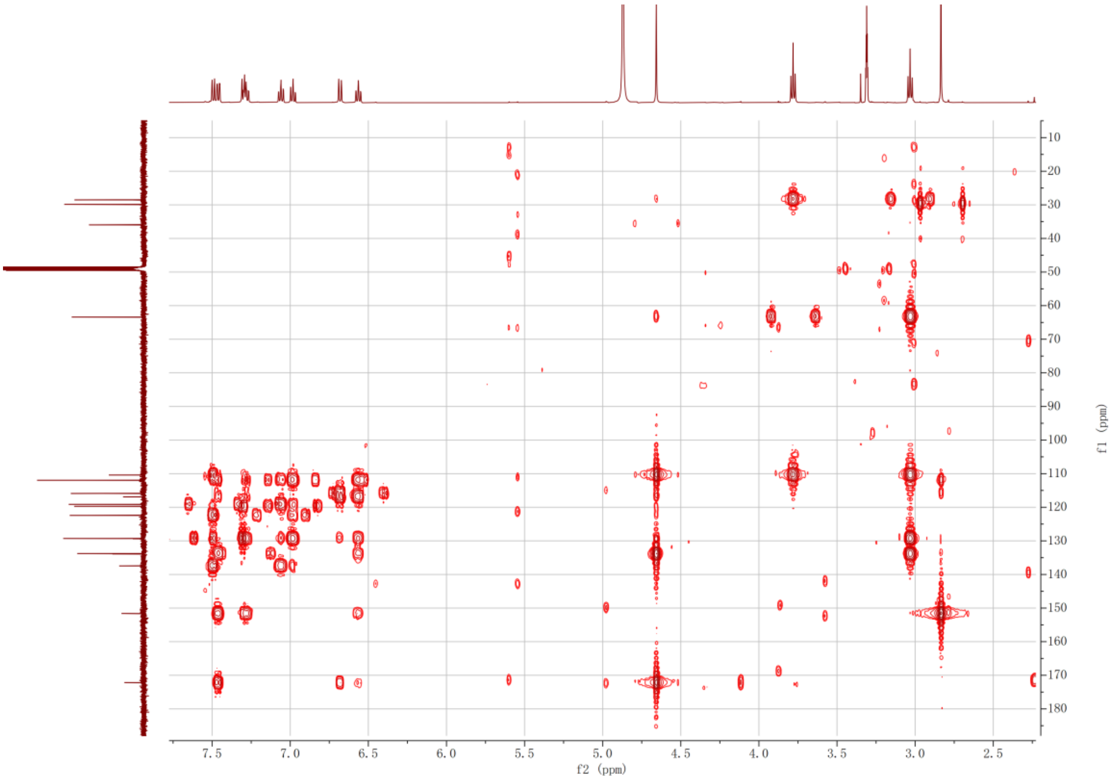

Figure S9. HRESIMS spectrum of compound **2**

Qualitative Analysis Report

**Data Filename**	HCMT-32a1.d	**Sample Name**	HCMT-32a1
**Sample Type**	Sample	**Position**	P1-A3
**Instrument Name**	Instrument 1	**User Name**	
**Acq Method**	s.m	**Acquired Time**	9/24/2024 4:46:40 PM
**IRM Calibration Status**	Success	**DA Method**	PCDL.m
**Comment**			

Sample Group **Info.**
Acquisition SW 6200 series TOF/6500 series
Version Q-TOF B.05.01 (B5125.2)

User Spectra

Fragmentor Voltage	Collision Energy	Ionization Mode
135	0	ESI

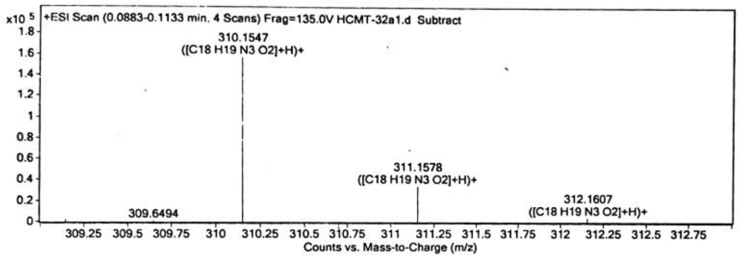

Peak List

m/z	z	Abund	Formula	Ion
122.0813	1	12601.58		
134.0598	1	19801.07		
298.1547	1	2853.72		
309.1465	1	3024.05		
310.1547	1	156982.7	C18 H19 N3 O2	(M+H)+
311.1578	1	34122	C18 H19 N3 O2	(M+H)+
312.1607	1	3809.89	C18 H19 N3 O2	(M+H)+
326.1491	1	7465.75		
332.1363	1	4261.78		
340.1641	1	3508.41		

Formula Calculator Element Limits

Element	Min	Max
C	3	60
H	0	200
O	0	20
N	0	8

Formula Calculator Results

Formula	CalculatedMass	CalculatedMz	Mz	Diff. (mDa)	Diff. (ppm)	DBE
C18 H19 N3 O2	309.1477	310.1550	310.1547	0.30	0.97	11.0000

--- End Of Report ---

Agilent Technologies Page 1 of 1 Printed at: 4:48 PM on: 9/24/2024

Figure S10. UV spectrum of compound **2**

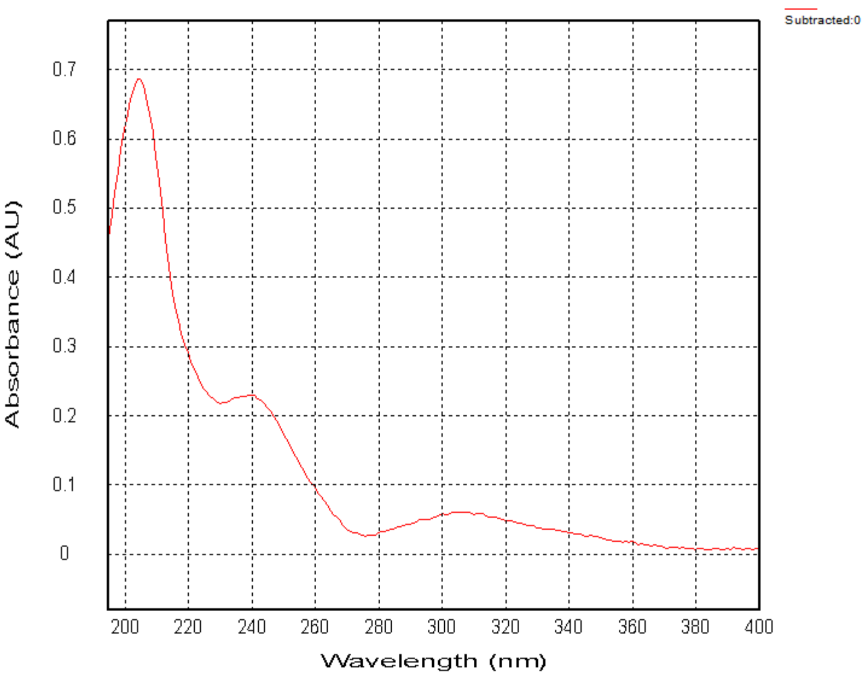

Figure S11. IR spectrum of compound **2**

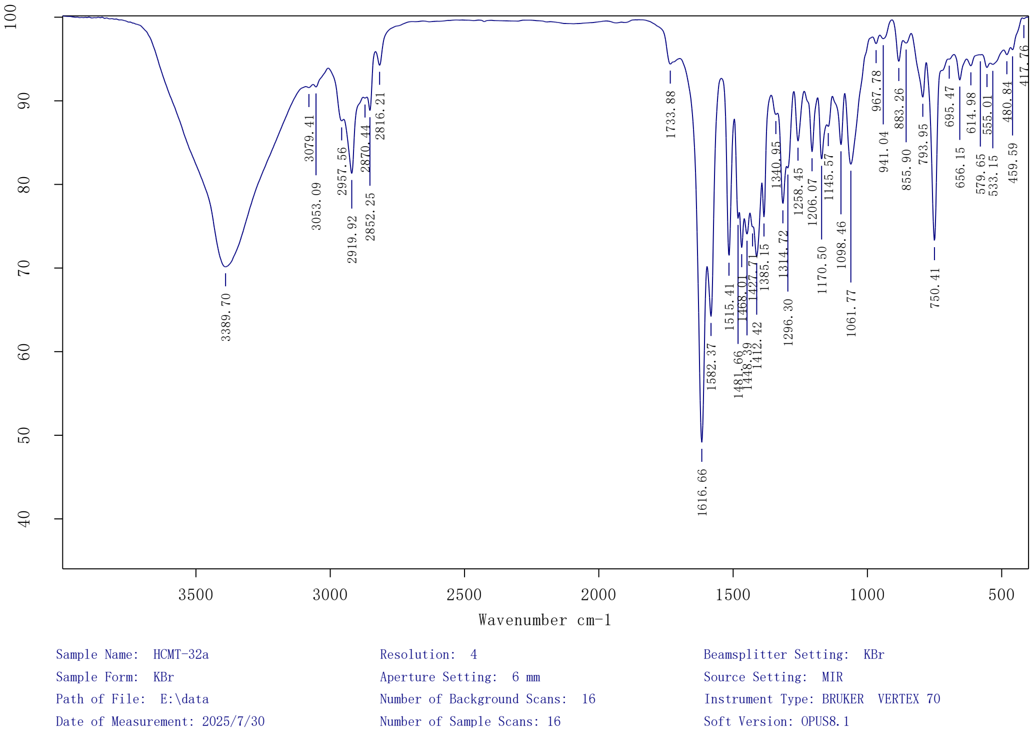

Figure S12.1. ^{1}H NMR spectrum of compound **2** before derivatization (600 MHz, CD_3OD)

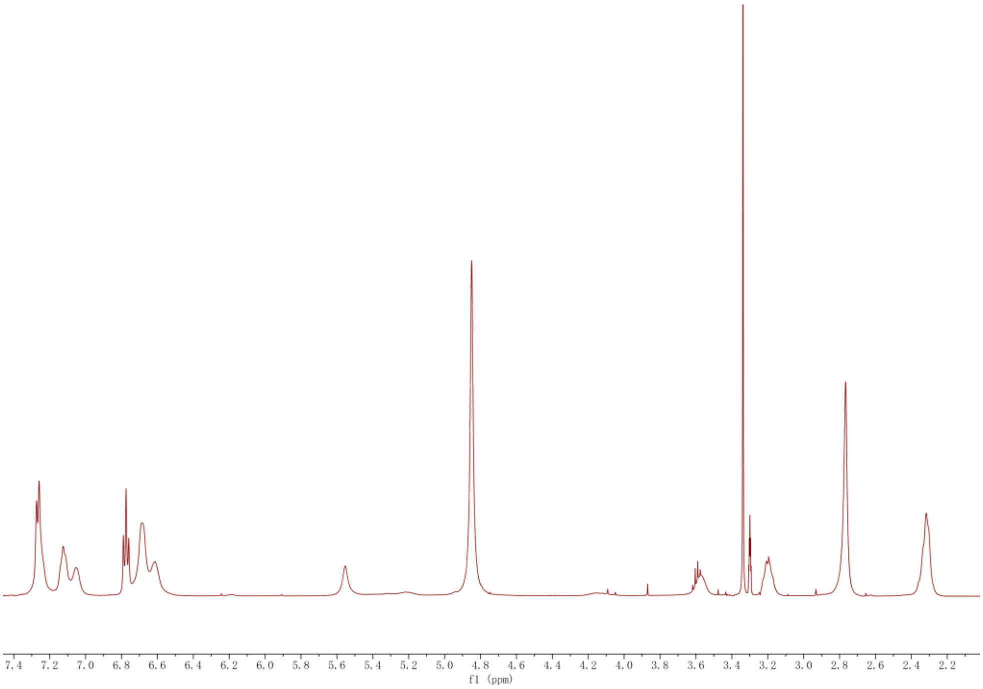

Figure S12.2. ^{1}H NMR spectrum of compound **2** (600 MHz, $(CD_3)_2CO$)

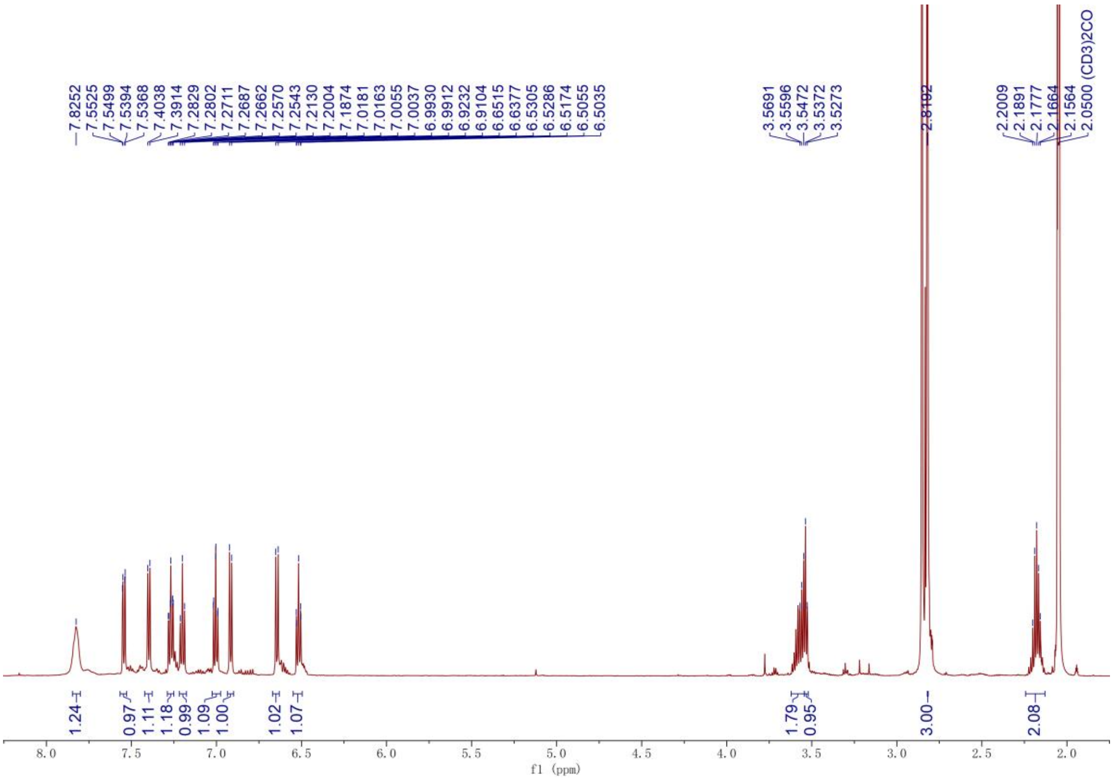

Figure S13.1 ^{13}C NMR spectrum of compound **2** before derivatization (150 MHz, CD_3OD)

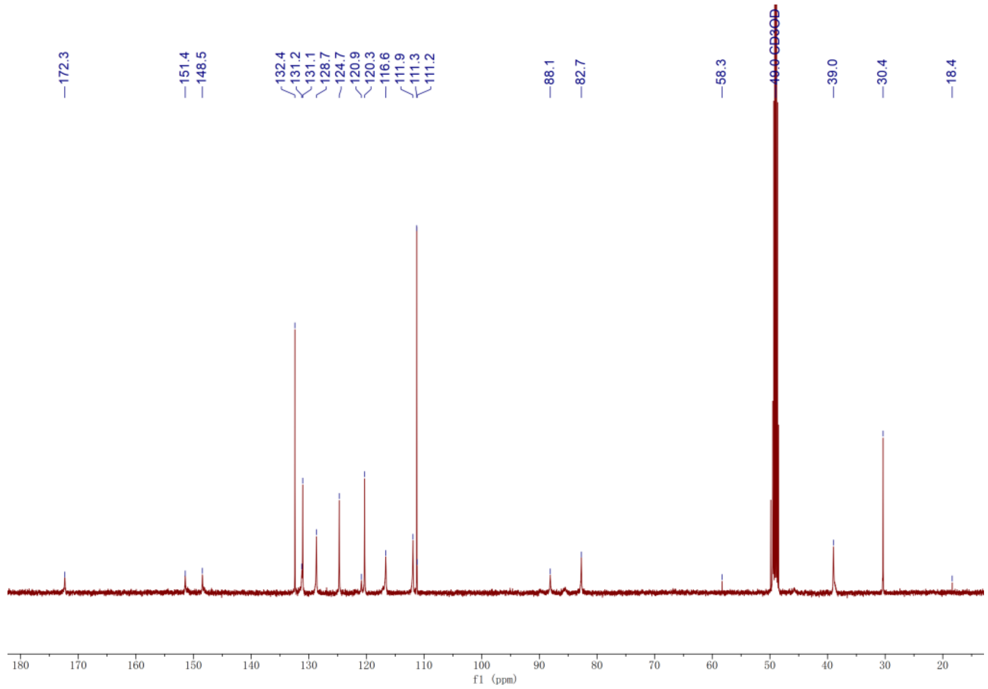

Figure S13.2 ^{13}C NMR spectrum of compound **2** (150 MHz, $(CD_3)_2CO$)

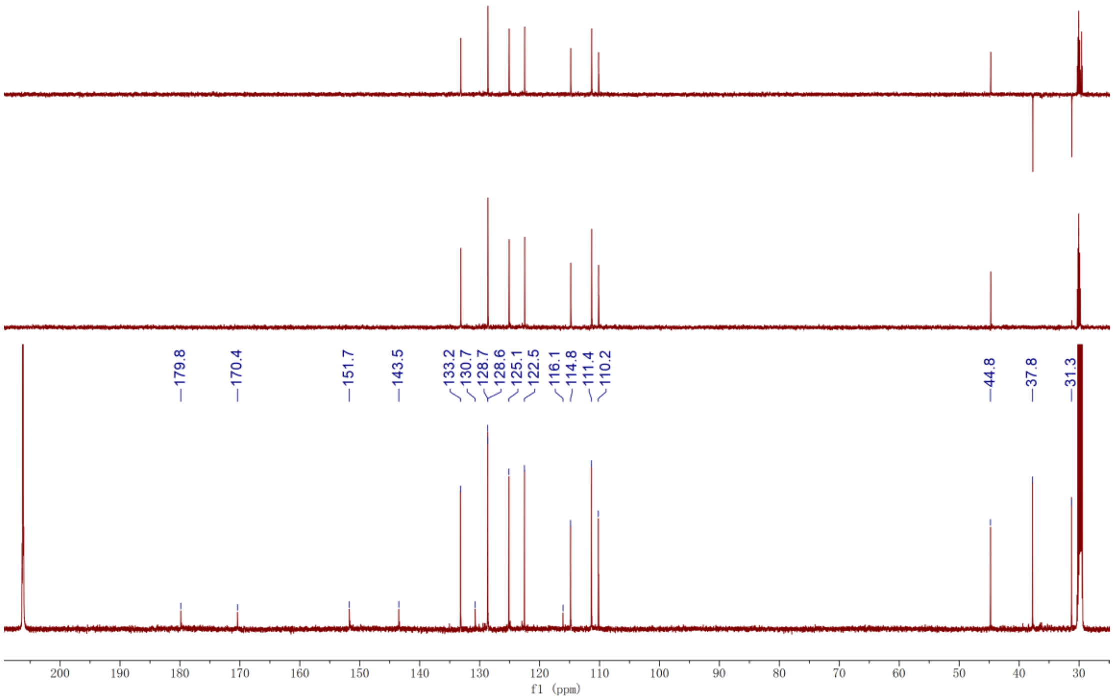

Figure S14. ^{1}H-^{1}H COSY spectrum of compound **2**

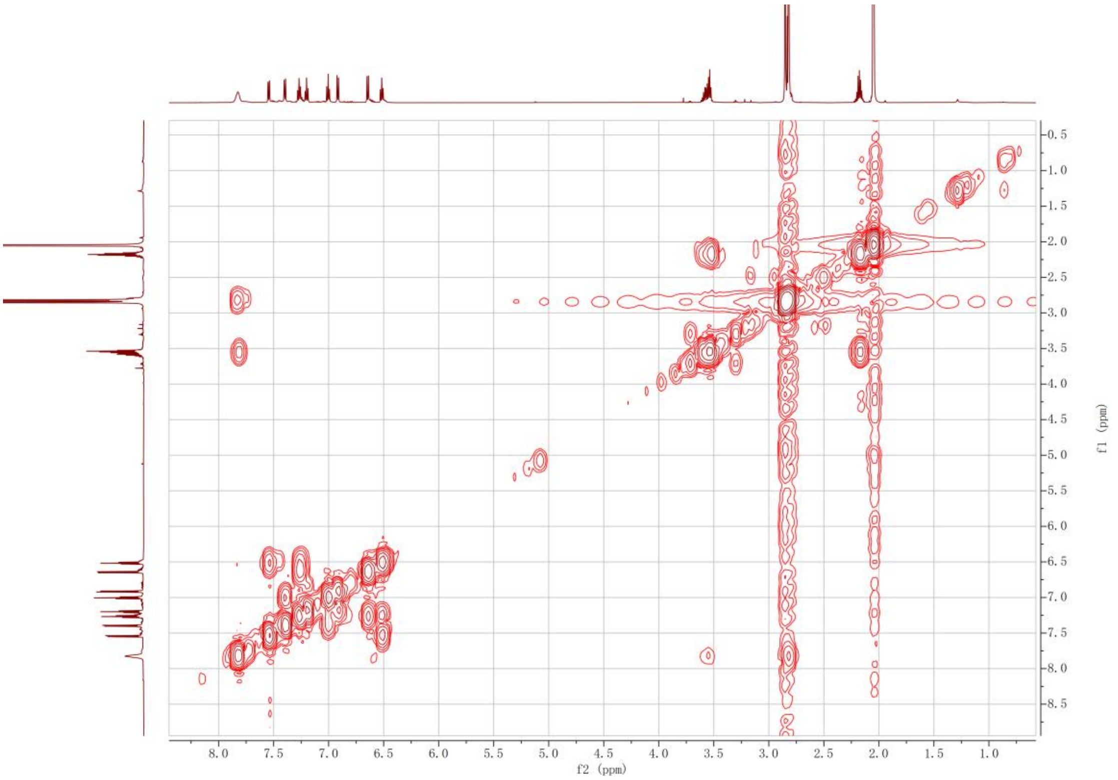

Figure S15. HSQC spectrum of compound **2**

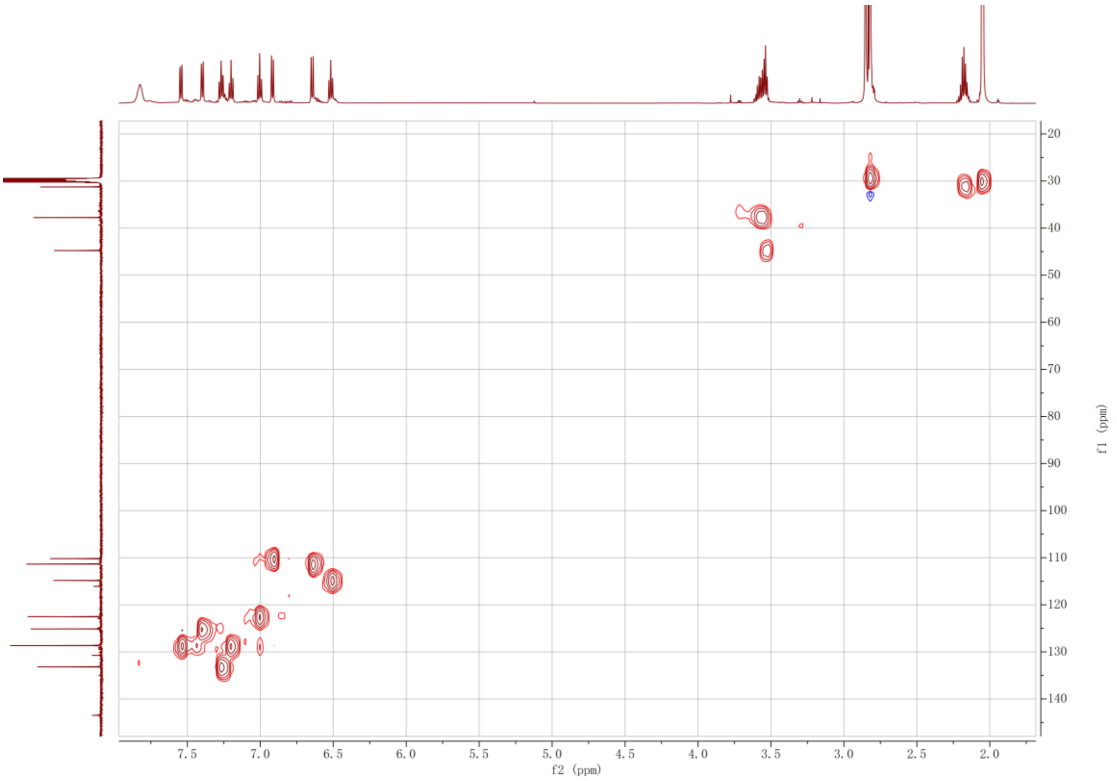

Figure S16. HMBC spectrum of compound **2**

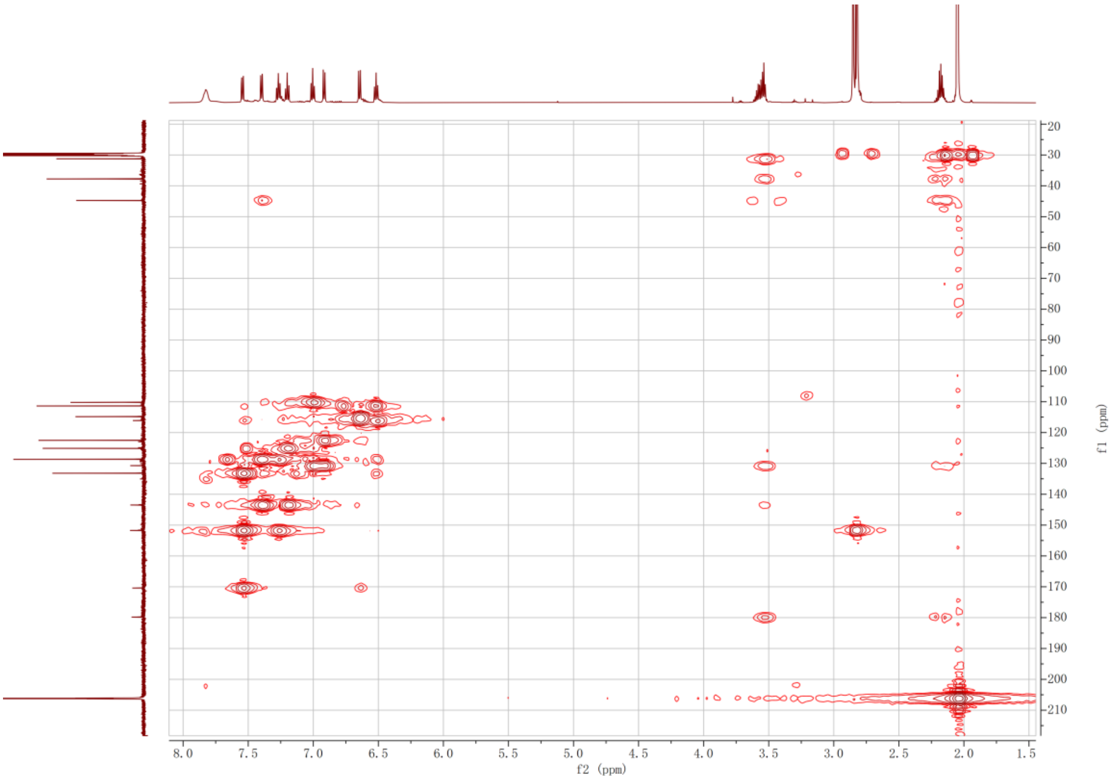

Figure S17. CD spectrum of compound **2**

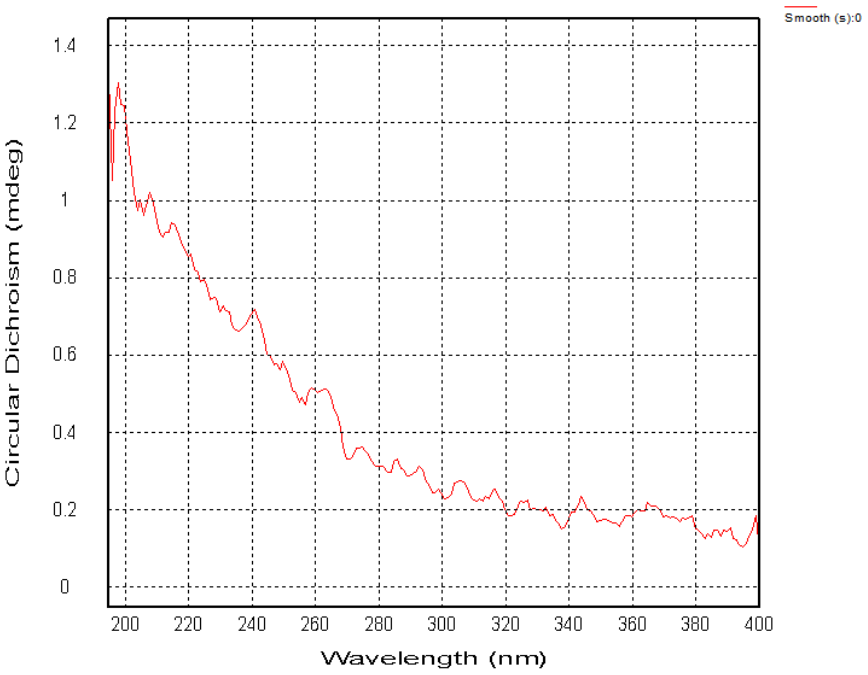

Figure S18. HRESIMS spectrum of compound **3**

Qualitative Analysis Report

**Data Filename**	HCMT-58b.d	**Sample Name**	HCMT-58b
**Sample Type**	Sample	**Position**	P1-F2
**Instrument Name**	Instrument 1	**User Name**	
**Acq Method**	s.m	**Acquired Time**	7/24/2025 3:26:47 PM
**IRM Calibration Status**	Success	**DA Method**	PCDL.m
**Comment**			

**Sample Group**		**Info.**
**Acquisition SW**	6200 series TOF/6500 series	
**Version**	Q-TOF B.05.01 (B5125.2)	

User Spectra

Fragmentor Voltage	Collision Energy	Ionization Mode
135	0	ESI

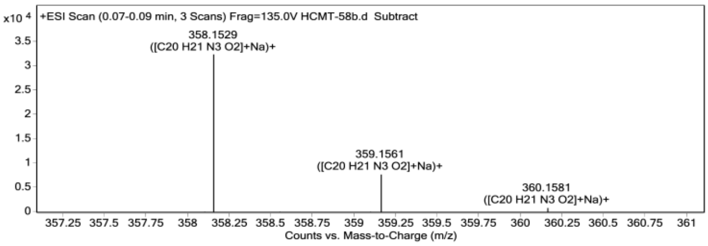

Peak List

m/z	z	Abund	Formula	Ion
334.1554	1	4222.99		
336.1708	1	21530.37		
337.1738	1	5454.51		
358.1529	1	32348.86	C20 H21 N3 O2	(M+Na)+
359.1561	1	7851.24	C20 H21 N3 O2	(M+Na)+
374.1363	1	9439.13		
375.141	1	2258.61		
693.3164	1	19963.39		
694.3203	1	8556.95		
709.2989	1	4431.14		

Formula Calculator Element Limits

Element	Min	Max
C	3	50
H	0	100
O	0	20
N	0	5

Formula Calculator Results

Formula	CalculatedMass	CalculatedMz	Mz	Diff. (mDa)	Diff. (ppm)	DBE
C20 H21 N3 O2	335.1634	358.1526	358.1529	-0.30	-0.84	12.0000

--- End Of Report ---

Figure S19. UV spectrum of compound **3**

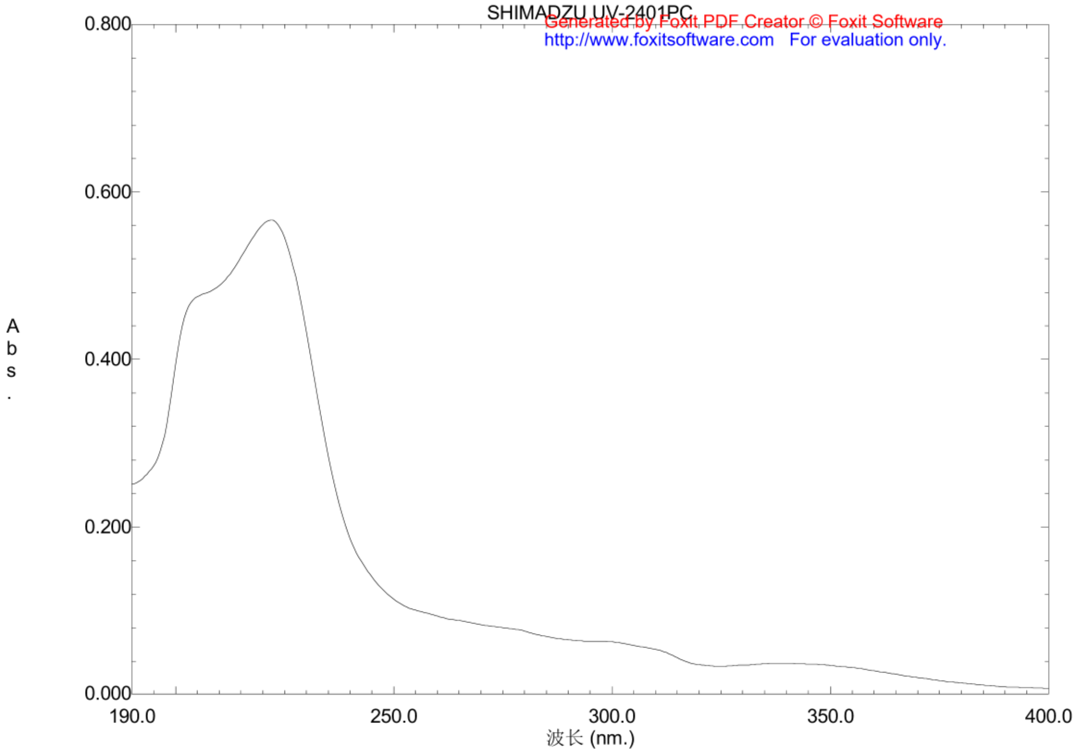

Figure S20. IR spectrum of compound **3**

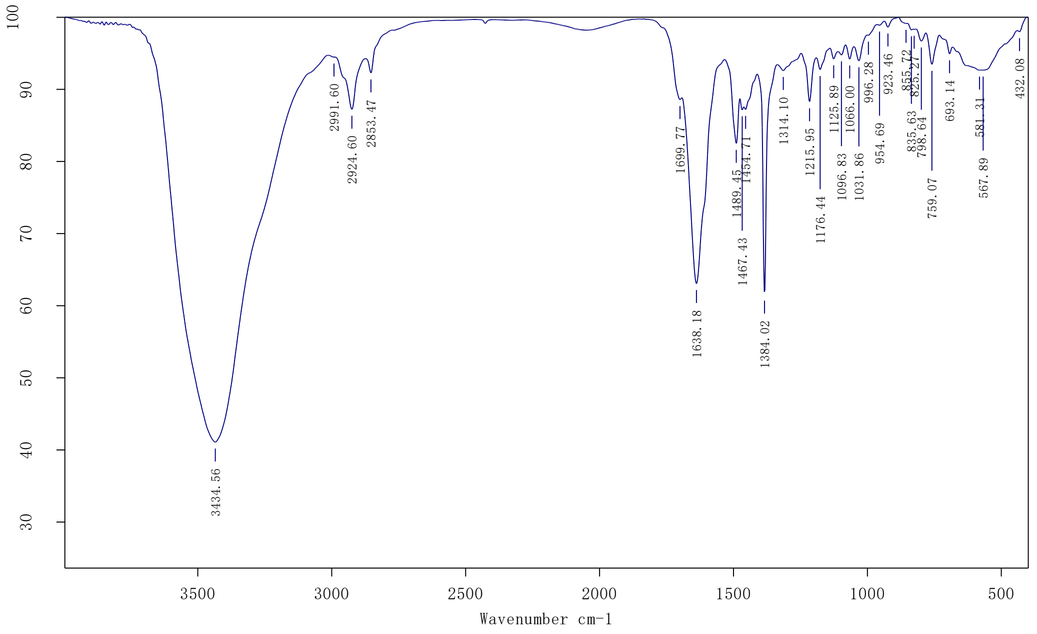

Sample Name: HCMT-58b
Sample Form: KBr
Path of File: E:\data
Date of Measurement: 2025/9/25

Resolution: 4
Aperture Setting: 6 mm
Number of Background Scans: 16
Number of Sample Scans: 16

Beamsplitter Setting: KBr
Source Setting: MIR
Instrument Type: BRUKER VERTEX 70
Soft Version: OPUS8.1

Figure S21. ^{1}H NMR spectrum of compound **3** (600 MHz, CD_3OD)

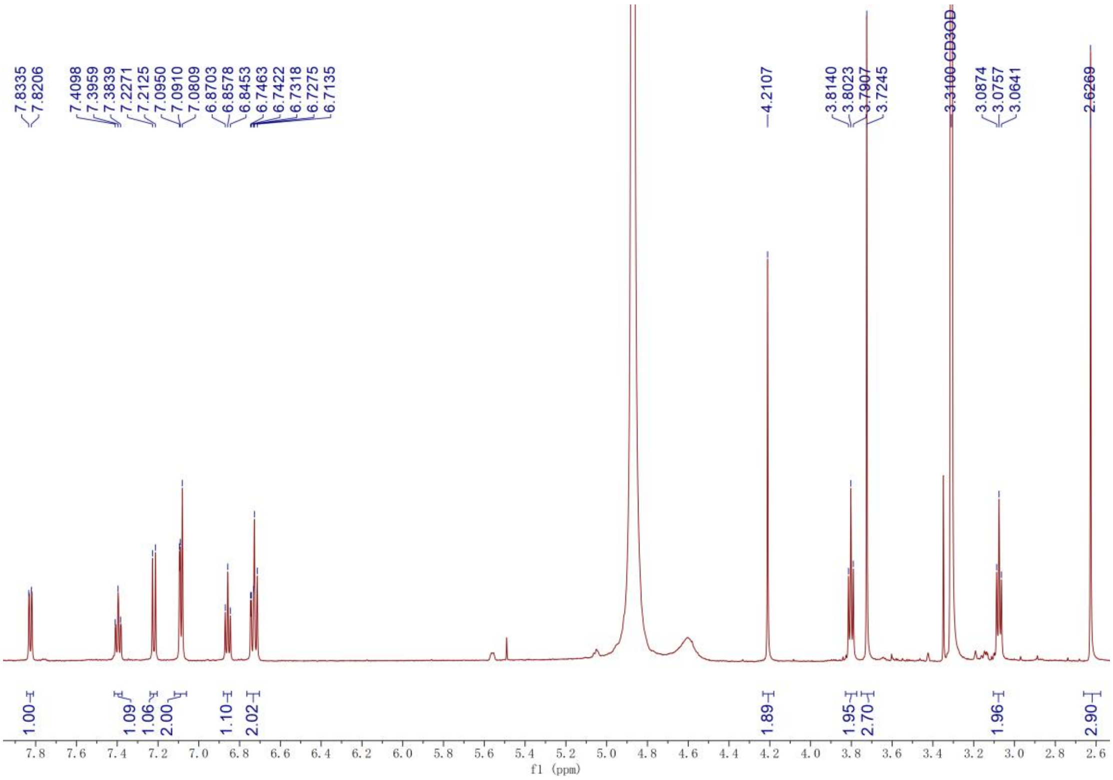

Figure S22. ^{13}C NMR spectrum of compound **3** (150 MHz, CD_3OD)

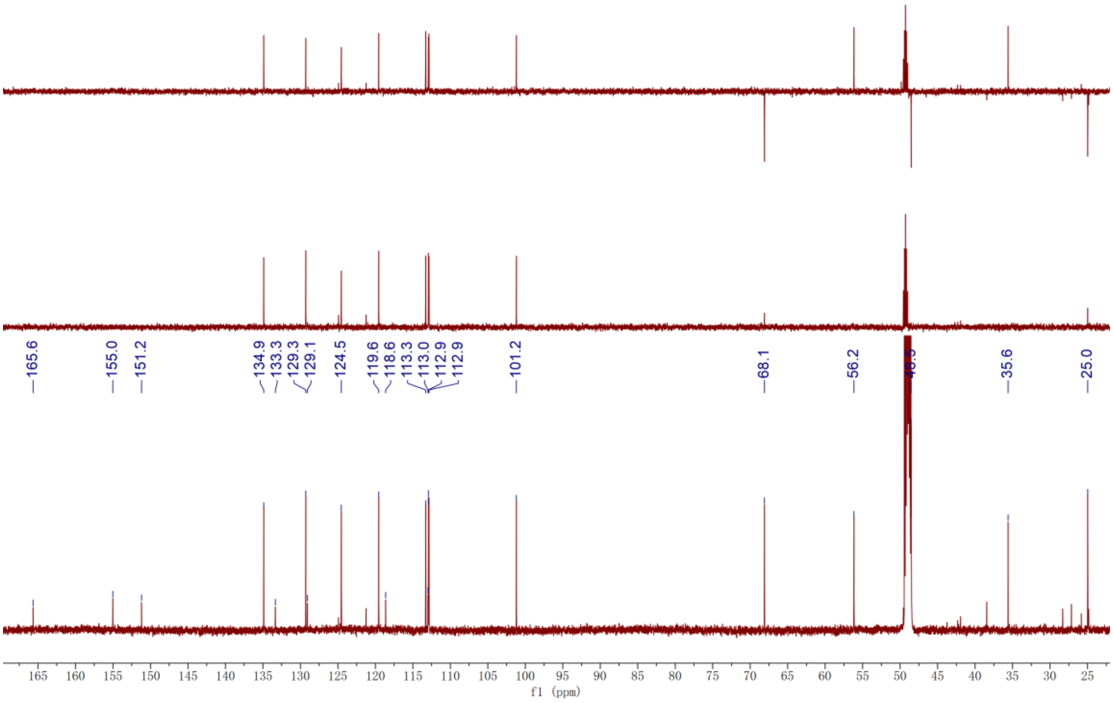

Figure S23. ^1H-^1H COSY spectrum of compound **3**

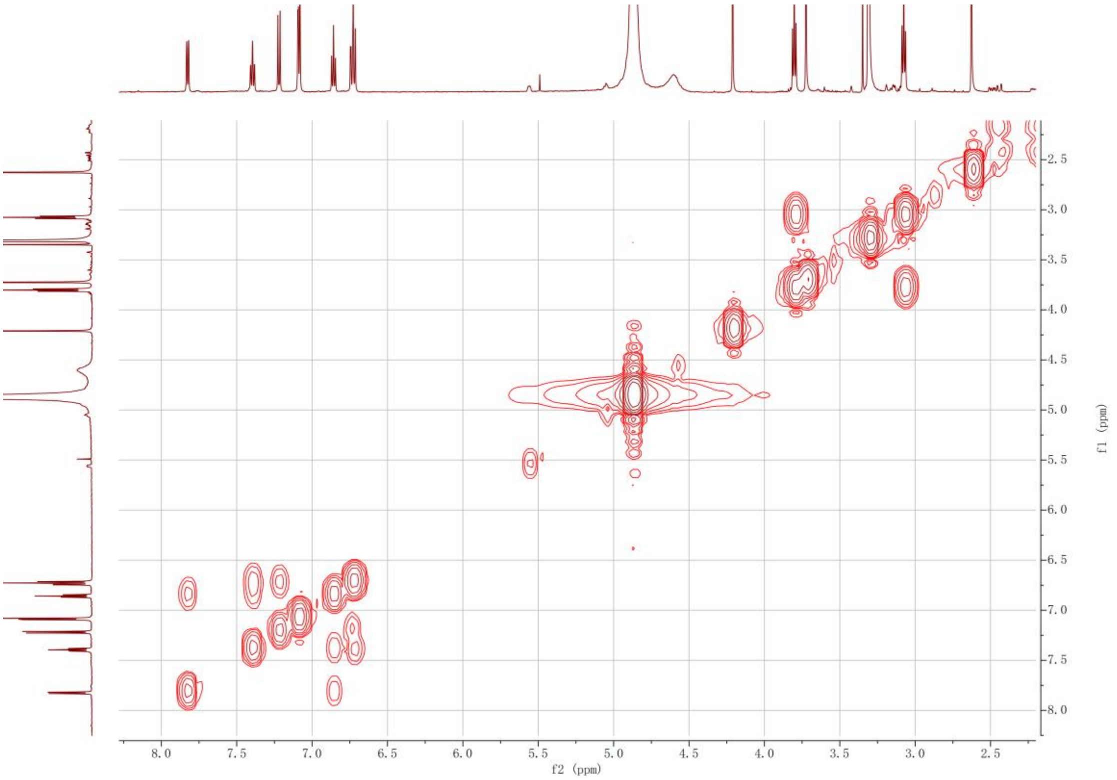

Figure S24. HSQC spectrum of compound **3**

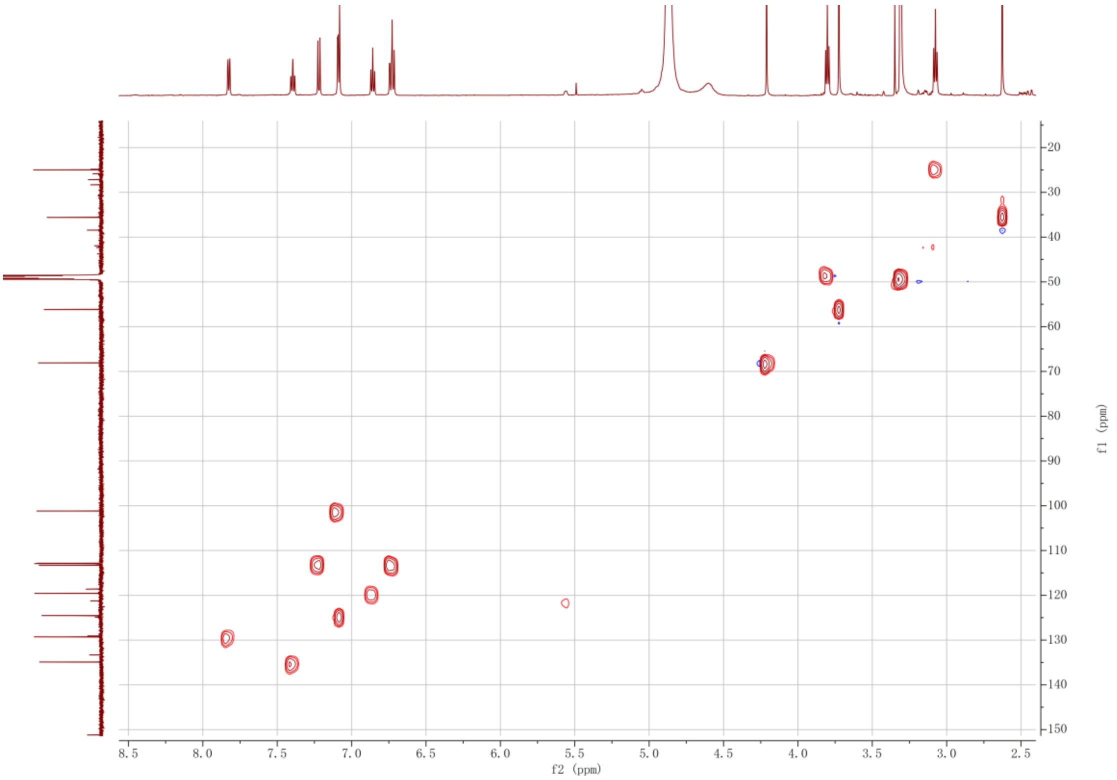

Figure S25. HMBC spectrum of compound **3**

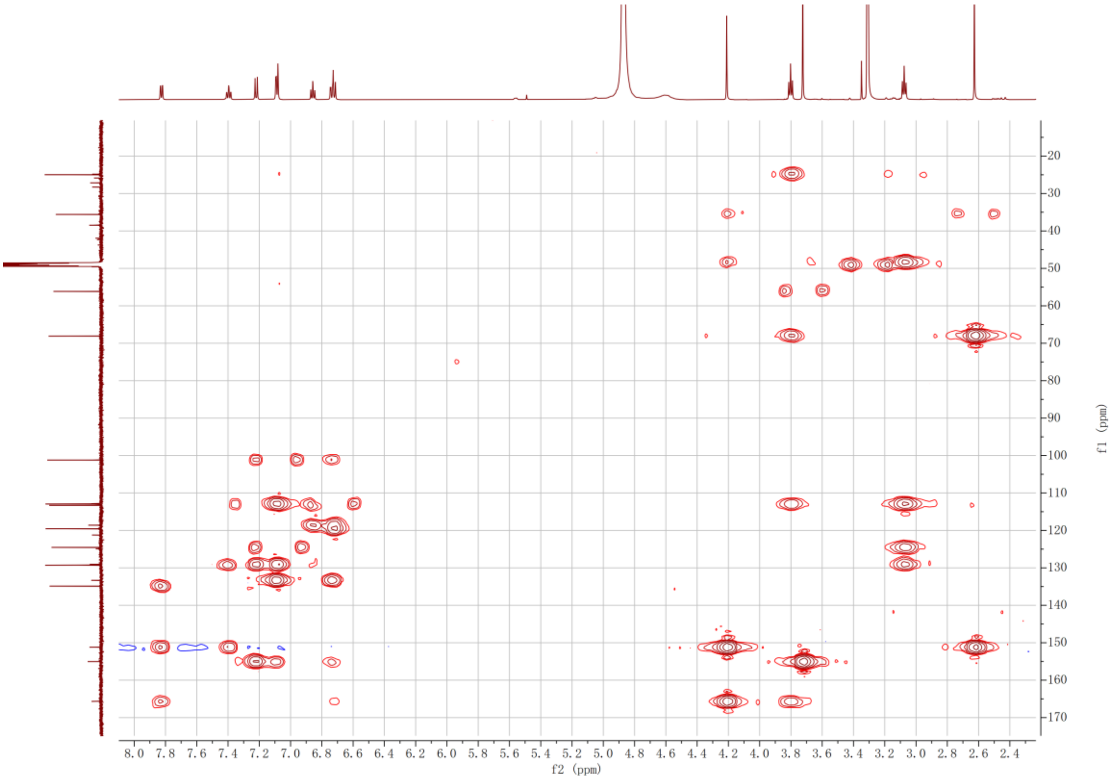

Figure S26. HRESIMS spectrum of compound **4**

Qualitative Analysis Report

**Data Filename**	HCMT-18.d	**Sample Name**	HCMT-18
**Sample Type**	Sample	**Position**	P1-F4
**Instrument Name**	Instrument 1	**User Name**	
**Acq Method**	s.m	**Acquired Time**	5/6/2024 5:17:37 PM
**IRM Calibration Status**	Success	**DA Method**	PCDL.m
**Comment**			

Sample Group **Info.**

Acquisition SW Version 6200 series TOF/6500 series Q-TOF B.05.01 (B5125.2)

User Spectra

Fragmentor Voltage	Collision Energy	Ionization Mode
135	0	ESI

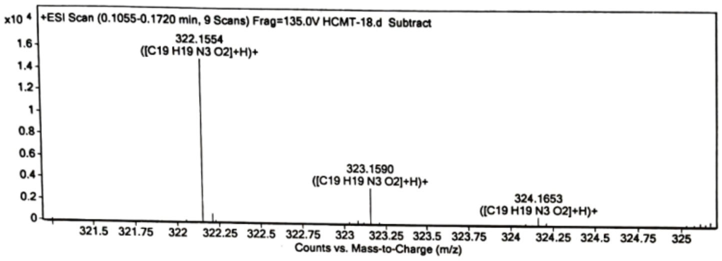

Peak List

m/z	z	Abund	Formula	Ion
104.1071	1	27260.55		
118.0865	1	8780.67		
161.1073	1	8432.29		
163.123	1	6584.61		
172.0756	1	13985.46		
177.1023	1	40828.26		
179.1178	1	17234.44		
258.1492	1	12958.81		
306.1342	1	6595.35		
322.1554	1	14916.19	C19 H19 N3 O2	(M+H)+

Formula Calculator Element Limits

Element	Min	Max
C	3	60
H	0	100
O	0	20
N	0	3

Formula Calculator Results

Formula	CalculatedMass	CalculatedMz	Mz	Diff. (mDa)	Diff. (ppm)	DBE
C19 H19 N3 O2	321.1477	322.1550	322.1554	-0.40	-1.24	12.0000

--- End Of Report ---

Agilent Technologies Page 1 of 1 Printed at: 5:20 PM on: 5/6/2024

Figure S27. UV spectrum of compound **4**

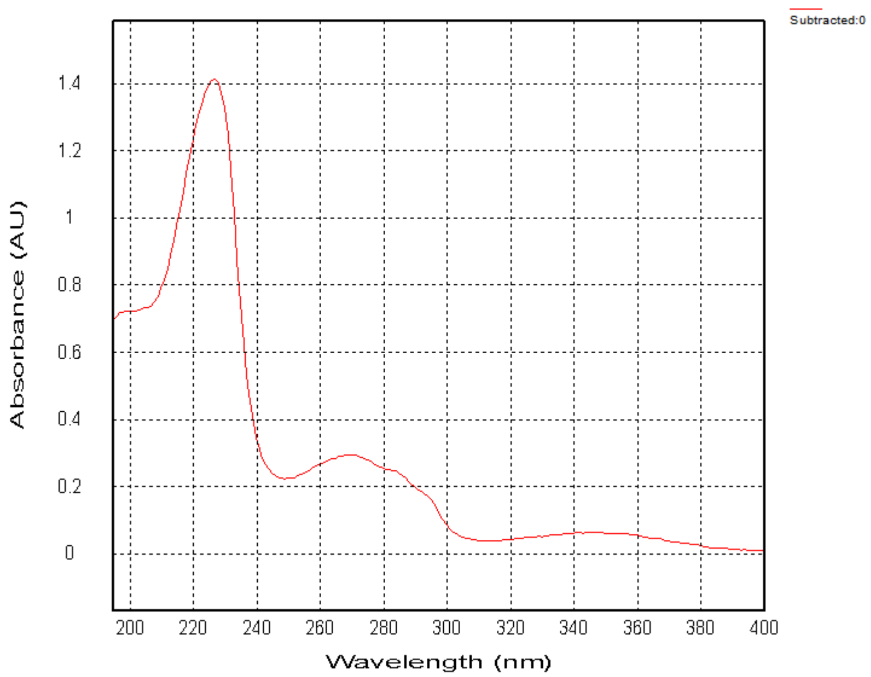

Figure S28. IR spectrum of compound **4**

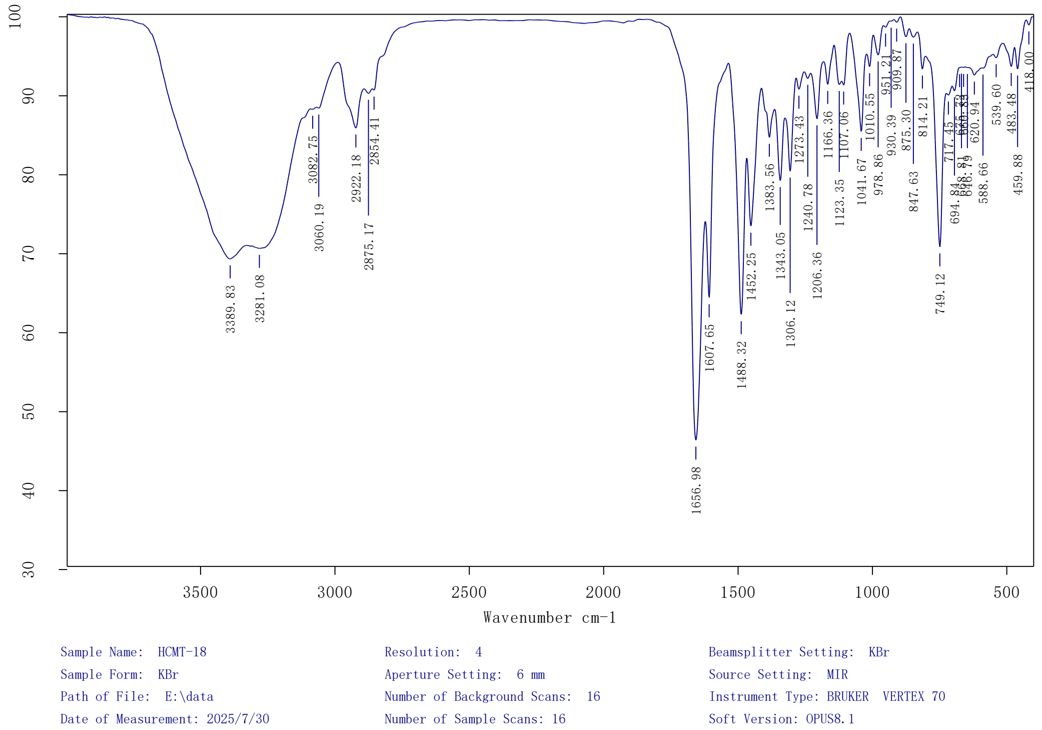

Figure S29. ^{1}H NMR spectrum of compound **4** (500 MHz, CD_3OD)

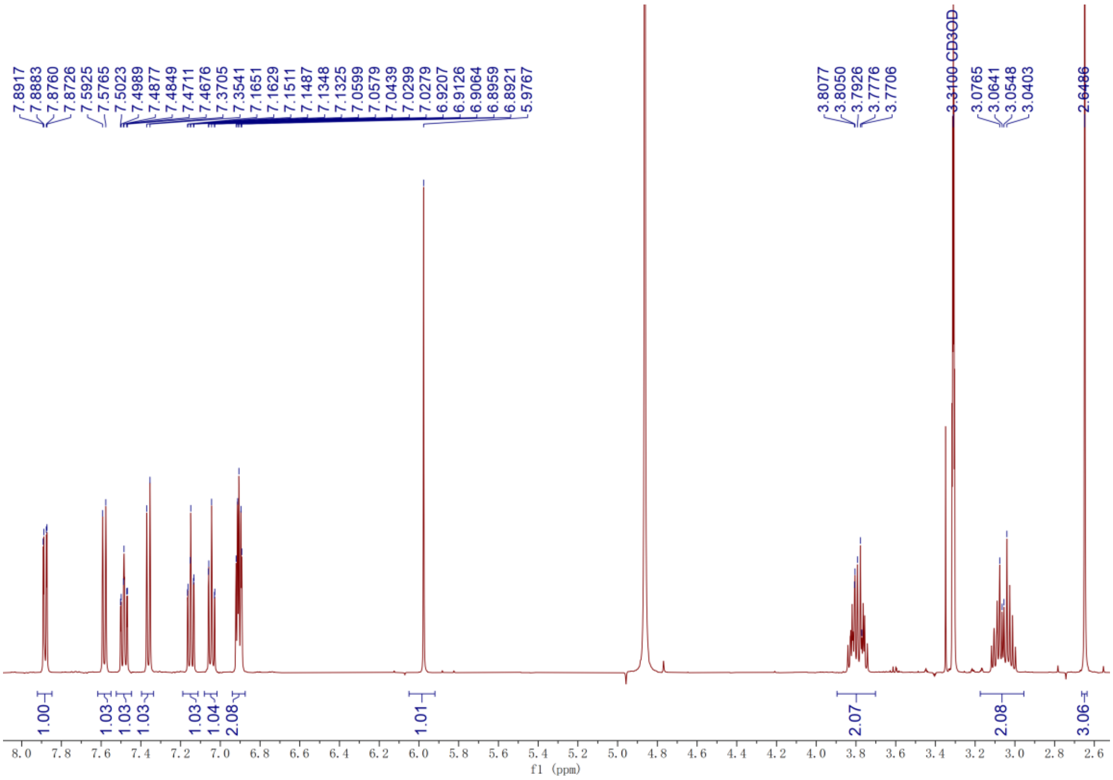

Figure S30. ^{13}C NMR spectrum of compound **4** (125 MHz, CD_3OD)

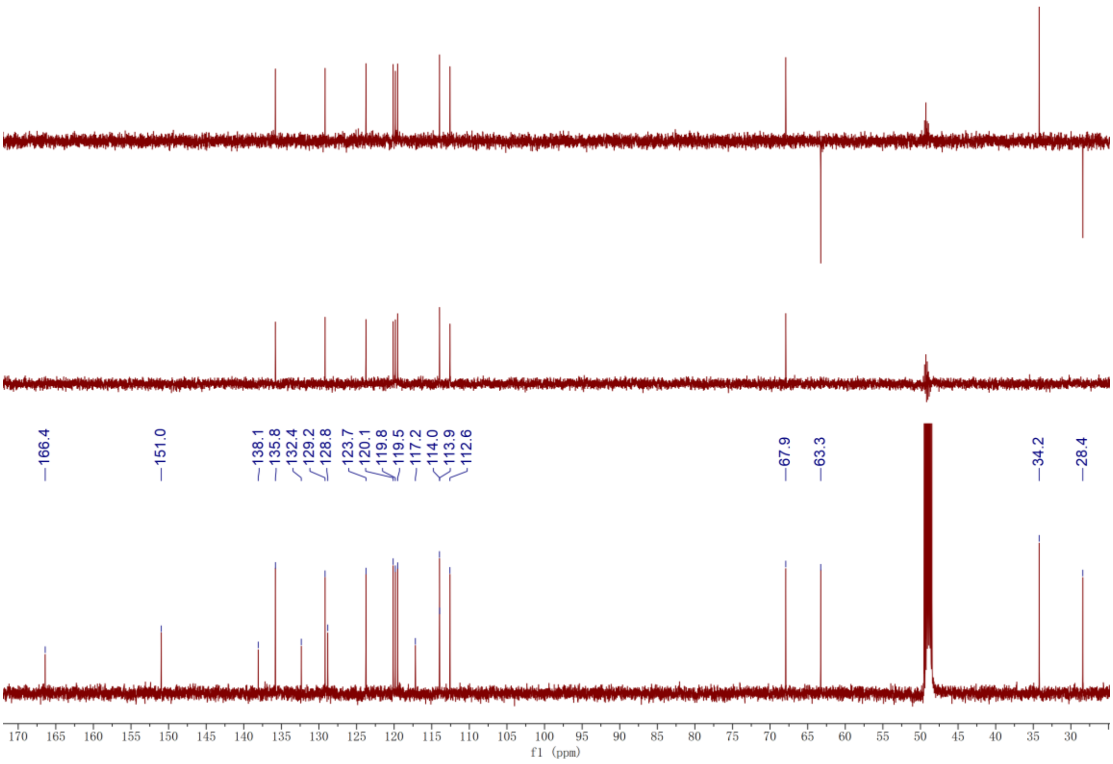

Figure S31. ^{1}H-^{1}H COSY spectrum of compound **4**

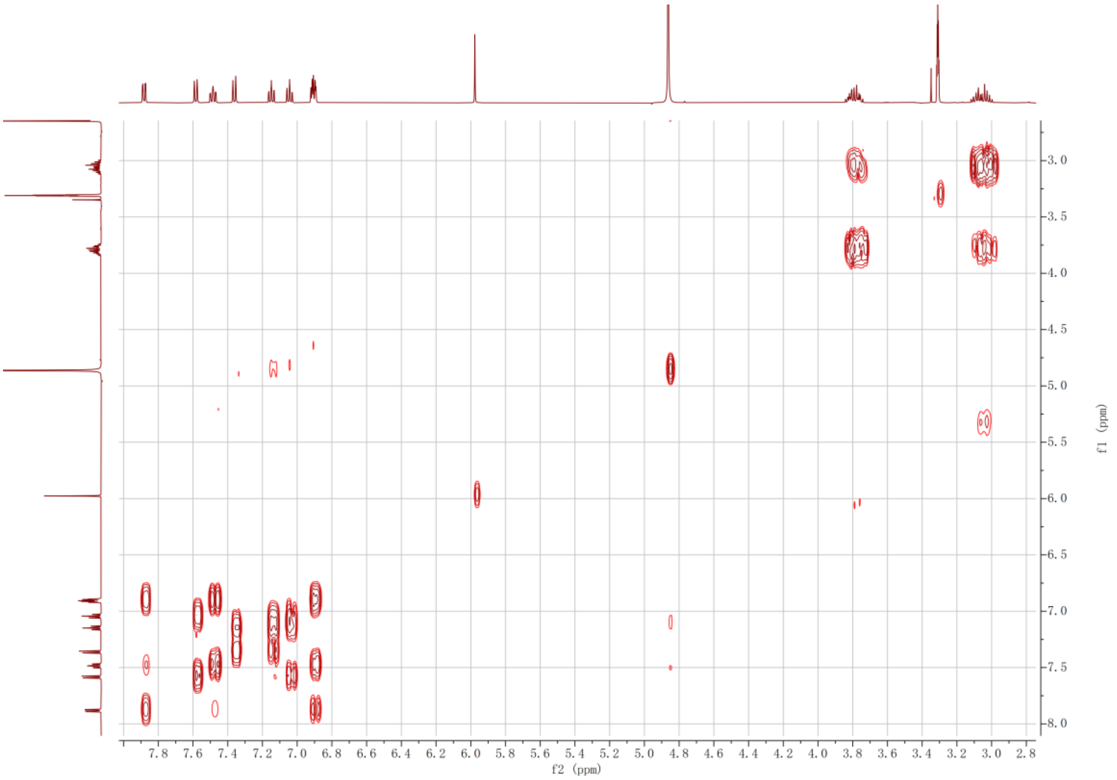

Figure S32. HSQC spectrum of compound **4**

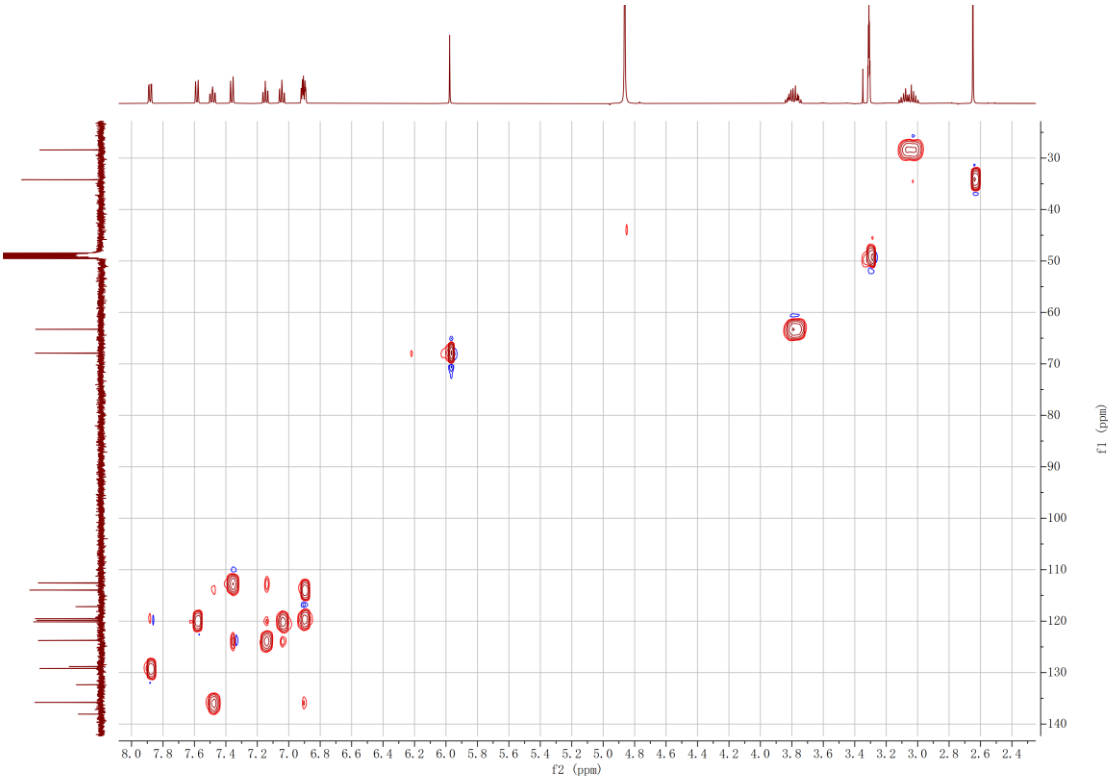

Figure S33. HMBC spectrum of compound **4**

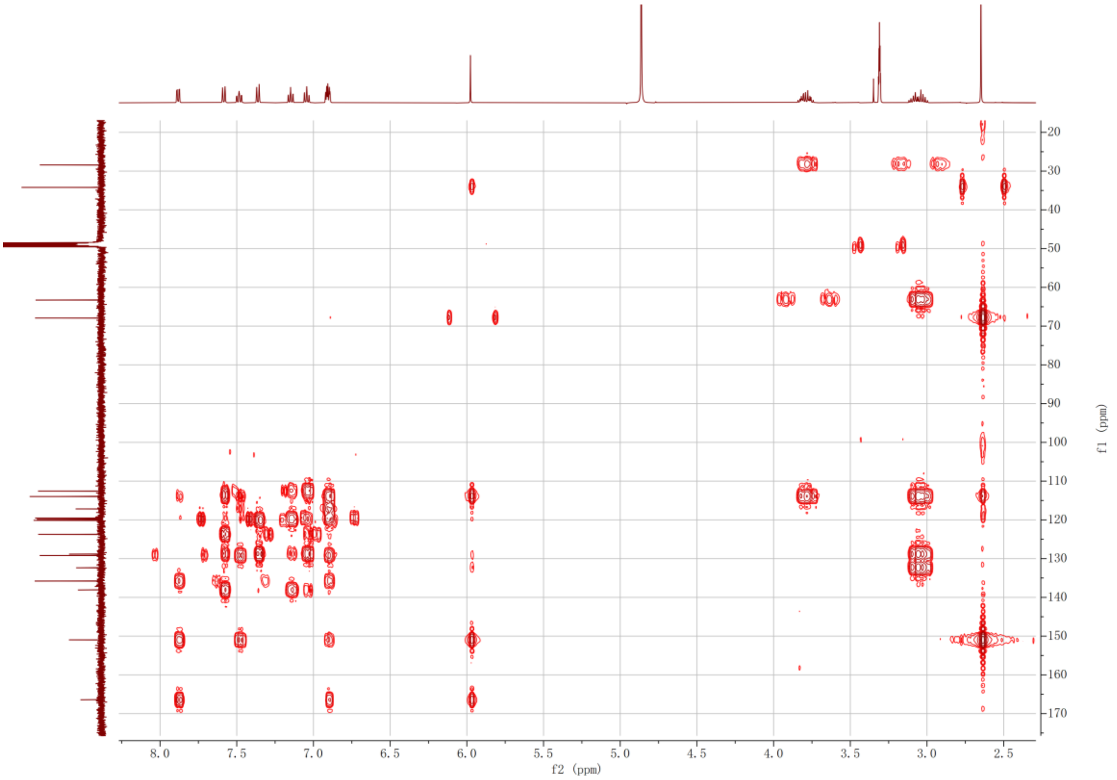

Figure S34. CD spectrum of compound **4**

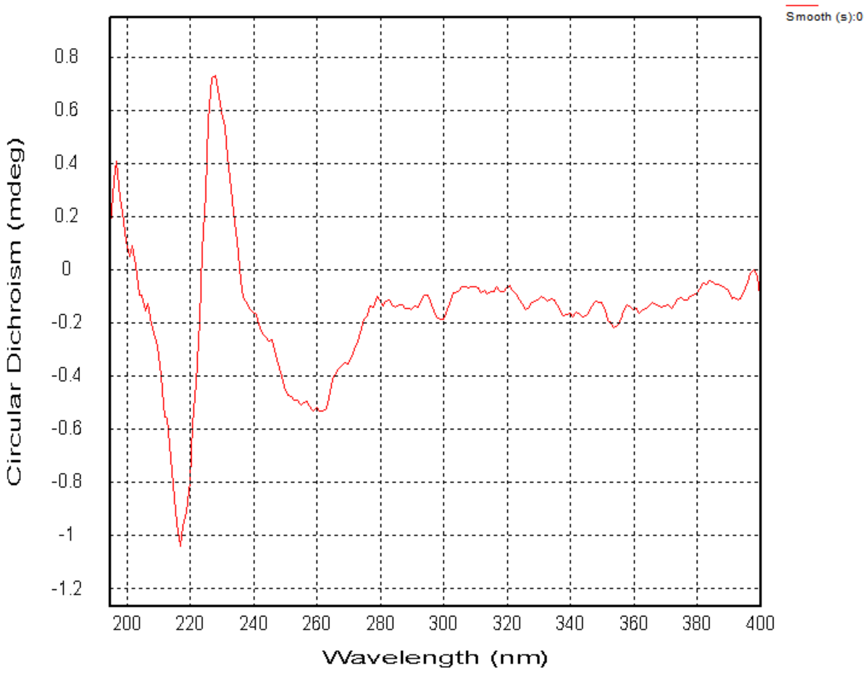

Figure S35. HRESIMS spectrum of compound **23**

Qualitative Analysis Report

**Data Filename**	HCMT-36b.d	**Sample Name**	HCMT-36b
**Sample Type**	Sample	**Position**	P1-A9
**Instrument Name**	Instrument 1	**User Name**	
**Acq Method**	s.m	**Acquired Time**	9/26/2024 11:19:31 AM
**IRM Calibration Status**	Success	**DA Method**	PCDL.m
**Comment**			

Info.

**Sample Group**	
**Acquisition SW Version**	6200 series TOF/6500 series Q-TOF B.05.01 (B5125.2)

User Spectra

Fragmentor Voltage	Collision Energy	Ionization Mode
135	0	ESI

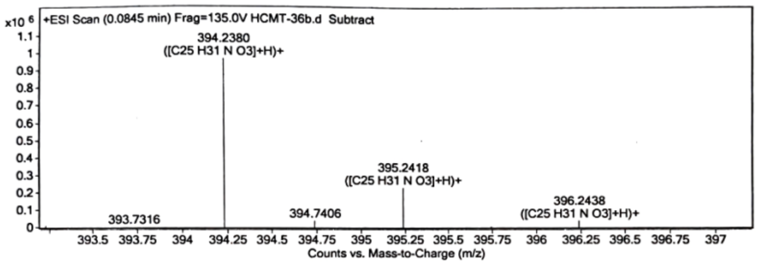

Peak List

m/z	z	Abund	Formula	Ion
376.227	1	17801.75		
392.2221	1	16563.09		
394.238	1	967957.88	C25 H31 N O3	(M+H)+
394.7406	1	37604.83		
395.2418	1	232121.91	C25 H31 N O3	(M+H)+
396.2438	1	38069.69	C25 H31 N O3	(M+H)+
408.2173	1	19812.64		
410.2325	1	53252.6		
411.2356	1	14907.15		
787.4679	1	13957.93		

Formula Calculator Element Limits

Element	Min	Max
C	3	60
H	0	200
O	0	20
N	0	5

Formula Calculator Results

Formula	CalculatedMass	CalculatedMz	Mz	Diff. (mDa)	Diff. (ppm)	DBE
C25 H31 N O3	393.2304	394.2377	394.2380	-0.30	-0.76	11.0000

--- End Of Report ---

Agilent Technologies   Page 1 of 1   Printed at: 11:21 AM on: 9/26/2024

Figure S36. UV spectrum of compound **23**

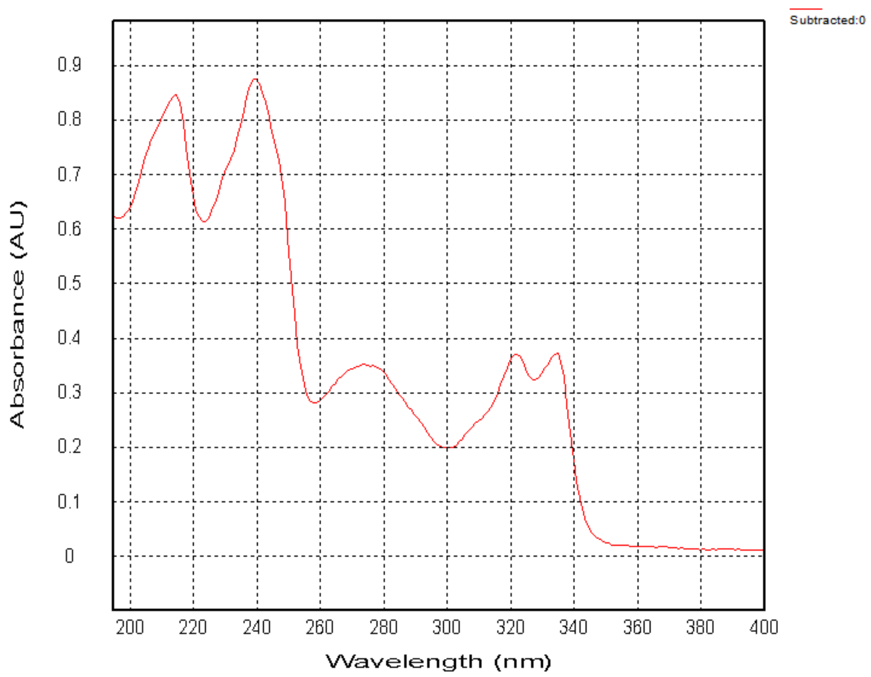

Figure S37. IR spectrum of compound **23**

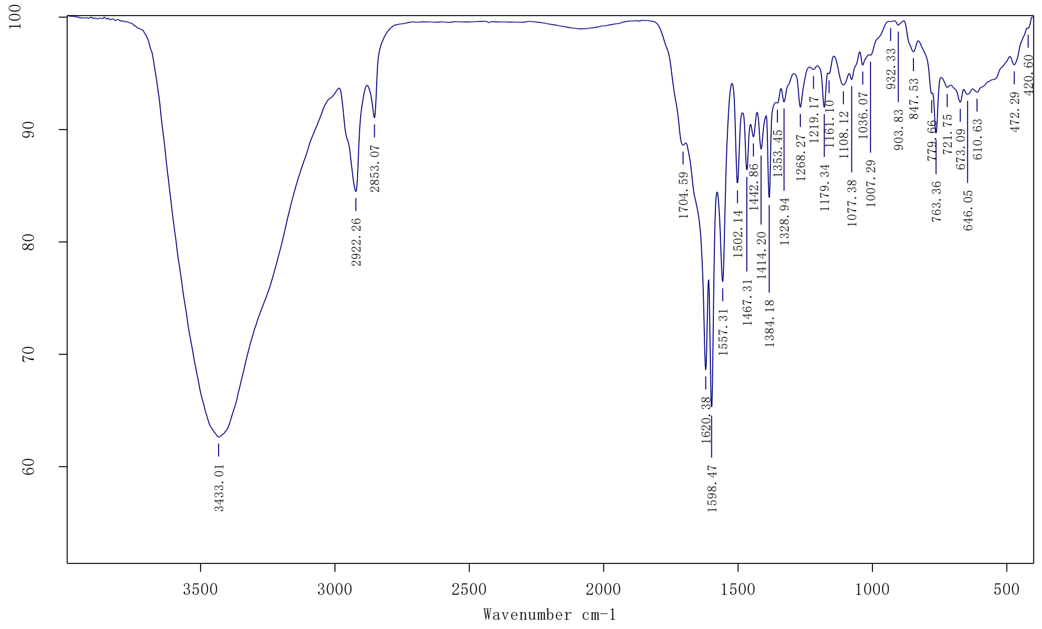

Sample Name: HCMT-36b
Sample Form: KBr
Path of File: E:\data
Date of Measurement: 2025/7/30

Resolution: 4
Aperture Setting: 6 mm
Number of Background Scans: 16
Number of Sample Scans: 16

Beamsplitter Setting: KBr
Source Setting: MIR
Instrument Type: BRUKER VERTEX 70
Soft Version: OPUS8.1

Figure S38. ^{1}H NMR spectrum of compound **23** (500 MHz, CD_3OD)

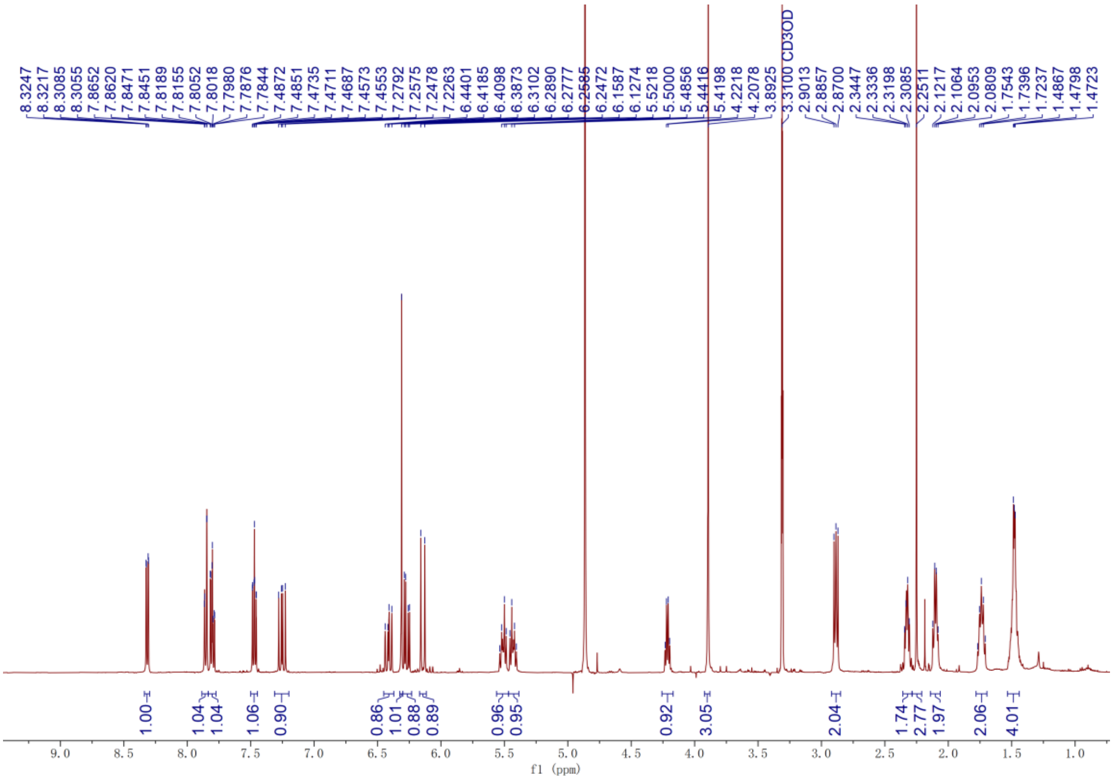

Figure S39. ^{13}C NMR spectrum of compound **23** (125 MHz, CD_3OD)

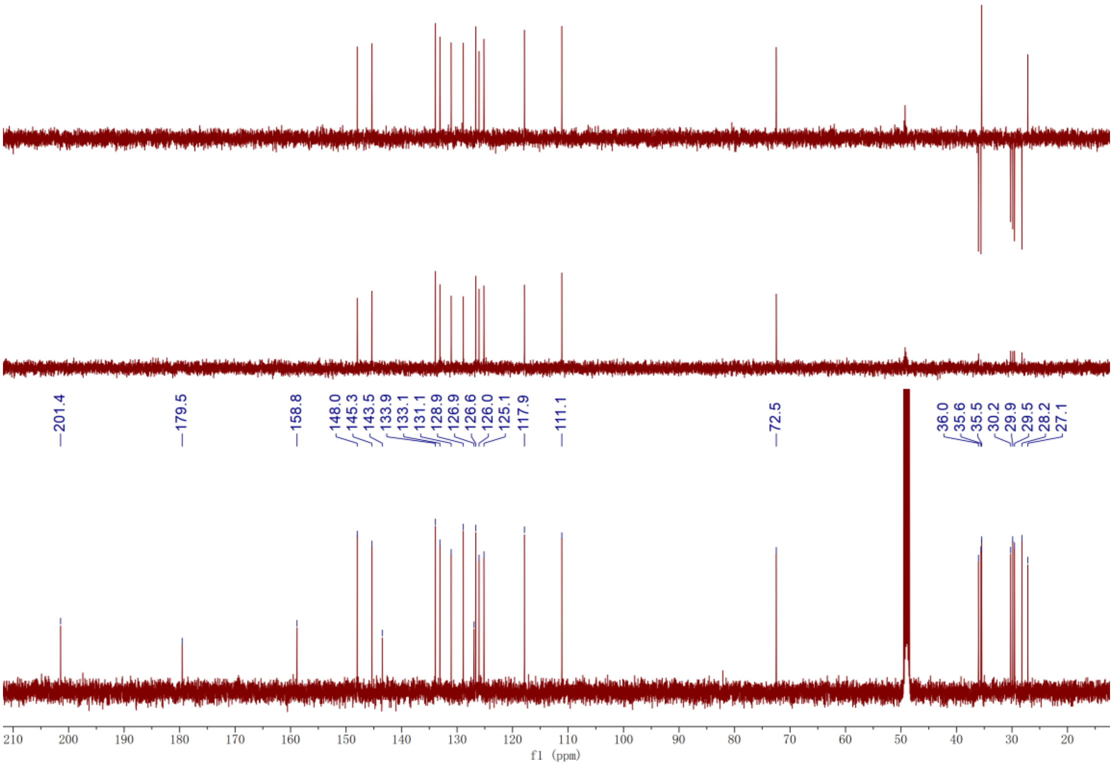

Figure S40. ^{1}H-^{1}H COSY spectrum of compound **23**

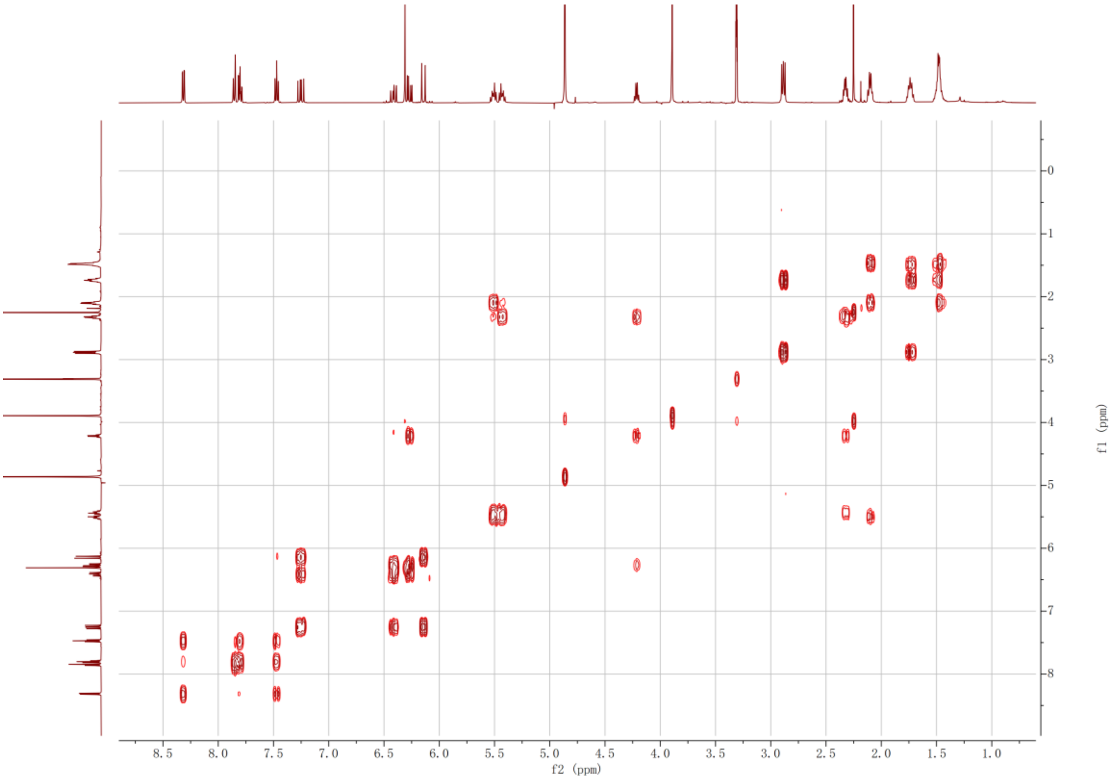

Figure S41. HSQC spectrum of compound **23**

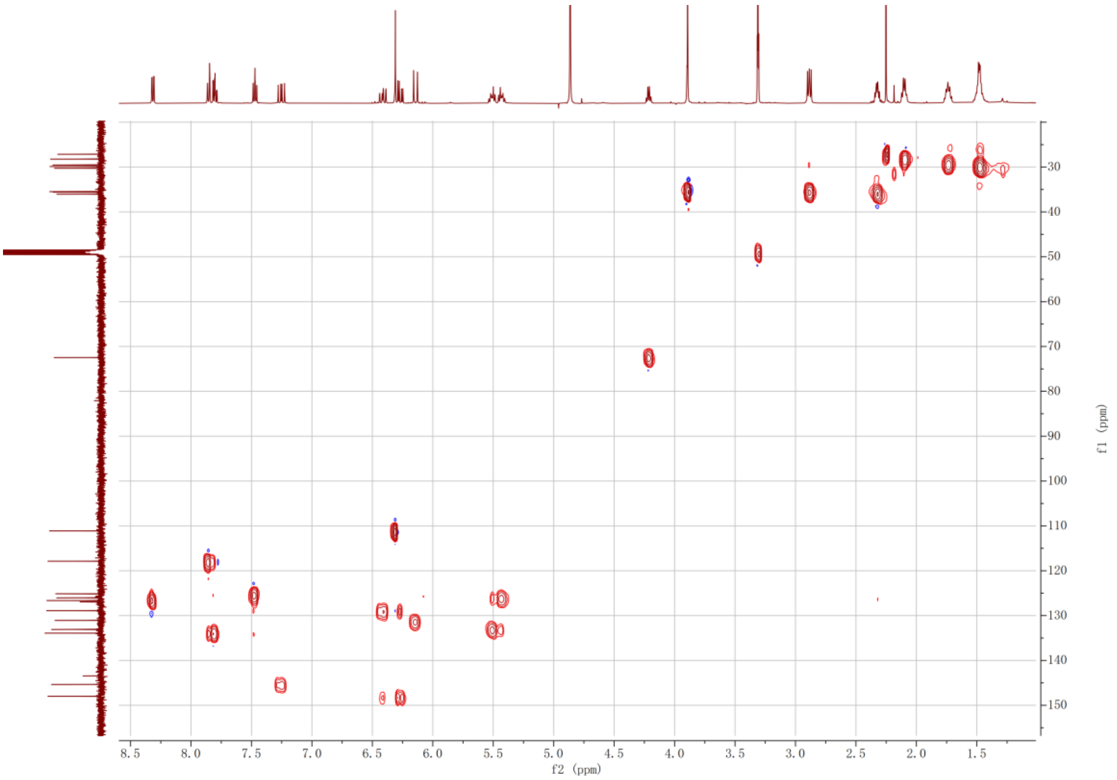

Figure S42. HMBC spectrum of compound **23**

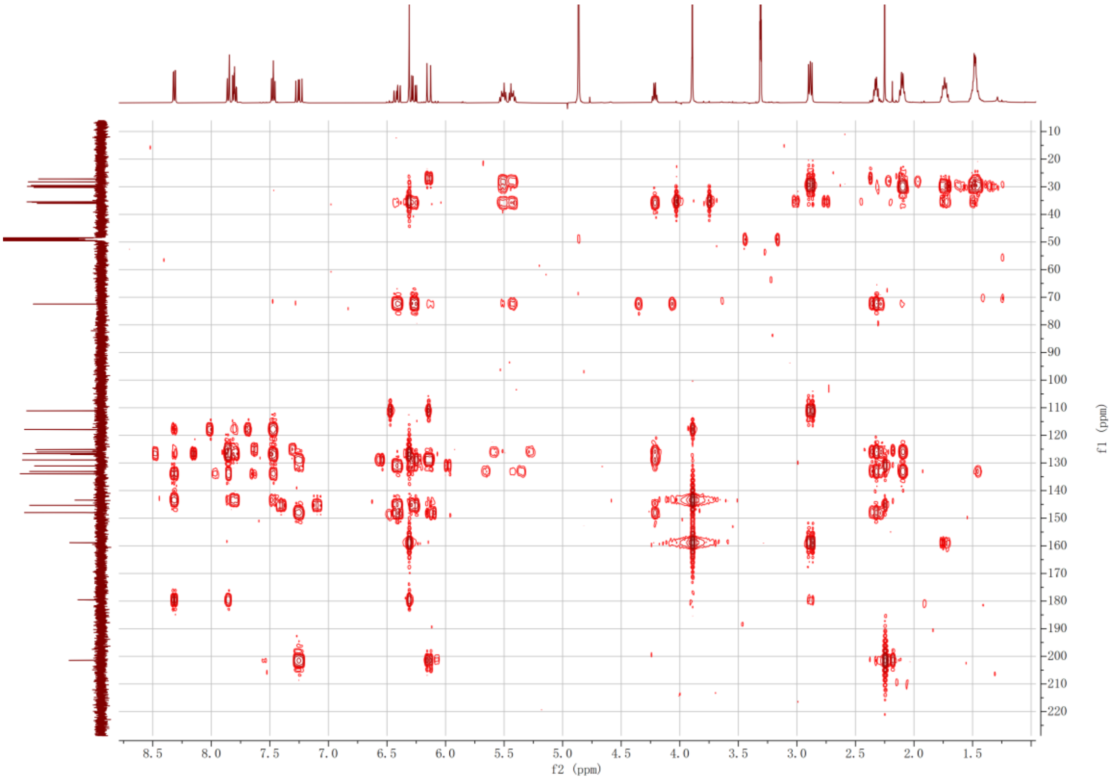

Figure S43. CD spectrum of compound **23**

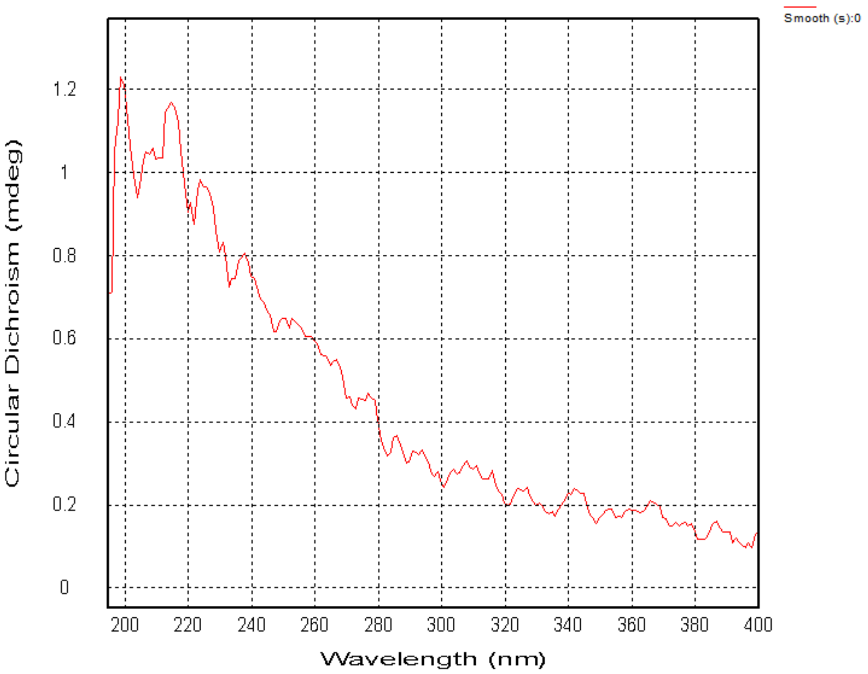

Figure S44. HRESIMS spectrum of compound **29**

Qualitative Analysis Report

**Data Filename**	HCMT-46b.d	**Sample Name**	HCMT-46b
**Sample Type**	Sample	**Position**	P1-F4
**Instrument Name**	Instrument 1	**User Name**	
**Acq Method**	s.m	**Acquired Time**	12/23/2024 11:07:15 AM
**IRM Calibration Status**	Success	**DA Method**	PCDL.m
**Comment**			

Sample Group **Info.**

Acquisition SW 6200 series TOF/6500 series

Version Q-TOF B.05.01 (B5125.2)

User Spectra

Fragmentor Voltage	Collision Energy	Ionization Mode
175	0	ESI

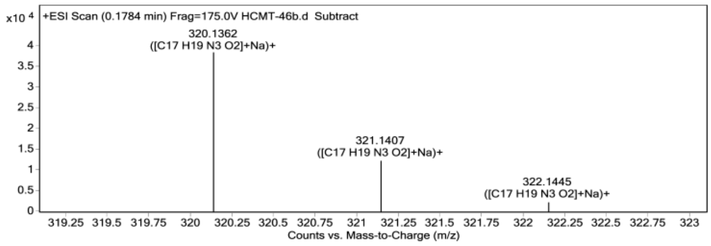

Peak List

m/z	z	Abund	Formula	Ion
102.1276	1	10063.9		
134.0597	1	19850.83		
172.0938	1	6616.67		
298.1546	1	26707.29		
299.159	1	6743		
320.1362	1	38327.79	C17 H19 N3 O2	(M+Na)+
321.1407	1	12446.69	C17 H19 N3 O2	(M+Na)+
617.2837	1	17866.36		
618.2889	1	11947.55		
619.2927	1	3511.12		

Formula Calculator Element Limits

Element	Min	Max
C	3	60
H	0	150
O	0	20
N	0	5

Formula Calculator Results

Formula	CalculatedMass	CalculatedMz	Mz	Diff. (mDa)	Diff. (ppm)	DBE
C17 H19 N3 O2	297.1477	320.1369	320.1362	0.70	2.19	10.0000

--- End Of Report ---

Figure S45. UV spectrum of compound **29**

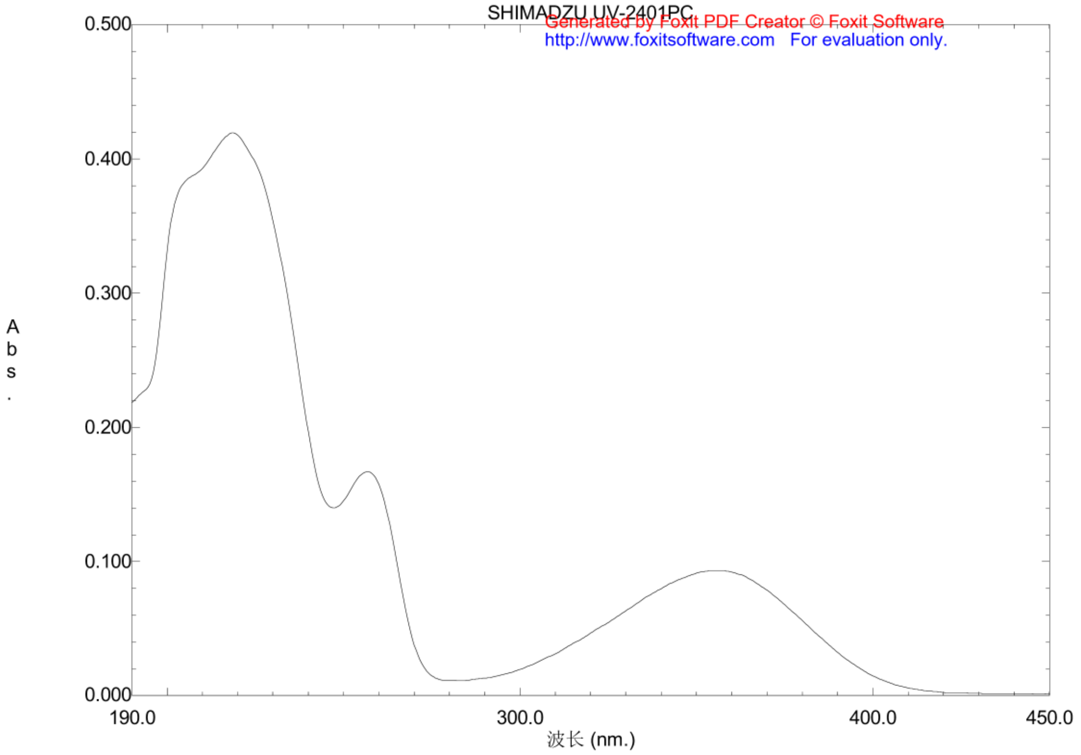

Figure S46. IR spectrum of compound **29**

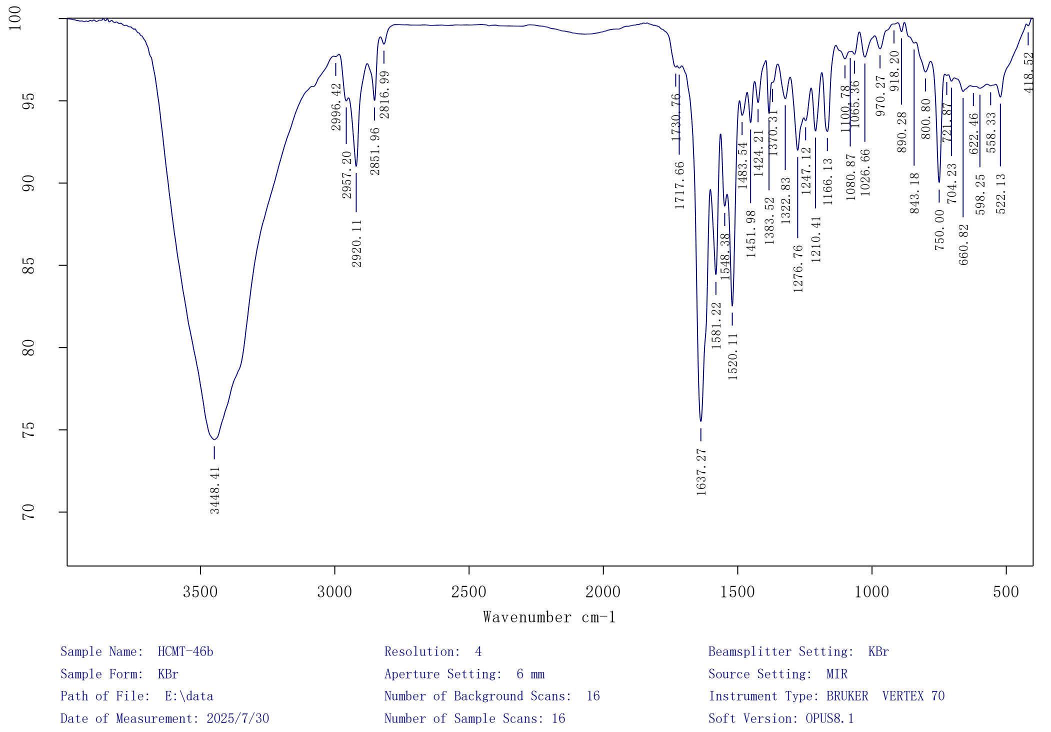

Figure S47. ^{1}H NMR spectrum of compound **29** (600 MHz, CD_3OD)

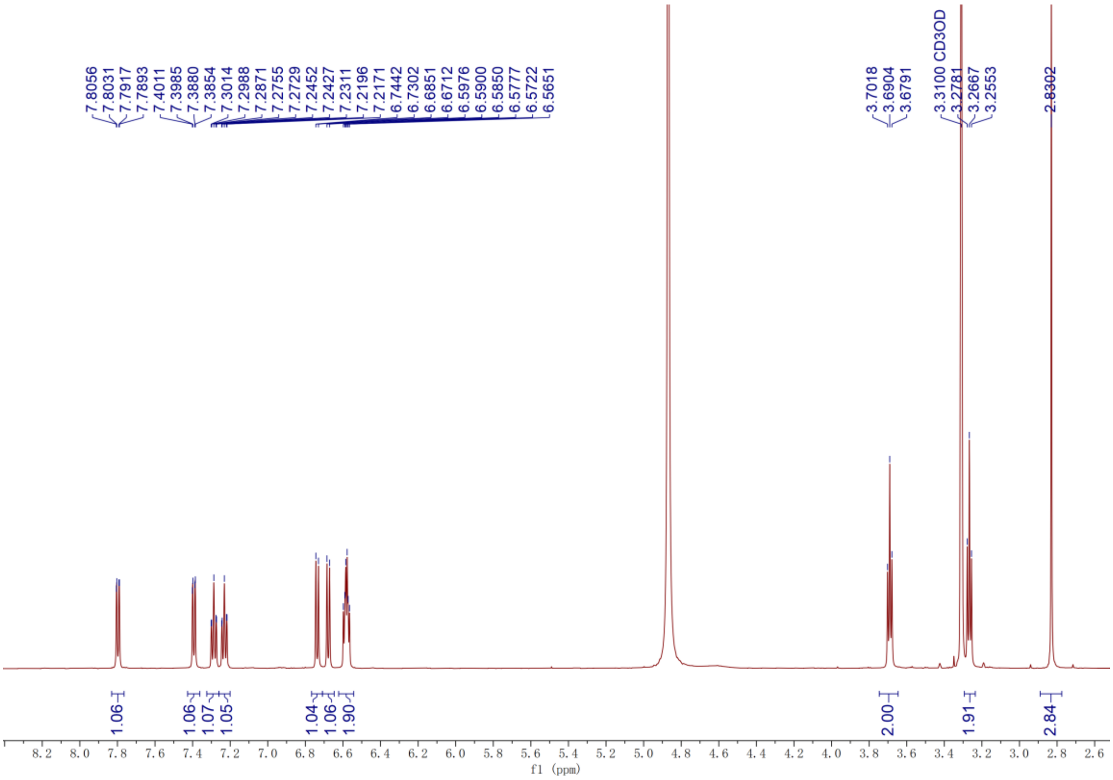

Figure S48. ^{13}C NMR spectrum of compound **29** (150 MHz, CD_3OD)

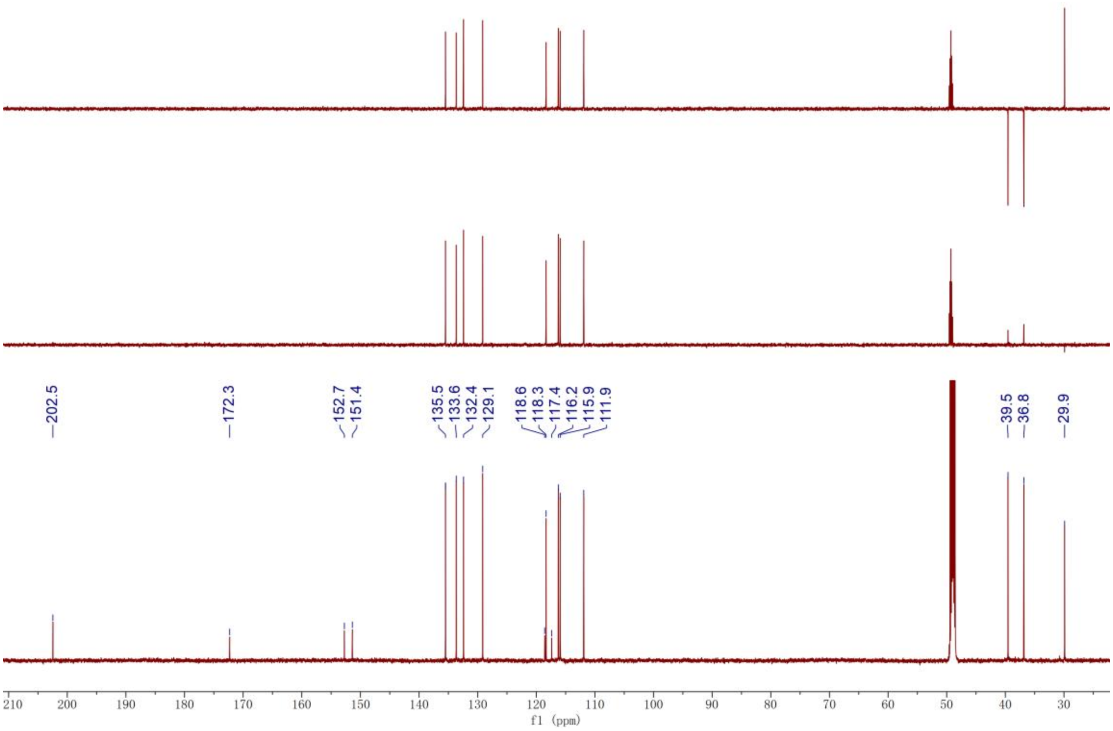

Figure S49. ^{1}H-^{1}H COSY spectrum of compound **29**

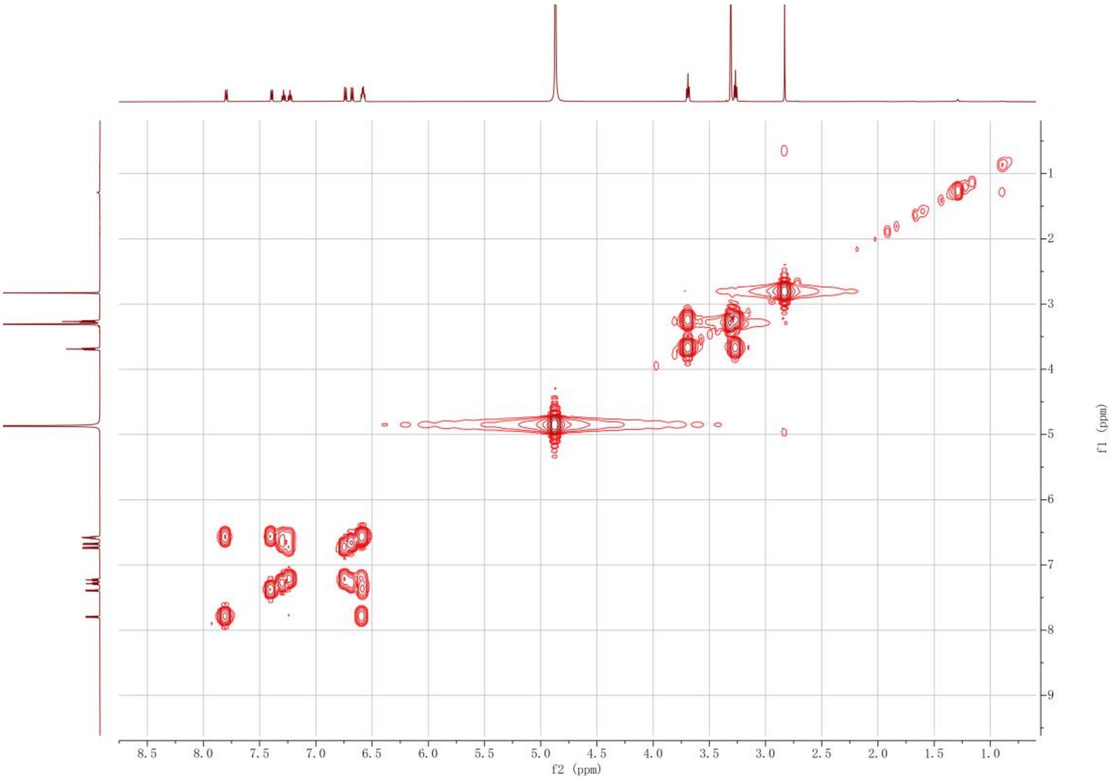

Figure S50. HSQC spectrum of compound **29**

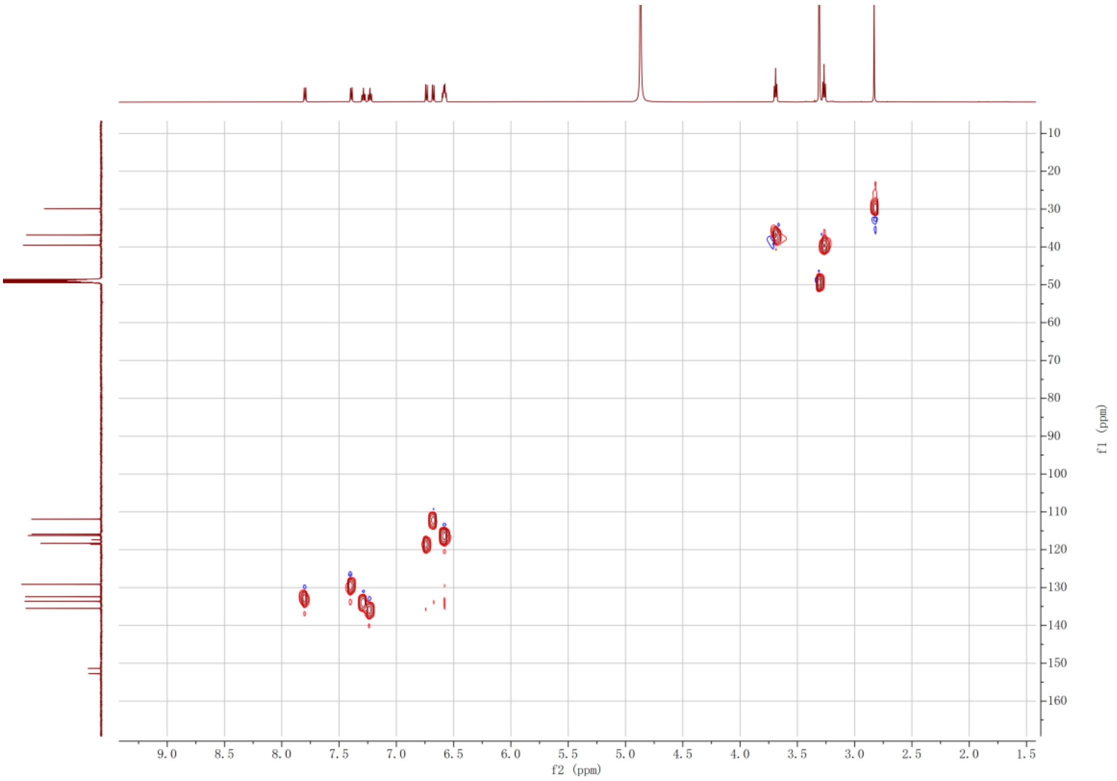

Figure S51. HMBC spectrum of compound **29**

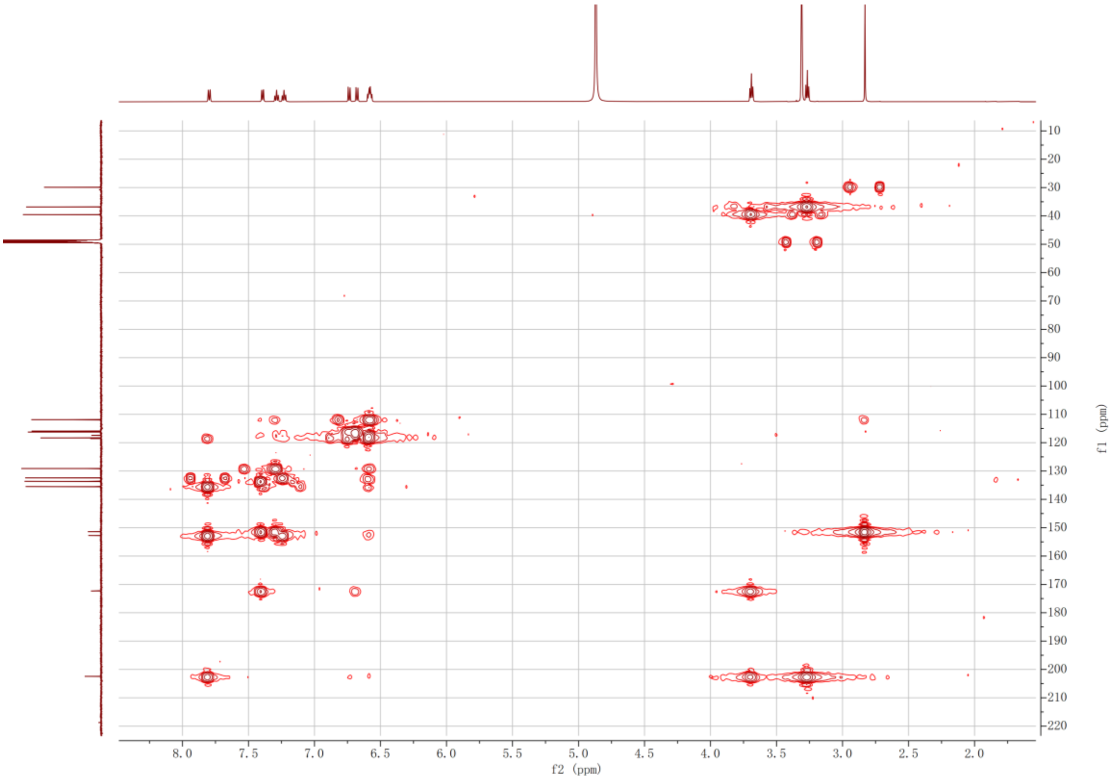

Figure S52. HRESIMS spectrum of compound **30**

Qualitative Analysis Report

**Data Filename**	HCMT-15.d	**Sample Name**	HCMT-15
**Sample Type**	Sample	**Position**	P1-A3
**Instrument Name**	Instrument 1	**User Name**	
**Acq Method**	s.m	**Acquired Time**	4/9/2024 4:41:33 PM
**IRM Calibration Status**	Success	**DA Method**	PCDL.m
**Comment**			

Sample Group **Info.**
Acquisition SW 6200 series TOF/6500 series
Version Q-TOF B.05.01 (B5125.2)

User Spectra

Fragmentor Voltage	Collision Energy	Ionization Mode
135	0	ESI

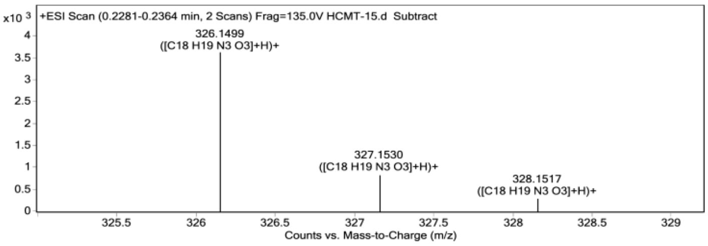

Peak List

m/z	z	Abund	Formula	Ion
61.04	1	665.2		
71.0608	1	1023.85		
81.5212	1	1180.19		
89.507	1	1362.39		
102.0349	1	2526.13		
134.0603	1	604.55		
326.1499	1	3635.25	C18 H19 N3 O3	(M+H)+
327.153	1	851.76	C18 H19 N3 O3	(M+H)+
348.1312	1	1673.52		
356.1855	1	587.57		

Formula Calculator Element Limits

Element	Min	Max
C	3	150
H	0	250
O	0	30
N	0	5

Formula Calculator Results

Formula	CalculatedMass	CalculatedMz	Mz	Diff. (mDa)	Diff. (ppm)	DBE
C18 H19 N3 O3	325.1426	326.1499	326.1499	0.00	0.00	11.0000

--- End Of Report ---

Figure S53. UV spectrum of compound **30**

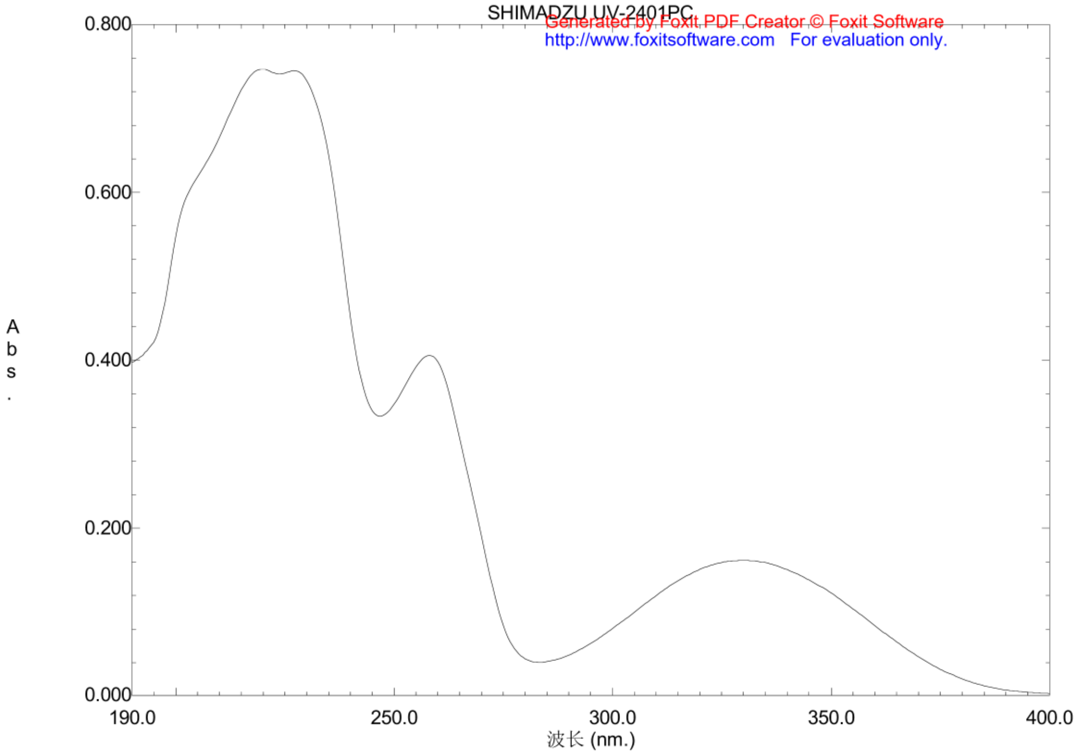

Figure S54. IR spectrum of compound **30**

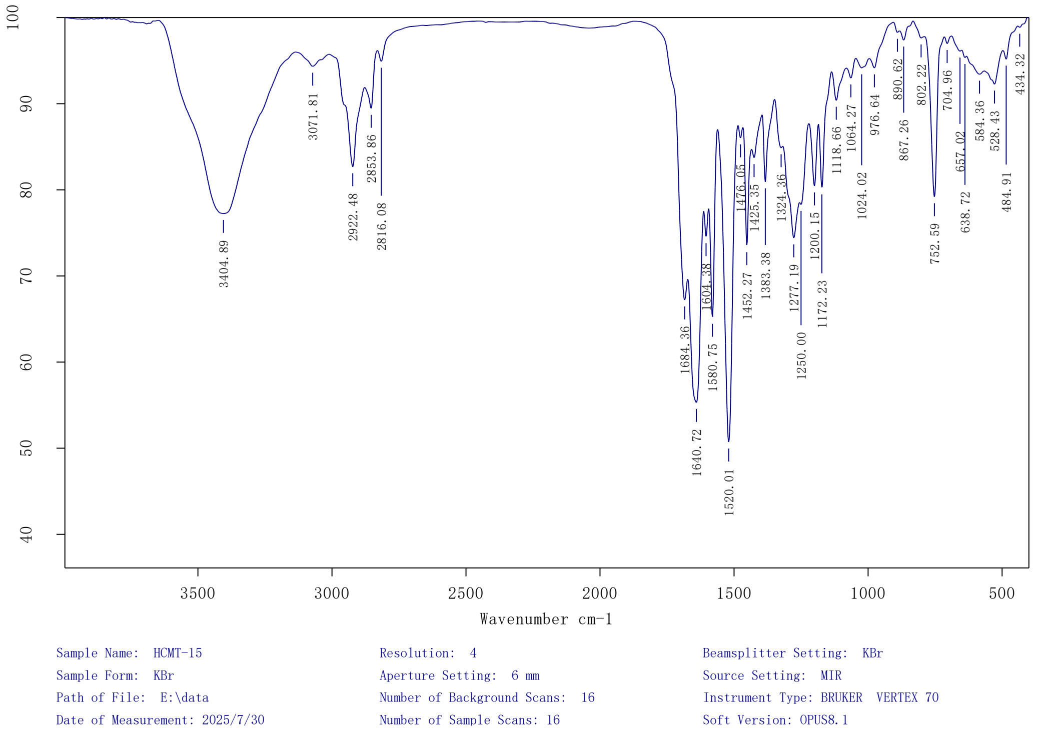

Figure S55. ^{1}H NMR spectrum of compound **30** (600 MHz, $CDCl_3$)

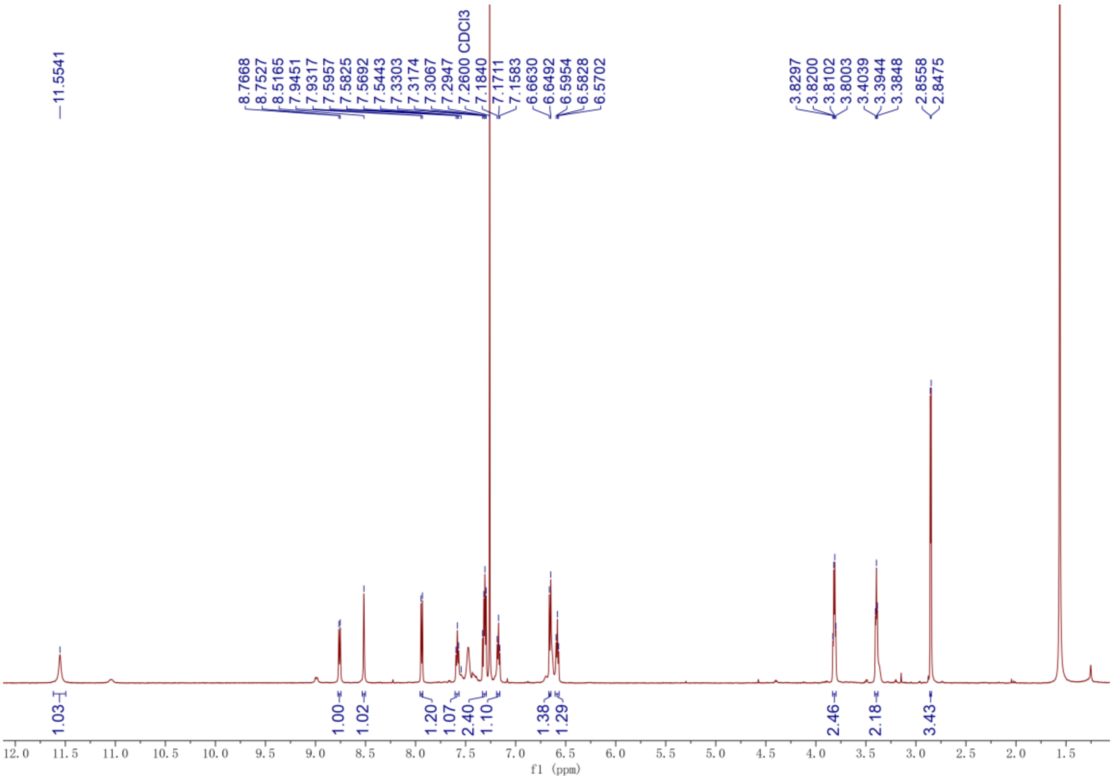

Figure S56. ^{13}C NMR spectrum of compound **30** (150 MHz, $CDCl_3$)

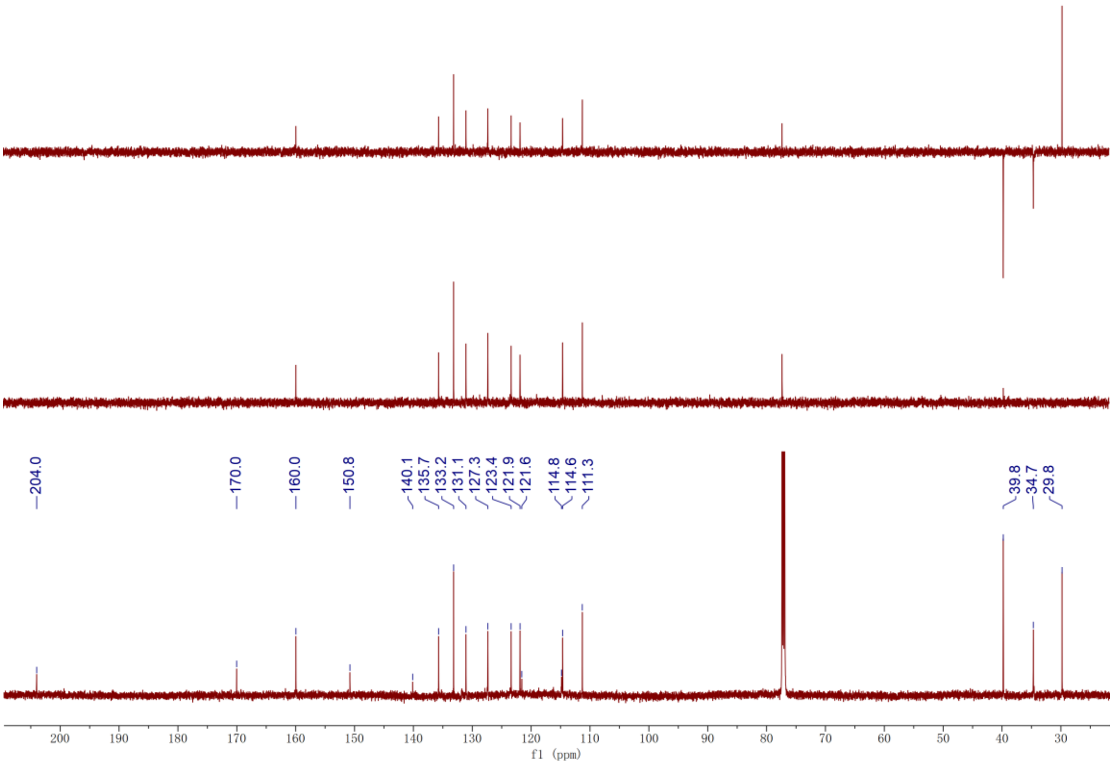

Figure S57. ^{1}H-^{1}H COSY spectrum of compound **30**

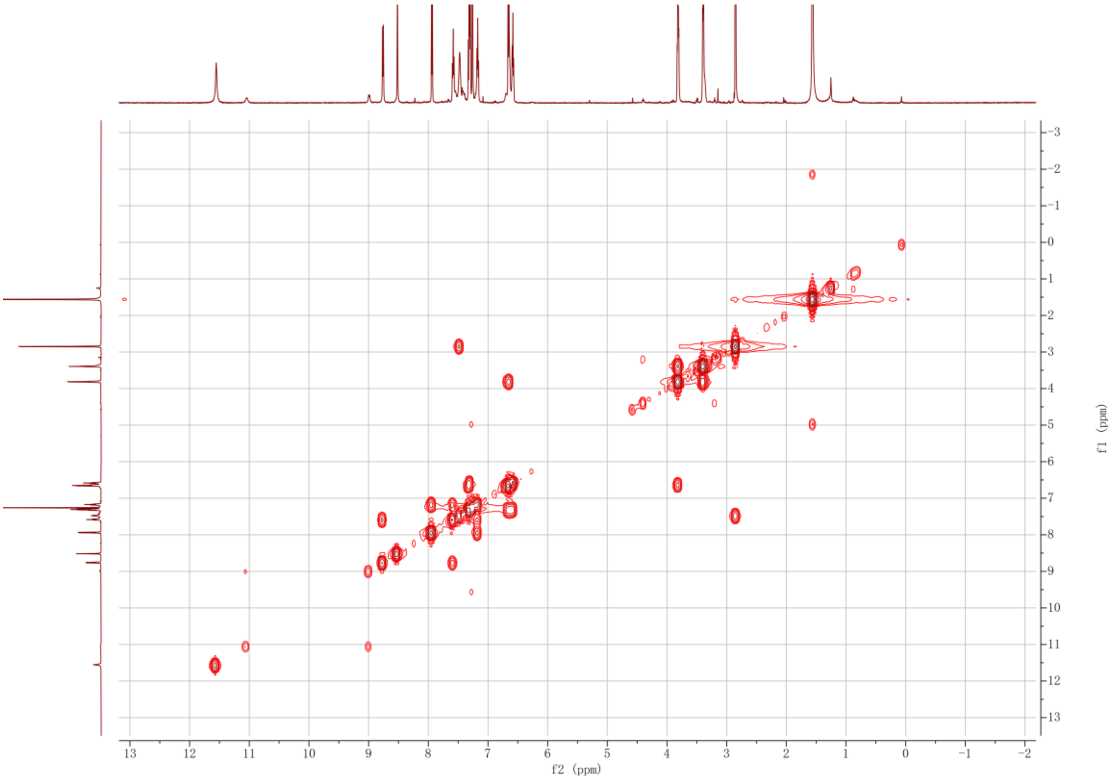

Figure S58. HSQC spectrum of compound **30**

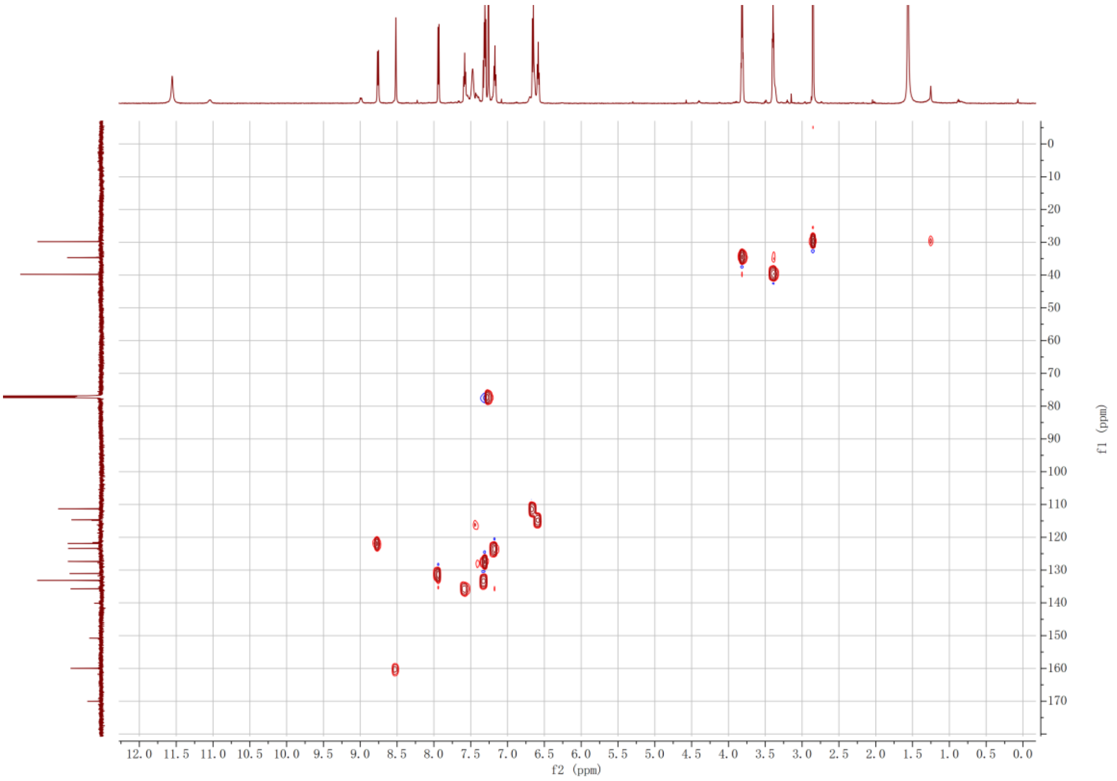

Figure S59. HMBC spectrum of compound **30**

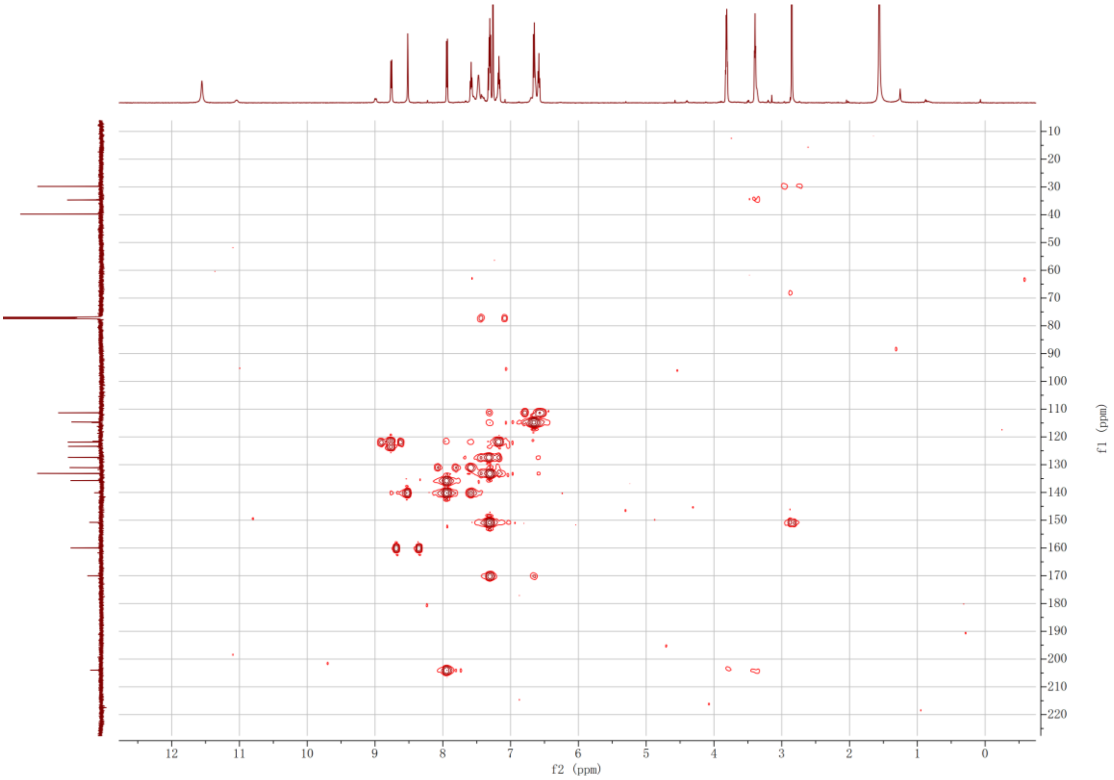

Supplement: Supplementary file 1 [file 13659_2026_610_MOESM1_ESM.pdf]
